# Life-threatening viral disease in a novel form of autosomal recessive *IFNAR2* deficiency in the Arctic

**DOI:** 10.1084/jem.20212427

**Published:** 2022-04-20

**Authors:** Christopher J.A. Duncan, Morten K. Skouboe, Sophie Howarth, Anne K. Hollensen, Rui Chen, Malene L. Børresen, Benjamin J. Thompson, Jarmila Stremenova Spegarova, Catherine F. Hatton, Frederik F. Stæger, Mette K. Andersen, John Whittaker, Søren R. Paludan, Sofie E. Jørgensen, Martin K. Thomsen, Jacob G. Mikkelsen, Carsten Heilmann, Daniela Buhas, Nina F. Øbro, Jakob T. Bay, Hanne V. Marquart, M. Teresa de la Morena, Joseph A. Klejka, Matthew Hirschfeld, Line Borgwardt, Isabel Forss, Tania Masmas, Anja Poulsen, Francisco Noya, Guy Rouleau, Torben Hansen, Sirui Zhou, Anders Albrechtsen, Reza Alizadehfar, Eric J. Allenspach, Sophie Hambleton, Trine H. Mogensen

**Affiliations:** 1 Clinical and Translational Research Institute, Immunity and Inflammation Theme, Newcastle University, Newcastle upon Tyne, UK; 2 The Newcastle upon Tyne Hospitals NHS Foundation Trust, Newcastle upon Tyne, UK; 3 Department of Biomedicine, Aarhus University, Aarhus, Denmark; 4 Department of Infectious Diseases, Aarhus University Hospital, Aarhus, Denmark; 5 Department of Paediatrics and Adolescent Medicine, Copenhagen University Hospital Rigshospitalet, Copenhagen, Denmark; 6 Department of Epidemiology Research, Statens Serum Institut, Copenhagen, Denmark; 7 Section for Computational and RNA Biology, Department of Biology, University of Copenhagen, Copenhagen, Denmark; 8 Novo Nordisk Foundation Center for Basic Metabolic Research, Faculty of Health and Medical Sciences, University of Copenhagen, Copenhagen, Denmark; 9 Medical Department, Pediatric Section, Dronning Ingrid Hospital, Nuuk, Greenland; 10 Division of Genetics, Department of Specialized Medicine, McGill University Health Centre, Montreal, Quebec, Canada; 11 Department of Human Genetics, McGill University, Montreal, Quebec, Canada; 12 Department of Clinical Immunology, Copenhagen University Hospital, Copenhagen, Denmark; 13 Seattle Children’s Hospital, Seattle, WA; 14 Department of Pediatrics, University of Washington, Seattle, WA; 15 Yukon-Kuskokwim Health Corporation, Bethel, AK; 16 Alaska Native Medical Center, Anchorage, AK; 17 Center for Genomic Medicine, Copenhagen University Hospital Rigshospitalet, Copenhagen, Denmark; 18 Division of Allergy & Clinical Immunology, Montreal Children’s Hospital, Montreal General Hospital, McGill University, Montreal, Quebec, Canada; 19 The Neuro, Department of Neurology and Neurosurgery, McGill University, Montreal, Quebec, Canada; 20 Center for Immunity and Immunotherapies, Seattle Children’s Research Institute, Seattle, WA; 21 Brotman Baty Institute for Precision Medicine, Seattle, WA

## Abstract

Type I interferons (IFN-I) play a critical role in human antiviral immunity, as demonstrated by the exceptionally rare deleterious variants of *IFNAR1* or *IFNAR2*. We investigated five children from Greenland, Canada, and Alaska presenting with viral diseases, including life-threatening COVID-19 or influenza, in addition to meningoencephalitis and/or hemophagocytic lymphohistiocytosis following live-attenuated viral vaccination. The affected individuals bore the same homozygous *IFNAR2* c.157T>C, p.Ser53Pro missense variant. Although absent from reference databases, p.Ser53Pro occurred with a minor allele frequency of 0.034 in their Inuit ancestry. The serine to proline substitution prevented cell surface expression of IFNAR2 protein, small amounts of which persisted intracellularly in an aberrantly glycosylated state. Cells exclusively expressing the p.Ser53Pro variant lacked responses to recombinant IFN-I and displayed heightened vulnerability to multiple viruses in vitro—a phenotype rescued by wild-type *IFNAR2* complementation. This novel form of autosomal recessive *IFNAR2* deficiency reinforces the essential role of IFN-I in viral immunity. Further studies are warranted to assess the need for population screening.

## Teaser

The authors report a novel form of autosomal recessive *IFNAR2* deficiency due to a null and common allele in Arctic populations, broadening its clinical phenotype from diseases caused by live-attenuated viral vaccines to include life-threatening influenza and SARS-CoV-2.

## Graphical Abstract

**Figure:**
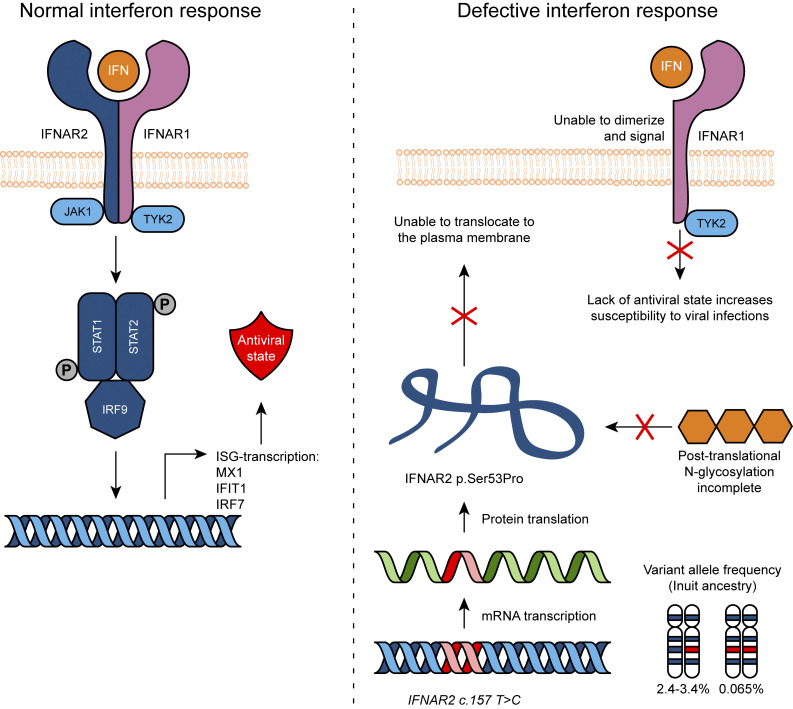

## Introduction

First recognized over 60 yr ago for their capacity to interfere with viral replication (Isaacs and Lindenmann, 1957), type I interferons (IFN-I) are essential for antiviral immunity (reviewed in Duncan et al., 2021). Virtually all human viral pathogens encode molecules designed to evade or subvert IFN-I responses (reviewed in Hoffmann et al., 2015; Randall and Goodbourn, 2008). Much of our understanding of the IFN-I system comes from the study of mice lacking IFN-α/β receptor 1 (*Ifnar1*), which exhibit profound susceptibility to experimental viral challenges and additional defects of immune regulation (Gough et al., 2012; Meyts and Casanova, 2021; Muller et al., 1994). The more recent discovery of viral susceptibility in humans with deleterious variants in the genes encoding IFNAR2 (Bastard et al., 2021b; Duncan et al., 2015; Passarelli et al., 2020) and IFNAR1 (Bastard et al., 2021a; Gothe et al., 2020; Hernandez et al., 2019; Zhang et al., 2020), in addition to anti–IFN-I neutralizing antibodies (Bastard et al., 2020), has shed new light on the specific role of IFN-I in human immunity.

**Figure 1.**
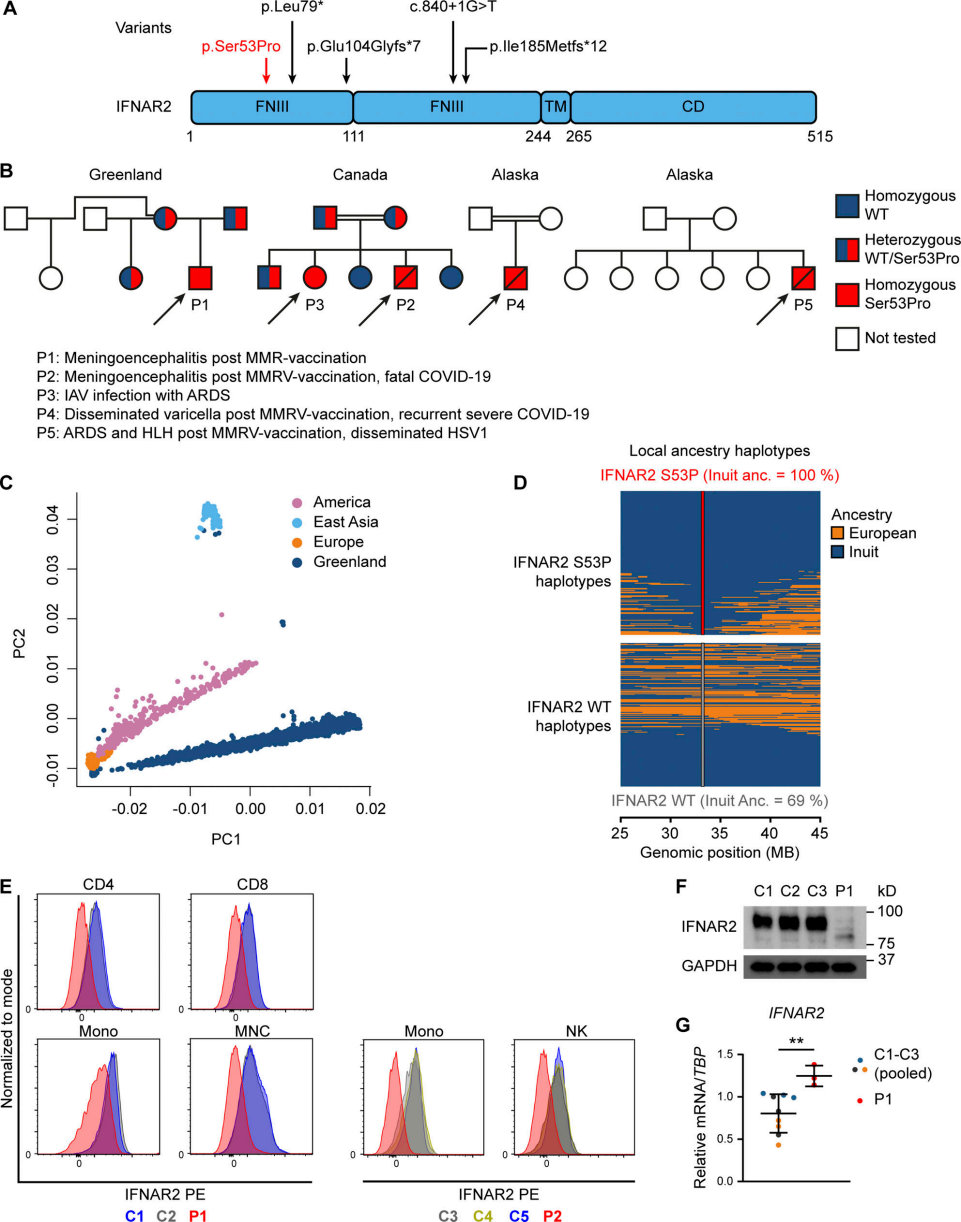
**Identification of a novel homozygous *IFNAR2* variant leading to IFNAR2 deficiency. (A)** IFNAR2 protein sequence with known deleterious variants marked. **(B)** Pedigrees of affected individuals originating from Greenland (P1), Canada (P2 and P3), and Alaska (P4 and P5). Double lines refer to consanguinity. **(C)** Principal component analysis of 4,630 Greenlandic individuals combined with three super populations from the 1000 Genomes Project. **(D)** Local ancestry haplotypes of admixed and p.Ser53Pro *IFNAR2* heterozygous Greenlandic individuals colored according to ancestry. Each heterozygous individual is represented with two haplotypes, one for the p.Ser53Pro haplotype (top) and one for the WT haplotype (bottom). The fraction of Inuit ancestry of the WT and p.Ser53Pro haplotype is estimated at the location of the variant. **(E)** IFNAR2 cell surface expression analyzed by flow cytometry in PBMCs and distinct cell subsets of healthy controls (C1–C5) and patients (P1 and P2). Representative of a single experiment in P1 and P2 PBMC. **(F)** Immunoblotting showing IFNAR2 expression in whole-cell lysates of PBMCs from P1 compared to three healthy controls (C1–C3). GAPDH was used as loading control. Not repeated due to limited patient material. **(G)** RT-qPCR of whole-cell RNA lysates of PBMCs evaluating *IFNAR2* mRNA levels relative to *TBP* for P1 compared to three healthy controls (pooled, mean ± SD of *n* = 3 biological replicates; **, P < 0.01, Welch’s *t* test). Representative of a single experiment in P1 PBMC. Source data are available for this figure: SourceData F1.

IFNAR2 is a ubiquitously expressed transmembrane receptor comprising a heavily N-glycosylated (Ling et al., 1995) extracellular region with two fibronectin domains and a C-terminal cytoplasmic domain that mediates interaction with intracellular signaling molecules, including Janus kinase 1 (JAK1), signal transducer and activator of transcription 2 (STAT2; Fig. 1 A; Li et al., 1997; Piehler et al., 2012). IFNAR2 generally binds IFN-I with greater affinity than IFNAR1 (Jaitin et al., 2006; Lamken et al., 2004). Ligand binding results in the assembly of a ternary complex of IFNAR1:IFNAR2:IFN-I that brings the receptor-associated kinases JAK1 and tyrosine kinase 2 (TYK2) into close proximity, triggering their reciprocal transphosphorylation (Cohen et al., 1995), and subsequent tyrosine phosphorylation of STAT1, STAT2, and other STAT molecules including STAT3, STAT4, and STAT6 (Thomas et al., 2011; van Boxel-Dezaire et al., 2006). In the canonical model, two principal transcription factor complexes assemble, namely IFN-stimulated gene factor 3 (ISGF3, a heterotrimer of pSTAT1, pSTAT2, and IFN regulatory factor 9 [IRF9]; Fu et al., 1992; Levy et al., 1988) and γ-activated factor (GAF, formed of pSTAT1 homodimers; Decker et al., 1991). These, alongside other signaling pathways, engaged downstream of IFNAR, govern the expression of hundreds of IFN-stimulated gene (ISG) products that mediate the biological properties of IFN-I (Der et al., 1998).

Patients with autosomal recessive (AR) deficiency of *IFNAR*2 and subsequently *IFNAR1* were first recognized by their susceptibility to severe viral diseases precipitated by the exposure to live-attenuated viral vaccines (LAV)—specifically, the measles, mumps, and rubella (MMR) and yellow fever vaccines (Bastard et al., 2021b; Duncan et al., 2015; Gothe et al., 2020; Hernandez et al., 2019; Passarelli et al., 2020). Unexpectedly, these individuals appeared otherwise healthy, with no overt evidence of vulnerability to naturally acquired viral pathogens or other immune deficits. This contrasted with patients with AR deficiencies of *STAT1*, *STAT2*, or *IRF9* (Alosaimi et al., 2019; Bravo Garcia-Morato et al., 2019; Chapgier et al., 2009; Chapgier et al., 2006; Dupuis et al., 2003; Freij et al., 2020; Hambleton et al., 2013; Hernandez et al., 2018; Kong et al., 2010; Moens et al., 2017; Shahni et al., 2015; Vairo et al., 2011), all of which are essential downstream components of the IFN-I signaling pathway (John et al., 1991; Kimura et al., 1996; Leung et al., 1995; Meraz et al., 1996; Park et al., 2000; Qureshi et al., 1996; Velazquez et al., 1992). In addition to vulnerability to LAV vaccines (Gothe et al., 2021), these patients experienced recurrent viral diseases due to naturally acquired viral pathogens, presumably due to the added participation of STAT1 and STAT2/IRF9 in signaling from the receptors for type II and/or III IFN (IFN-II/IFN-III; reviewed in Duncan et al., 2021). The pandemic of severe acute respiratory syndrome coronavirus 2 (SARS-CoV-2) has brought renewed focus to the study of the IFN-I system. AR *IFNAR1* deficiency or neutralizing anti–IFN-I autoantibodies confers heightened susceptibility to critical coronavirus disease 2019 (COVID-19) in adults or adolescents (Bastard et al., 2020; Khanmohammadi et al., 2021; Zhang et al., 2020). Furthermore, a recent report identified AR *IFNAR1* deficiency in a child with encephalitis secondary to herpes simplex virus 1 (HSV1; Bastard et al., 2021a). Thus, IFN-I appears to play a nonredundant role in resistance to both WT and attenuated vaccine-strain viruses.

## Results and discussion

### Severe viral disease accompanying MMR(V) vaccination or respiratory virus infection

**Figure S1.**
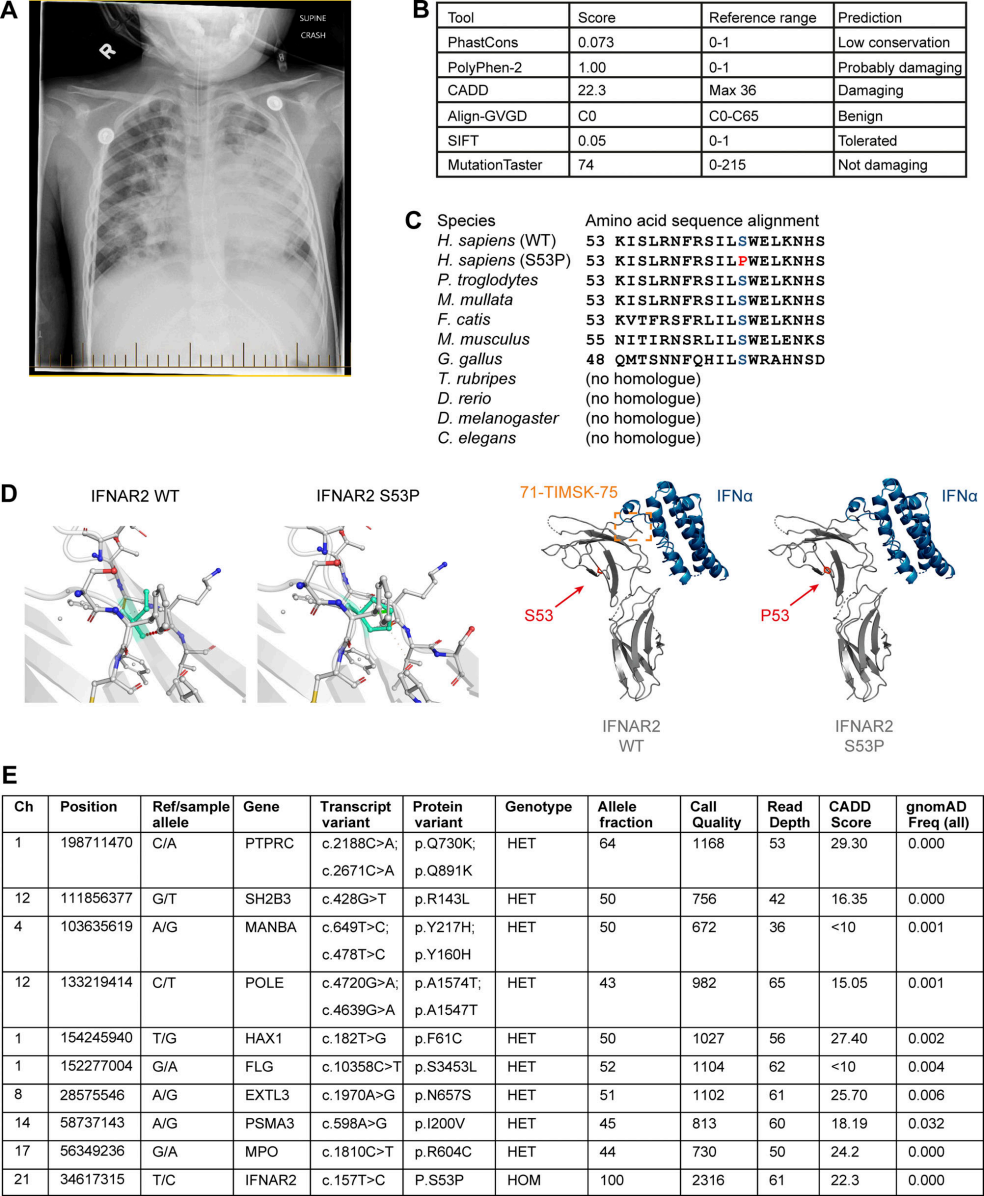
**Clinical information and variant modelling. (A)** Chest radiograph from P3 demonstrating pneumonitis and ARDS associated with influenza A infection. **(B)** Summary of the outputs of in silico prediction software. **(C)** Conservation of the p.Ser53Pro IFNAR2 residue across the indicated species. **(D)** Graphical models of the binary complex between human IFNα2 and IFNAR2 WT and p.Ser53Pro proteins (PDB accession no. 3S9D) were created using PyMOL software version 2.0.7. **(E)** Displayed are all rare exonic variants (gnomAD MAF < 0.01) in genes related to primary immunodeficiency identified by WGS in P1. All genotype quality scores were 99. CADD, Combined Annotation Dependent Depletion.

We investigated five infants of Inuit or Alaska Native heritage living in the circumpolar North, in Greenland (P1), Canada (P2 and P3), and Alaska (P4 and P5). P1 and P2 presented with meningoencephalitis in temporal association with the receipt of live-attenuated MMR (+/− varicella; MMR[V]) vaccine according to national immunization schedules. In P2, illness was associated with systemic hyperinflammation, although it did not reach the diagnostic criteria for hemophagocytic lymphohistiocytosis (HLH). Both recovered from their index illness with corticosteroid treatment. P2, aged nearly 3 yr, subsequently developed severe and ultimately fatal COVID-19. P3, the older sister of P2, did not fall ill following MMRV, but developed life-threatening illness after influenza A virus (IAV) infection at the age of 5 yr with acute respiratory distress syndrome (ARDS; Fig. S1 A) requiring mechanical ventilation. She later experienced SARS-CoV-2 infection aged 7 yr, coincident with her brother, but did not progress to develop severe disease. P4 developed disseminated varicella 30 d after the receipt of MMRV at the age of 14 mo, having a background of a previously diagnosed progressive neurodegenerative disorder of unknown etiology and recent hospitalization with IAV infection and associated pneumonia. Virological sequencing confirmed the vaccine-strain varicella zoster virus (VZV) that was present in the vesicular fluid, blood, and bronchoalveolar lavage (BAL), but not cerebrospinal fluid (CSF). She recovered back to her baseline following treatment with i.v. acyclovir, but subsequently experienced recurrent severe COVID-19, leading to two hospital admissions. Care was subsequently moved to a palliative footing owing to the severity of the neurological disease and she succumbed at the age of 3 yr. P5 was admitted to his local hospital with fever, conjunctivitis, and pneumonitis, 4 d after the first dose of MMRV at 13 mo of age. His clinical condition deteriorated significantly due to life-threatening respiratory failure secondary to ARDS, accompanied by HLH, which was ultimately fatal despite extracorporeal membrane oxygenation, anakinra, and dexamethasone. Although the precise virological trigger for this illness was uncertain, there was molecular evidence of systemic dissemination of vaccine-strain MMR viruses in addition to an extremely high copy number HSV1 viremia and detection of a range of additional viruses, including respiratory syncytial virus (RSV), adenovirus, CMV, EBV, and human herpes virus 6 (HHV6) in BAL, suggestive of a profound defect in antiviral immunity. There was no response to combination antiviral therapy with cidofovir, ribavirin, acyclovir, and intravenous immunoglobulin (IVIG). P5 was the youngest of six children, and all older siblings are reportedly healthy; genetic testing of the family has not yet been undertaken. Further clinical details of these patients are included in the Materials and methods.

### Identification of a novel missense p.Ser53Pro variant in *IFNAR2* associated with Inuit ancestry

Independent genetic investigation was undertaken by whole-genome sequencing (WGS; P1), commercial targeted whole-exome sequencing (WES; P2–4), or panel sequencing (P5), and a subsequent analysis strategy focusing on genes related to primary immune deficiency. The same homozygous single nucleotide variant in *IFNAR2*, predicted to produce a missense substitution of serine with proline at position 53 (c.157T>C, p.Ser53Pro, rs1987287426), was identified in all five affected individuals (Fig. 1 A). Where available, genetic segregation studies in the affected families were consistent with an AR inheritance pattern (Fig. 1 B). The *IFNAR2* p.Ser53Pro variant was absent from the gnomAD database of population genetic variation (Karczewski et al., 2020). The site has both high coverages in gnomAD and was not filtered out, suggesting that it is not polymorphic in the queried populations. In silico predictions of functional impact were inconsistent (Fig. S1 B). Ser53 is a highly conserved residue across species (Fig. S1 C), located in the extracellular fibronectin domain of IFNAR2, separate from the IFN-I binding site (Figs. 1 A and S1 D). Structural modeling (Protein Data Bank [PDB] accession no. 1N6V) revealed changes to local hydrogen bonds introduced by the substitution of proline (Fig. S1 D). No additional relevant variants were identified in P1 by WGS (Fig. S1 E). Considering the sparse representation of genomic data from the relevant populations in gnomAD, we sought alternative sources of information to obtain a better population genetic understanding of the variant.

In unpublished WGS data obtained from 448 healthy individuals in Greenland, the p.Ser53Pro variant was identified in heterozygosity in 23 persons with no homozygous carriers, giving a minor allele frequency (MAF) of 0.026. We next used dense single nucleotide polymorphism (SNP) chip data of 4,182 adults from Greenland (Bjerregaard, 2011; Bjerregaard et al., 2003). These were merged with the unpublished whole-genome reference panel described above and imputed resulting in a final data set consisting of 4,630 individuals. Based on the merged and imputed data of these 4,630 Greenlandic individuals, we performed principal component analysis combined with three of the super populations from the 1000 Genomes Project (Fig. 1 C). This revealed that the Greenlanders were genetically distinct from both East Asians and the populations from the Americas. However, most Greenlanders were admixed with Europeans resulting in an observed gradient toward the European superpopulation. A similar pattern was also observed for individuals from the Americas. Note that a few Greenlanders had apparent East Asian ancestry and hence did not cluster with the Greenlandic population. These individuals were removed when estimating the allele frequencies of the variant within the Inuit ancestry.

From the remaining 4,619 Greenlandic individuals, we identified 220 heterozygous and three homozygous carriers yielding an overall MAF of 0.024 (imputation confidence 0.987). To assess the distribution of the p.Ser53Pro variant, we performed a local ancestry analysis where the phased haplotypes for each individual are colored according to their European or Inuit ancestry. The p.Ser53Pro allele was exclusively found on a background of Inuit ancestry, whereas the reference allele was identified on both European and Inuit backgrounds (Fig. 1 D). Ancestry-specific allele frequencies were 0.034 and 0.000 for the Inuit and European ancestries, respectively. Consistent with these findings, we undertook a separate analysis of a published WES dataset from 104 healthy individuals of Inuit heritage from Nunavik, Canada (Zhou et al., 2019). The p.Ser53Pro allele was present in the heterozygous state in seven individuals (MAF 0.034). Excluding related individuals (defined as second degree or closer) gave a slightly higher MAF of 0.047. Collectively, these data indicated that the p.Ser53Pro *IFNAR2* variant was present at a relatively high allele frequency in association with Inuit ancestry.

### Patient leukocytes do not express IFNAR2 on the cell surface

**Figure S2.**
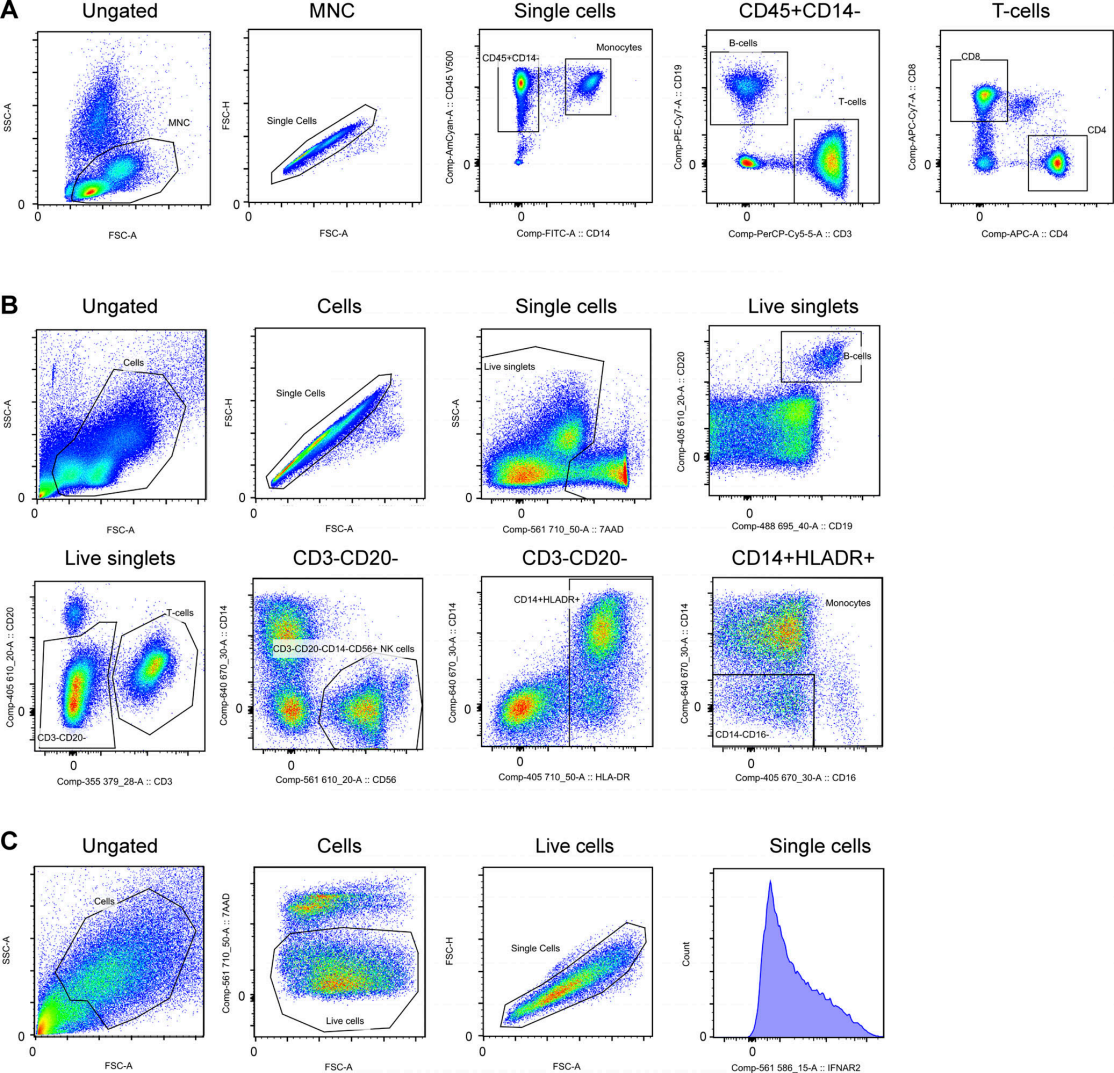
**Flow cytometry gating strategy. (A and B)** Gating strategy for Fig. 1 E, P1 (A) and P2 (B) alongside *n* = 2–3 healthy controls. **(C)** Gating strategy for Fig. 4 G.

To assess the pathogenicity of the p.Ser53Pro *IFNAR2* variant, we first analyzed IFNAR2 surface expression on peripheral blood mononuclear cells (PBMC) from P1 and P2 (Fig. 1 E; gating strategy in Fig. S2, A and B) by flow cytometry. The surface expression of IFNAR2 was lacking from major leukocyte cell subsets, including T cells, monocytes, and natural killer (NK) cells. Immunoblotting of whole-cell lysates prepared from PBMC of P1 also showed markedly reduced expression of a faster migrating IFNAR2 protein (Fig. 1 F). The analysis of *IFNAR2* mRNA expression in PBMC from P1 alongside healthy controls (Fig. 1 G) demonstrated normal *IFNAR2* mRNA abundance, consistent with the lack of a predicted impact on splicing or mRNA stability. These findings pointed to a major defect of IFNAR2 protein expression, predicted to compromise the cellular response to IFN-I.

### Cells bearing p.Ser53Pro IFNAR2 in the homozygous state are unresponsive to IFN-I

**Figure 2.**
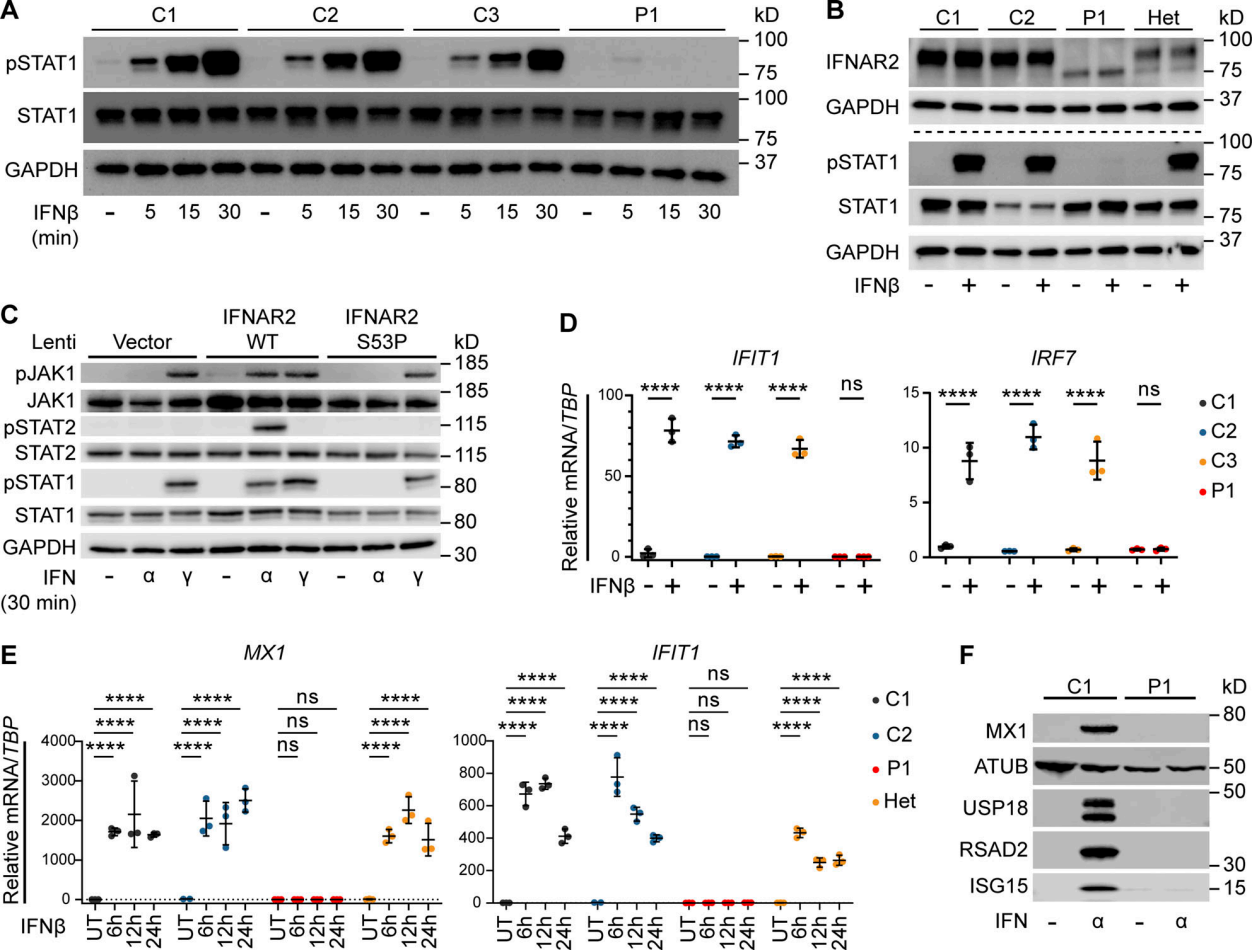
**Defective signaling and ISG induction downstream of IFNAR2 p.Ser53Pro. (A)** PBMCs from P1 and three healthy controls (C1–C3) were treated with IFNβ 100 IU/ml for 5, 15, or 30 min. Whole-cell lysates were harvested for Western blotting for the visualization of pSTAT1, STAT1, and GAPDH, which was used as the loading control. Not repeated due to limited patient material. **(B)** Primary dermal fibroblasts from P1, two healthy controls (C1 and C2), and the heterozygous mother of P1 (Het) were treated with IFNβ 100 IU/ml for 30 min. Whole-cell lysates were harvested for immunoblotting and the visualization of IFNAR2, STAT1, pSTAT1, and GAPDH as the loading control. One representative immunoblot of *n* = 3 independent experiments is shown. **(C)** Primary *IFNAR2*^−/−^ dermal fibroblasts reconstituted with lentiviruses expressing GFP, WT, or p.Ser53Pro *IFNAR2* were treated with IFNα2b or IFNγ 1,000 IU/ml for 30 min. Whole-cell lysates were harvested for immunoblotting and visualization of pJAK1, JAK1, pSTAT2, STAT2, STAT1, pSTAT1, and GAPDH as the loading control. One representative immunoblot of *n* = 3 independent experiments is shown. **(D)** PBMCs from P1 and three healthy controls (C1–C3) were treated with IFNβ 100 IU/ml for 6 h. Total RNA was purified for RT-qPCR of *IFIT1* and *IRF7* relative to *TBP* (mean ± SD of *n* = 3 independent experiments; ****, P < 0.0001, ns, non-significant; two-way ANOVA with Šidák’s test for multiple comparisons). **(E)** Primary dermal fibroblasts from P1, two healthy controls (C1 and C2), and the mother of P1 (Het) were treated with IFNβ 100 IU/ml for 6–24 h. Total RNA was purified for RT-qPCR of *MX1* and *IFIT1* relative to *TBP* (mean ± SD of *n* = 3 independent experiments; ****, P < 0.0001; ns, non-significant; two-way ANOVA with Dunnett’s test for multiple comparisons). **(F)** Primary dermal fibroblasts from P1 or a healthy control (C1) were treated with IFNα2b 1,000 IU/ml overnight. Whole-cell lysates were prepared for immunoblotting and visualization of MX1, USP18, RSAD2, ISG15, and α-tubulin (ATUB) as the loading control. One representative immunoblot of *n* = 3 independent experiments is shown. Source data are available for this figure: SourceData F2.

**Figure S3.**
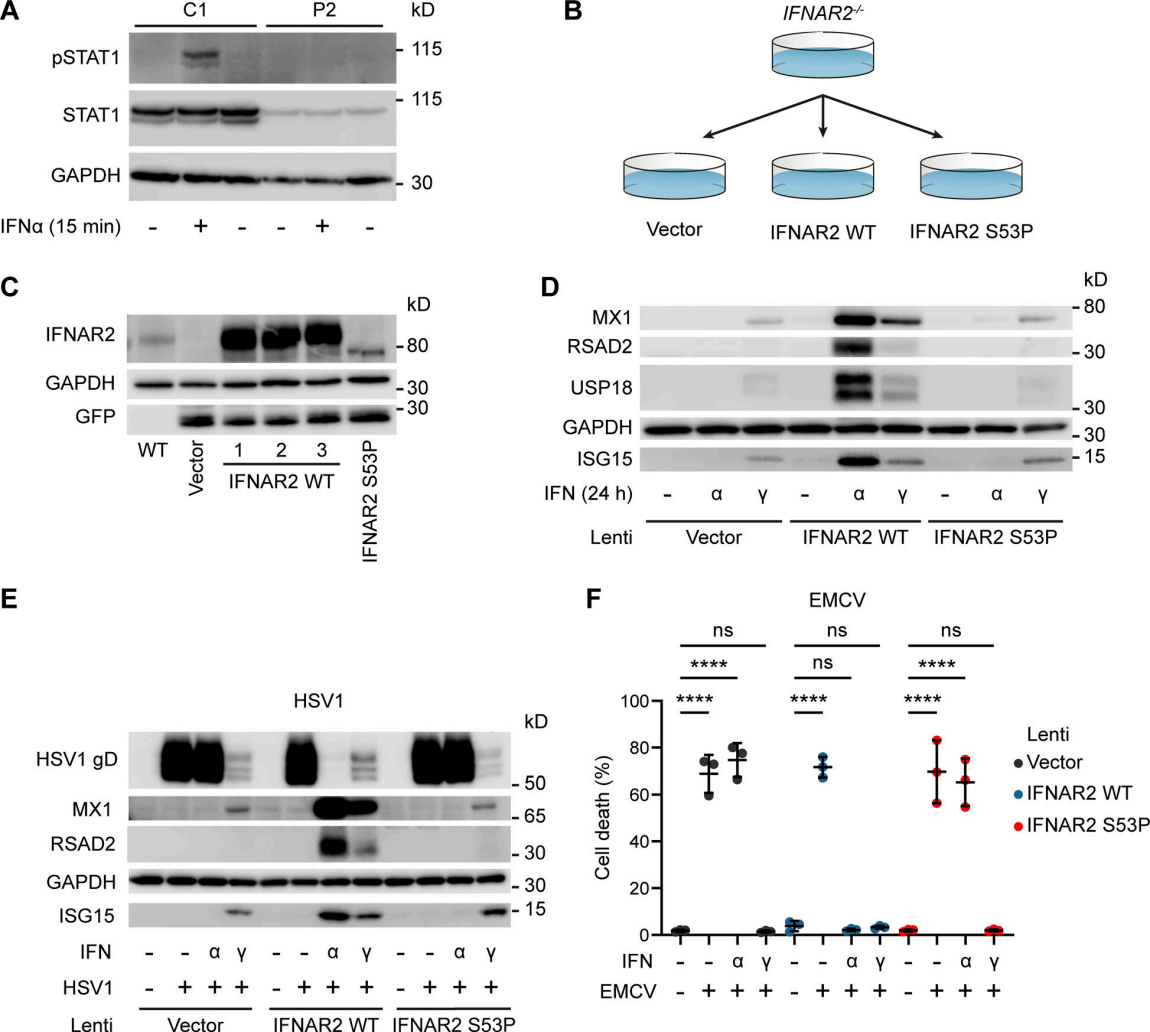
**In vitro functional modelling of the IFNAR2 p.Ser53Pro mutant. (A)** EBV-transformed B cells from P2 and a healthy control (C1) were treated with IFNα2b 1,000 IU/ml for 30 min. Whole cell lysates were harvested for immunoblotting and visualization of STAT1, pSTAT1 and GAPDH as loading control. Representative of *n* = 2 repeat experiments. **(B)** Graphical summary of experimental strategy. IFNAR2-deficient dermal fibroblasts were reconstituted with WT or mutant IFNAR2 lentiviral constructs or an empty vector control. **(C)** IFNAR2-deficient dermal fibroblasts were reconstituted with WT or mutant *IFNAR2* lentiviral constructs or an empty vector control and protein lysates prepared for immunoblotting for IFNAR2, GFP, and GAPDH loading control. Representative of *n* = 3 independent experiments. **(D)** IFNAR2-deficient dermal fibroblasts were reconstituted with WT or mutant *IFNAR2* lentiviral constructs or an empty vector control. After overnight stimulation with IFNα2b or IFNγ (1,000 IU/ml) protein lysates were prepared for immunoblotting for the ISG products MX1, USP18, RSAD2 and ISG15 alongside GAPDH loading control. Representative of *n* = 3 independent experiments. **(E)** Primary IFNAR2-deficient fibroblasts, stably reconstituted with empty vector, WT *IFNAR2* or p.Ser53Pro *IFNAR2*, were pre-treated with IFNα2b or IFNγ (1,000 IU/ml) overnight, prior to infection with HSV1 (17 + strain, MOI 0.01). At 48 h after infection, lysates were prepared for immunoblotting for HSV gD, alongside MX1, RSAD2, ISG15 and GAPDH as loading control. Representative of *n* = 3 independent experiments. **(F)** Primary IFNAR2-deficient fibroblasts, stably reconstituted with empty vector, WT *IFNAR2* or p.Ser53Pro *IFNAR2*, were pre-treated with IFNα2b or IFNγ (1,000 IU/ml) overnight, before infection with a cytopathic dose of EMCV. At 24 h after infection, cell viability was assessed in an imaging based live cell viability assay (mean ± SD of *n* = 3 independent experiments, one-way ANOVA with Šidák’s multiple comparisons test; ****, P < 0.0001; ns, non-significant). Source data are available for this figure: SourceData FS3.

To test this prediction, we treated PBMC from P1 with recombinant IFNβ (100 IU/ml) for 5–30 min before preparing whole cell lysates and assessing tyrosine phosphorylation of STAT1 by immunoblotting. This analysis revealed the absence of STAT1 phosphorylation in cells from P1, but not healthy controls, consistent with defective IFNAR signaling (Fig. 2 A). Additional studies examining STAT1 phosphorylation in dermal fibroblasts confirmed this defect of signaling in cells from P1 but not the heterozygous parent (Fig. 2B), as did studies of EBV-transformed B cells from P2 (Fig. S3 A). To verify these findings, we complemented IFNAR2-deficient fibroblasts (Duncan et al., 2015) with either WT or p.Ser53Pro *IFNAR2* via lentiviral transduction (Fig. S3, B and C) observing the failure of the latter to restore tyrosine phosphorylation of JAK1, STAT1, or STAT2 in response to IFN-I, but preserved responses to IFN-II, indicative of a specific defect of IFNAR signaling (Fig. 2 C).

We next considered the consequences for ISG expression. In PBMC from P1, the defect of JAK-STAT signaling correlated with a failure to upregulate ISGs, as assessed by RT-qPCR in PBMC (Fig. 2 D) or the fibroblasts (Fig. 2 E). Consistent with this, immunoblotting of lysates prepared from P1 fibroblasts (Fig. 2 F) or p.Ser53Pro *IFNAR2* reconstituted fibroblasts (Fig. S3 D) treated overnight with IFNα2b (1,000 IU/ml) confirmed the absence of ISG expression at the protein level. Collectively, these data indicated a complete failure of the response to IFN-I in cells bearing p.Ser53Pro *IFNAR2* in homozygosity.

### Defective induction of antiviral state in p.Ser53Pro homozygous cells

**Figure 3.**
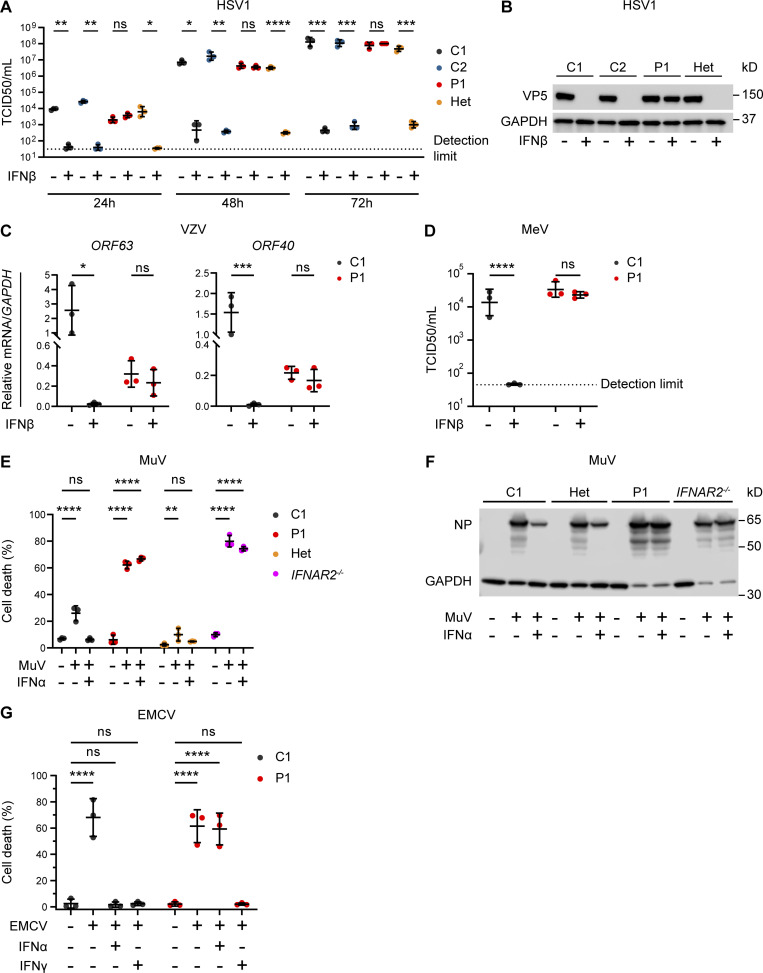
**Impaired viral control in patient fibroblasts bearing homozygous *IFNAR2* p.Ser53Pro. (A and B)** SV40-immortalized dermal fibroblasts from P1, two healthy controls (C1 and C2), and the heterozygous mother of P1 (Het) were pretreated with IFNβ 100 IU/ml for 24 h before infection with HSV1 (KOS strain) at an MOI of 0.001. **(A)** At 24, 48, and 72 hpi, supernatants were sampled and titrated for TCID50 (geometric mean ± SD of *n* = 3 independent repeats; *, P < 0.05; **, P < 0.01; ***, P < 0.001; ****, P < 0.0001, two-way ANOVA with Tukey’s test for multiple comparisons). **(B)** At 72 hpi, the cells were lysed for immunoblotting of whole-cell lysates for the HSV1 protein VP5 and GAPDH as the loading control. One representative immunoblot of *n* = 3 independent repeat experiments is shown. **(C)** SV40-immortalized dermal fibroblasts from P1 and a healthy control were pretreated with IFNβ 100 IU/ml for 24 h before infection with cell-free VZV at an MOI of 1. At 48 hpi, total RNA was harvested for RT-qPCR, evaluating the levels of VZV immediate early *ORF63* and late *ORF40* transcripts, respectively, relative to *GAPDH* (mean ± SD of *n* = 3 independent repeats; *, P < 0.05; ***, P < 0.001, two-way ANOVA with Šidák’s test for multiple comparisons). **(D)** SV40-immortalized dermal fibroblasts from P1 and one healthy control were pretreated with IFNβ 100 IU/ml for 24 h before infection with MeV (Edmonston strain, MOI = 0.00083). At 96 hpi, supernatants were harvested and titrated for TCID50 (geometric mean ± SD of *n* = 3 independent repeats; ****, P < 0.0001, two-way ANOVA with Tukey’s test for multiple comparisons). **(E and F)** Primary dermal fibroblasts from P1, a healthy control (C1), the heterozygous mother of P1 (Het) and a known IFNAR2-deficient patient (*IFNAR2*^−/−^) were pretreated with IFNα2b 1,000 IU/ml for 16 h before infection with MuV at an MOI 0.1 (MuV, Enders strain). At 72 hpi, (E) viability was assessed in an imaging based live cell viability assay (mean ± SD of *n* = 3 independent repeats; **, P < 0.01; ****, P < 0.0001, two-way ANOVA with Dunnett’s test for multiple comparisons) and (F) whole cell lysates were prepared for immunoblotting and visualization of MuV nucleoprotein (NP) and GAPDH as loading control. One representative immunoblot of *n* = 3 independent repeat experiments is shown. **(G)** Primary dermal fibroblasts from P1 and a healthy control (C1) were pretreated with IFNα2b 1,000 IU/ml for 16 h before infection with a cytopathic dose of EMCV. At 24 hpi, cell viability was assessed in an imaging based live cell viability assay (mean ± SD of *n* = 3 independent repeats; ****, P < 0.0001, two-way ANOVA with Dunnett’s test for multiple comparisons). Source data are available for this figure: SourceData F3.

To explore the functional impact of this defect, we investigated the ability of P1 dermal fibroblasts to mount an IFN-I–mediated antiviral state. For these experiments, we examined the viral pathogens relevant to the clinical phenotype of the patients, including measles virus (MeV) and mumps virus (MuV), in addition to HSV1 and VZV. We initially explored the induction of the antiviral state to IFNβ in simian virus 40 (SV40)–transformed fibroblasts from P1, the heterozygous parent, and two controls challenged with HSV1 KOS strain at a multiplicity of infection (MOI) of 0.001. Patient cells completely failed to adopt an antiviral state in response to IFNβ, whereas cells from the controls or heterozygous parent were protected (Fig. 3, A and B). Consistent with this, fibroblasts from P1 also demonstrated a defect of antiviral protection against rOka-strain VZV (Fig. 3 C), Edmonston-strain MeV (Fig. 3 D), or Enders-strain MuV (Fig. 3 E), all vaccine strains. Compatible findings were observed in primary *IFNAR2*-deficient dermal fibroblasts (Duncan et al., 2015) reconstituted with p.Ser53Pro but not WT *IFNAR2* (Fig. S3, E and F). In these experiments, we observed that IFNγ pre-treatment conferred an antiviral state upon p.Ser53Pro *IFNAR2* complemented cells, as was also seen in primary dermal fibroblasts from P1 (Fig. 3 G). Collectively, these data indicated that the p.Ser53Pro *IFNAR2* allele was functionally null, consistent with flow studies in patient PBMC showing that p.Ser53Pro IFNAR2 protein was not expressed at the cell surface (Fig. 1 E). Next, we considered the underlying molecular mechanisms.

### p.Ser53Pro IFNAR2 is an unstable, aberrantly N-glycosylated protein that fails to traffic through the secretory pathway to the cell surface

**Figure 4.**
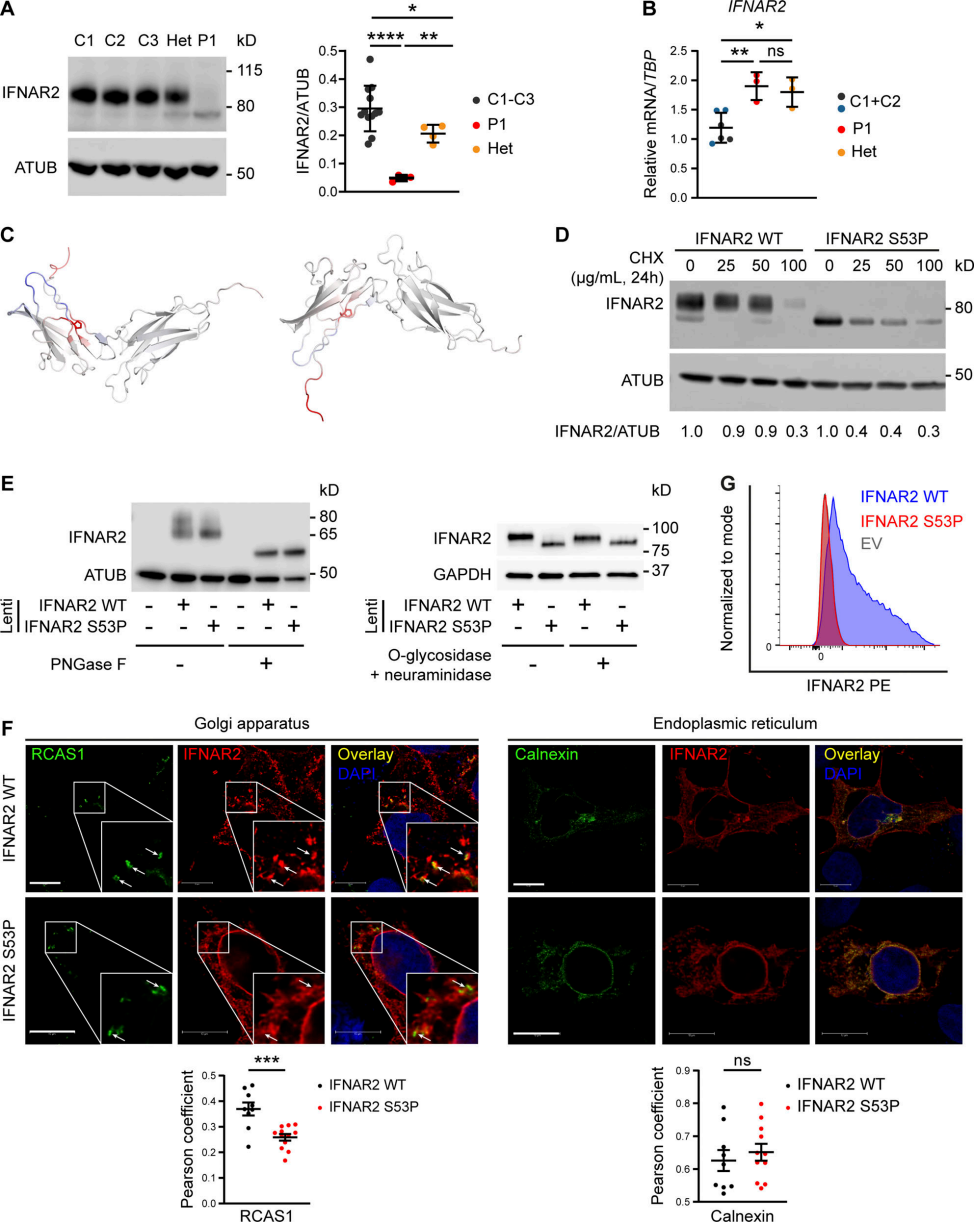
**Defective processing of p.Ser53Pro IFNAR2 through the secretory pathway. (A)** Primary dermal fibroblasts from P1, healthy controls (C1–3), and the heterozygous mother of P1 (Het) were lysed for immunoblotting of whole cell lysates for IFNAR2 protein and α-tubulin (ATUB) as loading control. Densitometry analysis of IFNAR2 expression relative to loading control (mean ± SD of *n* = 3 independent experiments; *, P < 0.05; **, P < 0.01; ****, P < 0.0001, Welch’s one-way ANOVA with Dunnett’s test for multiple comparisons). **(B)** Total RNA from primary dermal fibroblasts from P1, two healthy controls (C1 and C2), and the heterozygous mother of P1 (Het) was purified for RT-qPCR evaluating *IFNAR2* mRNA levels relative to *TBP* (mean ± SD of *n* = 3 independent experiments; *, P < 0.05; **, P < 0.01, one-way ANOVA with Tukey’s test for multiple comparisons). **(C)** Model of change to IFNAR2 protein entropy from the p.Ser53Pro substitution, prepared using DynaMUT. Red color indicates a gain of flexibility, blue a gain of stability. **(D)** HEK 293 FT cells were transfected with HA-tagged expression constructs encoding WT or p.Ser53Pro *IFNAR2*. 24 h after transfection, the cells were treated with CHX at the indicated concentrations or DMSO vehicle control, for a further 24 h, before whole-cell lysates were prepared for immunoblotting for IFNAR2, and α-tubulin (ATUB) as loading control. Expression of IFNAR2 protein relative to loading control was assessed by densitometry analysis and expressed as a proportion of the DMSO-treated control. Displayed is a representative immunoblot of two independent experiments. **(E)** Left: HEK293FT cells were transfected with lentiviral expression constructs expressing *IFNAR2* WT or *IFNAR2* p.Ser53Pro or empty vector. Whole cell lysates were prepared and treated with PNGase F before immunoblotting for IFNAR2 and GAPDH as loading control. Displayed is a representative immunoblot of *n* = 3 independent experiments. **(E)** Right: Primary dermal fibroblasts of a healthy control were stably transduced with a lentiviral vector encoding *IFNAR2* WT or *IFNAR2* p.Ser53Pro. Whole cell protein lysates were harvested and treated with O-glycosidase and neuraminidase before immunoblotting for IFNAR2 and GAPDH as loading control. Displayed is a representative immunoblot of *n* = 2 independent experiments. **(F)** HEK293FT cells were transfected with HA-tagged expression constructs encoding WT or p.Ser53Pro *IFNAR2*. 24 h after transfection cells were fixed, immunostained, and imaged by confocal microscopy for expression of the Golgi marker RCAS1 (left) or the ER marker calnexin (right) alongside IFNAR2. Scale bars represent 10 μm. Shown are the results of correlation analysis of IFNAR2 and the relevant organelle marker in individual cells (mean ± SD of *n* = 9 WT and *n* = 12 S53P cells analyzed; ***, P < 0.001, *t* test). Representative of *n* = 2 repeat experiments. **(G)** HEK293FT cells were transfected with HA-tagged expression constructs encoding WT or p.Ser53Pro *IFNAR2*. 24 h after transfection cell surface expression of IFNAR2 was assessed by flow cytometry. Representative of *n* = 2 repeat experiments. Source data are available for this figure: SourceData F4.

As observed in PBMC, p.Ser53Pro IFNAR2 protein was expressed at a lower level in dermal fibroblasts from P1 (Fig. 4 A) and migrated at a lower apparent molecular weight than WT IFNAR2, while mRNA expression was preserved (Fig. 4 B), indicating that the defect of IFNAR2 protein expression arose following the transcription. The S53 residue is buried within the N-terminal extracellular domain of IFNAR2, away from the ligand-binding site (Fig. S1 D). Considering that the introduction of proline may have an impact on protein stability, we performed an in silico analysis of protein-free energy changes (ΔΔGG) using PremPS (Chen et al., 2020), which revealed a ΔΔGG value of 1.22 kcal/mol, consistent with impaired stability. Additional analysis of the change in vibrational entropy energy between WT and p.Ser53Pro using DynaMUT (Rodrigues et al., 2018) revealed an ΔΔSVib ENCoM value of 0.431 kcal/mol/K, indicative of a net gain of flexibility (Fig. 4 C). We investigated these predictions experimentally by transfecting HEK293FT cells with WT or p.Ser53Pro *IFNAR2* for 24 h before treating the cells with cycloheximide (CHX) at a range of doses to terminate the transcription. Immunoblot analysis after 24 h of CHX treatment revealed a greater loss of p.Ser53Pro IFNAR2 protein abundance compared with WT IFNAR2, consistent with the reduced stability of p.Ser53Pro IFNAR2 protein (Fig. 4 D).

We next considered the explanation for the faster migration of p.Ser53Pro IFNAR2 as compared with WT IFNAR2 on SDS-PAGE and immunoblotting. WT IFNAR2 is known to be heavily N-glycosylated (Ling et al., 1995), accounting for its apparent molecular weight (MW) of ∼80–90 kD compared with the predicted MW of 57 kD based on protein sequence, as noted previously (Duncan et al., 2015; Ling et al., 1995). We, therefore, tested the hypothesis that altered glycosylation accounted for the difference in electrophoretic mobility between WT and p.Ser53Pro IFNAR2. The treatment of protein lysates with peptide:N-glycosidase F (PNGase F), which cleaves virtually all N-linked glycans from glycoproteins, indeed resulted in the faster migration of WT and p.Ser53Pro bands, but at the same time predicted a MW of ∼57 kD based on protein sequence, indicating that the difference in N-glycosylation, not protein sequence, accounted for the prior differences in migration (Fig. 4 E). Furthermore, O-glycosidase treatment of lysates, which strips O-linked glycans, had no effect on IFNAR2 migration, consistent with the paucity of O-linked glycans in IFNAR2 (Fig. 4 E). Collectively these data confirm that the difference in migration between WT and p.Ser53Pro IFNAR2 was due to altered N-glycosylation rather than a change in protein mass per se. Interestingly, the lower band was also detected alongside the larger upper band in HEK293FT lysates following transfection with WT IFNAR2, suggesting the faster migrating species to be an immature glycoform (Fig. 4, D and E).

Cell surface glycoproteins such as IFNAR2 are processed in the secretory pathway. N-glycosylation is initiated in the ER, and N-glycans are further modified in the Golgi apparatus before proteins are trafficked to the cell surface. The aberrant N-glycosylation profile and reduction in cell surface expression suggested that progress of p.Ser53Pro IFNAR2 protein through the secretory pathway was impaired. To assess this possibility, we used confocal microscopy to examine the subcellular colocalization of transfected IFNAR2 with markers of the ER (calnexin) or the Golgi (receptor binding cancer antigen expressed on SiSo cells, RCAS1; Fig. 4 F). IFNAR2 p.Ser53Pro was significantly less associated with the Golgi marker RCAS1 than WT IFNAR2, suggesting that less mutant IFNAR2 was reaching the Golgi. Both the proteins were highly associated with the ER marker calnexin, with no significant differences in the Pearson correlation coefficient. Consistent with this, we failed to detect the cell surface expression of p.Ser53Pro IFNAR2 in transfected cells by flow cytometry, despite normal expression of WT IFNAR2 (Fig. 4 G, gating strategy Fig. S2 C). These data demonstrate that the proline substitution destabilized IFNAR2, retarding its progression through the secretory pathway and compromising cell surface expression.

### Complementation with WT *IFNAR2* restores IFNAR2 expression and antiviral function

**Figure 5.**
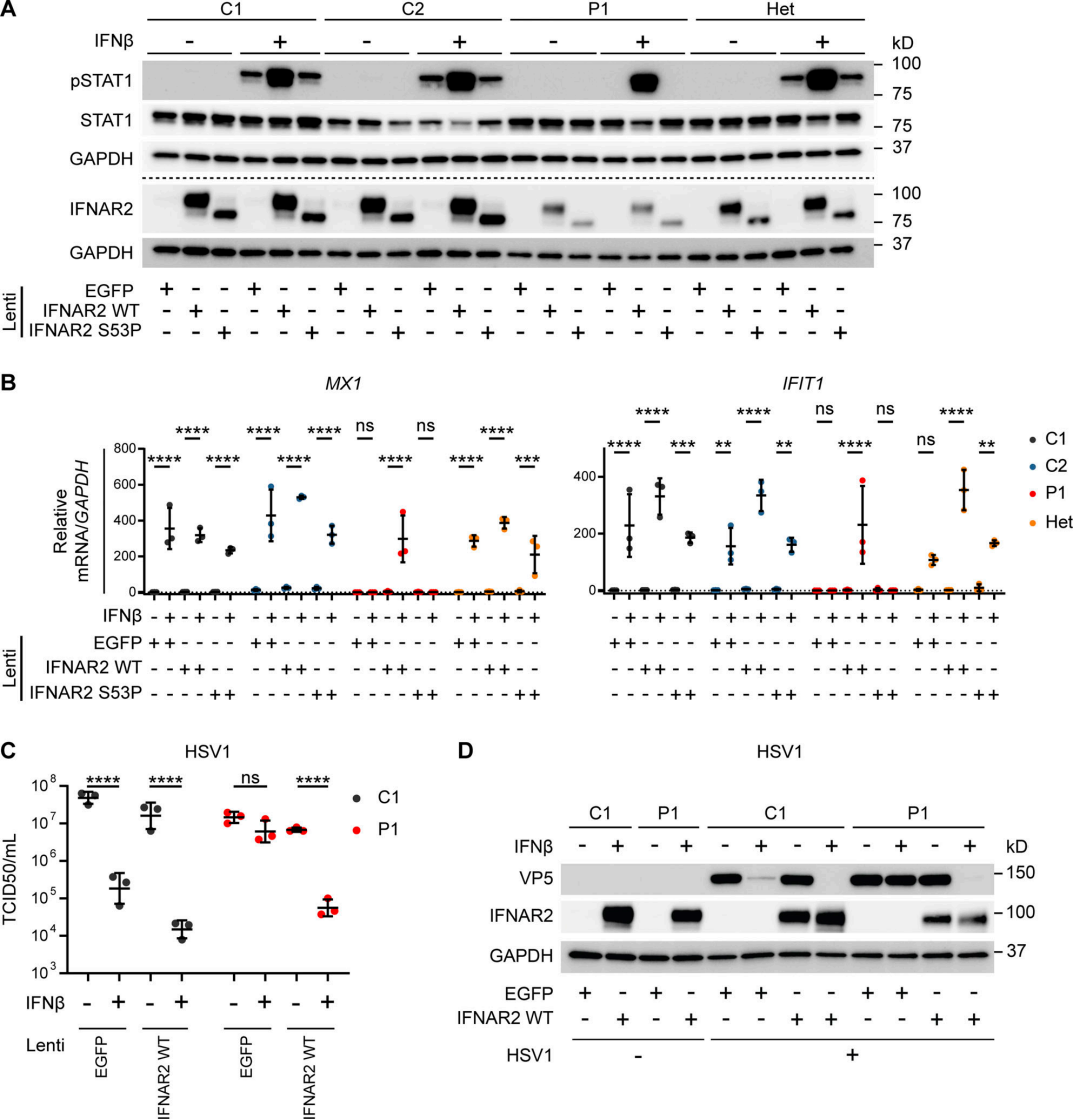
**Reconstitution of patient fibroblasts with WT *IFNAR2* restores IFNAR2 signaling, ISG induction and viral control. (A and B)** Primary dermal fibroblasts from P1, two healthy controls (C1 and C2), and the heterozygous mother of P1 (Het) were transduced with lentiviral vectors encoding *EGFP*, *IFNAR2* WT, or *IFNAR2* p.Ser53Pro. 2 d after transduction, cells were pretreated with IFNβ 100 IU/ml for either (A) 30 min before being lysed and harvested for whole cell lysates for immunoblotting or (B) 6 h before being lysed and harvested for total RNA for RT-qPCR of *MX1* and *IFIT1* relative to *GAPDH* (mean ± SD of *n* = 3 independent repeats; **, P < 0.01; ***, P < 0.001; ****, P < 0.0001; ns, non-significant; two-way ANOVA with Tukey’s test for multiple comparisons). For A, one representative immunoblot of three independent experiments is shown. **(C and D)** SV40-immortalized dermal fibroblasts from P1 and a healthy control were transduced with lentiviral vectors encoding *EGFP* or *IFNAR2* WT. 2 d after transduction, cells were pretreated with IFNβ 100 IU/ml for 24 h before infection with HSV1 (KOS strain) at an MOI of 1. At 24 h p.i., supernatants were harvested and titrated for (C) TCID50 (geometric mean ± SD of *n* = 3 independent replicates; ****, P < 0.0001; ns, non-significant; two-way ANOVA with Tukey’s test for multiple comparisons) and the cells were lysed for Western blotting for the HSV1 protein VP5, IFNAR2, and GAPDH as loading control (D). Source data are available for this figure: SourceData F5.

To demonstrate conclusively that the observed cellular phenotype was attributable to this variant, we complemented dermal fibroblasts from P1 or a heterozygous parent with WT or p.Ser53Pro *IFNAR2* by lentiviral transduction. Complementation with WT *IFNAR2* rescued the previous defect of IFNAR2 protein expression and STAT1 phosphorylation in response to IFNβ stimulation in P1 cells but had no effect on the response of heterozygous cells (Fig. 5 A). Reconstitution of the patient dermal fibroblasts with WT *IFNAR2* restored ISG expression at the mRNA level (Fig. 5 B) and reinstated the ability of IFN-I to induce an antiviral state to HSV1 (Fig. 5, C and D). The overexpression of p.Ser53Pro *IFNAR2* in healthy control cells did not appear to negatively impact tyrosine phosphorylation of STAT1 (Fig. 5 A) or ISG mRNA expression (Fig. 5 B), further suggesting that the heterozygous expression of p.Ser53Pro does not have a dominant negative impact on IFN-I signaling or antiviral response.

We report a novel cause of AR *IFNAR2* deficiency due to a missense p.Ser53Pro variant, occurring at an appreciable frequency in Greenland and the Nunavik Inuit population, and also present in the Alaska Native population, thus representing an important finding with potential implications for public health. The clinical phenotype highlights several emergent phenotypic characteristics of AR *IFNAR2* deficiency, namely: (i) susceptibility to life-threatening complications of LAVs, including VZV; (ii) virus-induced hyperinflammation, sometimes reaching clinical thresholds for the diagnosis of HLH; (iii) susceptibility to serious complications of naturally acquired viral infection (including SARS-CoV-2, HSV1, and IAV); and (iv) incomplete clinical penetrance or variable expression of the viral susceptibility phenotype.

Initial reports of patients with AR *IFNAR1* or *IFNAR2* deficiency were notable for a lack of apparent susceptibility to naturally acquired viral disease (Bastard et al., 2021b; Duncan et al., 2015; Gothe et al., 2020; Hernandez et al., 2019; Passarelli et al., 2020). More recent findings in children and adults with AR *IFNAR1* deficiency suggest this phenotype is not universal, with reports of heightened susceptibility to life-threatening COVID-19 (Khanmohammadi et al., 2021; Zhang et al., 2020) and HSV1 encephalitis (Bastard et al., 2021a). Our experience of fatal COVID-19 in P2 and recurrent severe COVID-19 in P4 reinforces findings in adults and adolescents with AR deficiency of *IFNAR1* or IFN-I autoantibodies (Bastard et al., 2020; Khanmohammadi et al., 2021; Zhang et al., 2020), and associations between severe COVID-19 and variation at the *IFNAR2* locus (Initiative, 2021; Pairo-Castineira et al., 2021). Collectively, these findings are consistent with a nonredundant role for IFN-I signaling in the control of this recently emergent pandemic virus. Our findings also extend this concept to children, who are otherwise generally protected against severe COVID-19 (Brodin, 2021), consistent with a recent report of fatal COVID-19 in a child with AR *IFNAR1* deficiency (Abolhassani et al., 2022). Interestingly, our findings also indicate that the penetrance of severe COVID-19 is incomplete in AR *IFNAR2* deficiency since P3 experienced self-limiting SARS-CoV-2 infection not requiring hospitalization. Whilst it remains to be determined where anatomically IFN-I exerts its protective effects, recent data suggest the airway may be a relevant site based on (i) studies indicating that children have robust and primed airway IFN responses to SARS-CoV-2 (Loske et al., 2021; Yoshida et al., 2022) while in adults, a more robust nasal IFN response is associated with reduced disease severity (Ziegler et al., 2021), complemented by (ii) data showing the induction of an endogenous innate IFN response in infected human nasal cells (Cheemarla et al., 2021; Hatton et al., 2021; Lopez et al., 2021).

Consistent with the emerging theme of vulnerability to certain naturally acquired pathogens in IFNAR-deficient individuals, at the time of presentation to tertiary care, P5 was noted to have extremely high levels of HSV1 viraemia, strongly suggesting defective control of this virus—although the clinical impact of HSV1 viraemia in the context of simultaneous dissemination of vaccine-strain MMR, with concomitant ARDS and HLH, is difficult to estimate. Nevertheless, this observation is consistent with a reported role for IFN-I in containing systemic HSV1 dissemination in mice (Luker et al., 2003) and importantly accords with previous reports of encephalitis in patients with AR *STAT1* (Dupuis et al., 2003), AR *IFNAR1* deficiency (Bastard et al., 2021a), and multiple defects in the TLR3 signaling pathway (Andersen et al., 2015; Casrouge et al., 2006; Herman et al., 2012; Mork et al., 2015; Perez de Diego et al., 2010; Sancho-Shimizu et al., 2011; Zhang and Casanova, 2015; Zhang et al., 2007). Collectively, these data suggest that IFN-I contributes to the control of systemic and CNS dissemination of HSV1 during primary infection, as it does in the mouse (Meyts and Casanova, 2021). Nevertheless, susceptibility to HSV1 was seemingly incomplete in AR *IFNAR2* deficiency, given the serological evidence of HSV1 exposure in P1 and the self-limiting nature of primary HSV1 infection in P2. In this context, another intriguing observation was the vulnerability to influenza A pneumonitis in P3 and P4. This phenotype—previously associated with AR deficiencies of *IRF7*, *IRF9*, *STAT2*, and *TLR3* (Ciancanelli et al., 2015; Freij et al., 2020; Hernandez et al., 2018; Lim et al., 2019) and more recently described in AR *IFNAR1* deficiency (Abolhassani et al., 2022)—suggests that IFN-I and IFN-III may play nonredundant roles in airway defense against IAV, as has been reported in mouse models (Klinkhammer et al., 2018; Shepardson et al., 2018). It is entirely conceivable that the spectrum of viral susceptibility phenotypes will expand as further cases of AR *IFNAR2* deficiency are identified.

Susceptibility to LAVs is a well-established feature of monogenic disorders of IFN-I immunity (Alosaimi et al., 2019; Bastard et al., 2021b; Bravo Garcia-Morato et al., 2019; Duncan et al., 2015; Freij et al., 2020; Gothe et al., 2020; Gothe et al., 2021; Hambleton et al., 2013; Hernandez et al., 2019; Hernandez et al., 2018; Meyts and Casanova, 2021; Moens et al., 2017; Shahni et al., 2015). Consistent with this, all previously described *IFNAR2*-deficient patients (Bastard et al., 2021b; Duncan et al., 2015; Passarelli et al., 2020), who are known to have received MMR, experienced disease temporally related to it. Nevertheless, incomplete penetrance for this phenotype has been suggested in AR *IFNAR1* deficiency (Abolhassani et al., 2022; Bastard et al., 2021a; Hernandez et al., 2019). Consistent with those reports, we observed severe or fatal complications of LAV administration in P1, P2, and P5, but no evidence of disease in P3, and relatively mild albeit widespread dissemination of vaccine-strain VZV in P4, demonstrating incomplete penetrance of the LAV susceptibility phenotype. Furthermore, the clinical severity of disease accompanying LAV administration also varied from a relatively mild clinical illness in P4, albeit requiring antiviral therapy, to neurological disease in P1 and P2, and fatal end-organ damage in P5. This variation in clinical expressivity will presumably continue to emerge as further cases of *IFNAR2* deficiency are identified. In genetic analysis, we also imputed homozygosity for the p.Ser53Pro *IFNAR2* variant in three individuals among the healthy adult Greenlandic study cohort. Whilst no information on prior vaccine exposure was available for these individuals, we cannot be absolutely certain about their genotype, and this also raises the possibility of incomplete penetrance. Our studies indicate that heterozygous carriage of the p.Ser53Pro *IFNAR2* variant is clinically silent, supported by the uncomplicated course of MMR vaccination in the heterozygous sibling of P1.

It is unclear why certain parenterally administered vaccine-strain viruses but not others caused disseminated disease, despite simultaneous exposure. Vaccine-strain VZV dissemination in P4 is a novel observation in IFNAR deficiency, whereas VZV did not appear to cause disease in P5, where dissemination of MMR was the major issue. Conversely, P4 tolerated MMR. A similar scenario was previously reported for an *IFNAR1* deficient individual who did not experience overt illness following MMR but developed severe yellow fever virus–related disease upon subsequent exposure (Hernandez et al., 2019). Reasons for the apparent virulence of some but not all LAV constituents in any given individual are unknown. Nevertheless, the clear clinical implication is that even an apparently mild episode of LAV dissemination might signal an inborn error, conferring substantial future risk, therefore warranting investigation. A related interesting observation is that the live-attenuated rotavirus vaccine, where administered, was not associated with overt disease in *IFNAR2 *deficient children. In this case, there may be an immunological explanation: the oral route of rotavirus vaccine administration would ensure it encounters the mucosal IFN-III system, consistent with the notion that IFN-III might compensate to a degree for defects in IFN-I immunity (Duncan et al., 2021). These cases highlight the clinical value of molecular virological investigation to identify whether or not the disease is associated with the vaccine-strain virus, since this provides a compelling rationale for further genetic investigation and reduces diagnostic delay. From a therapeutic perspective in patients with complete IFNAR deficiency (i.e., AR *IFNAR1* or *IFNAR2* deficiencies), the apparent vulnerability to SARS-CoV-2 and IAV emphasizes the rationale for inactivated COVID-19 and IAV vaccination, whilst the inconsistent susceptibility to HSV suggests prophylactic acyclovir may be warranted, especially in HSV/VZV seronegative individuals. Related to this, the clinical experience of one LAV may not necessarily predict the risk of disease from another—reinforcing the precautionary principle to avoid all LAVs in patients with complete IFNAR deficiency.

Globally AR *IFNAR2* deficiency is rare, with only four cases reported to date (Bastard et al., 2021b; Duncan et al., 2021; Passarelli et al., 2020). Although this novel p.Ser53Pro *IFNAR2* variant was absent from population databases, it was found in unrelated kindreds from Nunavik, Alaska, and Greenland and occurred at an appreciable frequency in association with Inuit ancestry in the analysis of cohorts in Greenland and Nunavik. This is probably due to a genetic drift, which has a particularly strong effect in geographically isolated populations where it causes some variants present in the founders to disappear from the population, and other variants, like this *IFNAR2* variant, to drift to a higher frequency (Pedersen et al., 2017). There is a general consensus that the present-day people of the circumpolar North (e.g., Greenlanders, the Nunavik Inuit, and Alaska Native groups) have common ancestors who migrated to the region ∼1,000 yr ago (Raghavan et al., 2014), suggestive of a recent founder effect. Circulation of viruses such as influenza or measles did not begin until around the early 20th century when they were associated with major epidemics (Bjerregaard et al., 2004). We speculate that selection pressure is unlikely to have been exerted by these viruses over this short period, although this is clearly a question for future population genetic studies. The deployment of live-attenuated measles vaccination and later MMR has brought undoubted benefits in the control of infectious disease in the circumpolar region (Parkinson et al., 2008), and it is essential that the identification of the p.Ser53Pro *IFNAR2* variant does not undermine the confidence in LAVs. Available data from Greenland indicate that while childhood vaccination coverage is generally high (>80%), it is slightly lower for MMR, at 77% for children aged 15 mo and 64% for children aged 4 yr, but drops to 41% in certain districts (Albertsen et al., 2020). Fewer data are available for other regions. To date, epidemiological data indicate that the COVID-19 pandemic has had an uneven impact across the circumpolar region, with substantial levels of morbidity and mortality in Alaska, Northern Russia, and Sweden, and a lower impact in Northern Canada and Greenland (Petrov et al., 2021), which is likely multifactorial and may simply reflect wider pandemic dynamics or COVID-19 vaccine coverage in these countries. Population genetic studies are immediately warranted to define p.Ser53Pro *IFNAR2* variant frequency across the circumpolar region and to understand the clinical impact of homozygosity on susceptibility to LAVs, as well as to naturally occurring viral and other infectious diseases. It is possible that population screening may be indicated and genetic counselling of affected families is warranted. The association with life-threatening complications of IAV and SARS-CoV-2 infection we describe here emphasizes the importance of prioritizing the deployment of inactivated IAV and SARS-CoV-2 vaccines across the region, especially in individuals bearing p.Ser53Pro *IFNAR2* in homozygosity. Our findings also expose gaps in our knowledge of the prevalence of potentially deleterious variants associated with primary immunodeficiency in human populations that are not well represented in databases such as gnomAD.

In contrast to previously reported pathogenic nonsense *IFNAR2* variants, the p.Ser53Pro missense variant destabilizes full-length IFNAR2 protein and results in its failure to traffic to the cell surface, associated with secondarily impaired N-glycosylation. Proline introductions are reported to cause misfolding of transmembrane proteins (Senes et al., 2004), and the reduced expression level in patient cells was consistent with instability and consequent degradation of the mutant p.Ser53Pro IFNAR2 protein. In this respect, the mechanism appears to differ from missense variants in *IFNGR2* that result in a net gain of N-glycosylation (Moncada-Velez et al., 2013). Heterozygous expression of p.Ser53Pro had minimal impact on IFNAR responses or antiviral resistance in vitro (albeit conferring a minor reduction in IFNAR2 protein expression), consistent with the apparent absence of a clinical phenotype in heterozygous carriers. The data do not exclude a more subtle effect of heterozygous carriage on IFNAR2 signaling that might be seen at a population level.

Another important question concerns the pathomechanism of hyperinflammation, an emerging phenotype in monogenic disorders of IFN-I immunity (reviewed in Duncan et al., 2021). Regrettably, no patient material was available to explore the inflammatory disease phenotype of P2 or P4. Data from mouse models indicate that IFN-I suppresses various proinflammatory cascades, including IL1β (Guarda et al., 2011; Reboldi et al., 2014) and IL17 signaling (Guo et al., 2008; Marie et al., 2021). It is also tempting to draw parallels with the model in severe/critical COVID-19, in which an inadequate early IFN-I response leads to uncontrolled late-stage activation of proinflammatory NF-κB pathways, including IL6 (Lee and Shin, 2020). The clinical response of P1 and P2 to corticosteroid and the natural history of COVID-19 in P2, P5, and other IFNAR-deficient individuals lend some support to this analogy.

In summary, we identify a novel missense variant responsible for AR *IFNAR2* deficiency in association with Inuit ancestry and displaying high mortality in the patients investigated. These cases broaden the clinical phenotype of AR *IFNAR2* deficiency, highlighting the need to thoroughly investigate for underlying genetic lesions of IFN-I immunity in patients presenting with a severe or an unusual disease in association with LAVs or naturally occurring viral infection. Since this variant occurs at a relatively high frequency, we recommend immediate population genetic studies to accurately define (i) variant frequency in other related populations in this region and (ii) the relationship to viral susceptibility, both in terms of rare complications of LAVs and susceptibility to naturally encountered viruses, including SARS-CoV-2, IAV, and HSV1.

## Materials and methods

### Clinical case summaries

#### P1

A 22-mo-old male infant from Greenland was transferred to tertiary care due to suspected meningoencephalitis. He had developed an acute febrile illness ∼10–14 d after receipt of the first dose of MMR. He was admitted to the local hospital and initially managed for Kawasaki-like illness with IVIG. Owing to a deterioration, consisting of seizure, encephalopathy, and vomiting, a lumbar puncture (LP) was performed which showed CSF pleocytosis, elevated protein, and low glucose. Treatment with ceftriaxone, acyclovir, and dexamethasone was introduced, alongside empirical quadruple tuberculosis therapy, and he was transferred to tertiary care. Extensive investigation of CSF revealed no evidence of bacterial, fungal, or viral infection, although EBV DNA was detected at a low level in both CSF (1,100 copies/ml) and subsequently in blood (2,750 copies/ml). EBV IgG was detected (alongside VZV, HSV1, and CMV IgG) 6 mo previously, suggesting EBV reactivation. Levels of CXCL13 were elevated in CSF (>500 ng/liter [normal <5 ng/liter]), reflecting meningeal inflammation. Neuroimaging with magnetic resonance imaging (MRI) was unremarkable, and PET–CT studies showed no other focus on infection. A term infant, he was developing normally prior to this illness, and has two elder half-sisters. There was no significant family history other than nephrotic syndrome (in the mother and one half-sister) and atopy. The receipt of all other routine childhood immunizations (including Bacille Calmette-Guérin) was uncomplicated. He had a past history of eczema, asthma, abdominal pain, and several episodes of acute otitis media aged 6–12 mo. At 15 mo of age he was investigated for possible tuberculosis or immunodeficiency due to right-sided lung infiltrates and lymphadenopathy. Immunological workup at this time demonstrated mild eosinophilia; normal T, B, and NK cell populations; and antibody (IgA, IgG, and IgM) levels, along with normal T cell proliferation to pokeweed mitogen and anti-CD3/CD28 beads. IgE was mildly raised (498 IU/liter, *N* < 60), consistent with atopy. Microbiological investigations were sterile, although EBV and CMV DNA were detected at low levels in both BAL and blood (alongside detectable IgG, arguing against primary infection). WGS revealed a homozygous variant in *IFNAR2* (c.157T>C, p.Ser53Pro) absent in the gnomAD and 1000 Genomes databases. The mother, father, and one half-sister were heterozygous for the *IFNAR2* variant (the other half-sister was not tested). The heterozygous sister received childhood MMR without incident. Additionally, a likely pathogenic variant in the sucrase–isomaltase gene (*SI*, NM_001041.4, c.273_274del, p.Gly92Leufs*8), a founder variant associated with Inuit ancestry (Marcadier et al., 2015), was identified in the homozygous state. This is associated with congenital sucrase–isomaltase deficiency (Marcadier et al., 2015), which has no reported immune features. P1 also bore another variant in the carnitine palmitoyltransferase 1A gene (NM_001876.4, *CPT1A* c.1436C>T, p.Pro479Leu) in the heterozygous state. This variant, which similarly has no association with immunodeficiency, is fixed (i.e., MAF 1.0) in the Inuit (Pedersen et al., 2017; Senftleber et al., 2020). All rare variants called (MAF < 0.01) in immunodeficiency genes are listed in Fig. S1 E.

#### P2

A 12-mo-old male infant from Nunavik was transferred to tertiary care with a history of meningoencephalitis. He developed a febrile illness approximately 3–4 d following receipt of MMRV. Initially, this was treated as otitis media without improvement. He was admitted to the hospital and treated with ceftriaxone and vancomycin for possible meningitis (traumatic LP), but due to persistent fever and failure to respond, he was transferred to tertiary care. Upon arrival, an MRI of the brain demonstrated leptomeningeal enhancement. CSF culture was sterile and an extended viral PCR panel was negative (including testing for MMR viruses). Despite the completion of antibiotics for bacterial meningitis, there was continued deterioration with persistent fever and the onset of seizures, presumed secondary to vasogenic oedema apparent on repeat neuroimaging. Levetiracetam was started for seizures, and there was an apparent clinical response to empirical therapy for tuberculous meningitis with adjunctive dexamethasone 0.6 mg/kg daily. Cultures of BAL and CSF were negative, although TB therapy was continued. Following initiation of steroid therapy, EBV viremia became detectable (maximum titer 16,596 copies/ml associated with detectable VCA IgG, thus presumed reactivation) but was not considered the primary cause of disease. Blood tests demonstrated elevated inflammatory markers and abnormal liver enzymes which did not reach the threshold for HLH diagnosis. Repeat MRI after 9 d demonstrated signs of improvement in both leptomeningeal enhancement and vasogenic edema. The patient was discharged home on antiepileptic therapy and a weaning course of steroids. Due to ongoing EEG abnormalities, vigabatrin was added; however a deterioration in seizure control after weaning of dexamethasone prompted an additional course of steroid (prednisolone 60 mg for 2 wk followed by a gradual reduction of 10 mg per week). He was subsequently readmitted with fever and increased seizure frequency and treated for possible aspiration pneumonitis with antibiotics. MRI showed changes apparently consistent with vigabatrin toxicity, and alternative antiepileptics were added (clobazam, topiramate, and valproic acid). He was subsequently weaned off vigabatrin. His neurological development progressed albeit delayed relative to norms for his age and he continued to experience frequent (daily) seizures. Approximately 19 mo after the index illness, aged 32 mo, he developed SARS-CoV-2 infection, testing positive on PCR analysis of a nasopharyngeal swab. Although he was hospitalized and actively managed, his condition rapidly deteriorated due to respiratory failure and he sadly passed away. Postmortem analysis was awaited at the time of writing. A term infant of second-cousin parents, he was developing normally prior to these illnesses and had three elder and one younger sibling. Receipt of all other routine childhood immunizations (including Bacille Calmette-Guérin and two doses of RotaTeq) was uncomplicated. He had a past history of multifocal bacterial pneumonia and acute otitis media aged 8 mo. He also experienced uncomplicated hand, foot, and mouth disease associated with herpangina aged 10 mo and HSV1 stomatitis requiring acyclovir treatment (first dose i.v. then oral). Immunological testing originally identified mild B cell lymphopenia during steroid therapy, which normalized upon further follow-up, and extended phenotyping identified normal expression of T and B cell memory markers and T cell proliferation to PHA and ConA, accompanied by normal IgG, IgA, and IgM levels and normal vaccine responses. Targeted WES (Prevention Genetics Lab) revealed a homozygous variant in *IFNAR2* (c.157T>C, p.Ser53Pro, rs1987287426) absent in the gnomAD and 1000 Genomes databases, and also the homozygous variant in *CPT1A* (c.1436C>T, p.Pro479Leu) identified in the homozygous state in his mother (and identified in P1). Family segregation studies demonstrated heterozygous carriage of *IFNAR2* p.Ser53Pro in both parents and his older brother, and homozygous carriage in his older sister, P3 (Fig. 1 B).

#### P3

A 7-yr-old female and the older sister of P2, P3 has a past medical history of mild intermittent asthma. Aged 5 yr, she presented to tertiary care with respiratory failure due to IAV infection complicated by bilateral pneumonitis and ARDS (Fig. S1 A). Previously healthy and in receipt of recommended vaccines, there was no report of rash or other complications associated with LAVs (i.e., MMR-V). She presented with fever and progressive difficulty in breathing for a duration of 4 d. She was admitted to the local hospital where she required high-flow oxygen via nasal cannula (15 liter/min). PCR testing detected IAV. CXR showed bilateral opacities compatible with pneumonia/ARDS (Fig. S1). Treatment with oseltamivir, prednisolone, nebulized bronchodilators, intravenous ceftriaxone, and oral azithromycin was administered. Due to a deterioration in the respiratory function, the patient was transferred to the tertiary pediatric intensive care unit (ICU) after 48 h, requiring intubation and mechanical ventilation for 6 d for severe respiratory failure. The illness was complicated by a small pneumomediastinum, which resolved spontaneously, and refractory vasoactive shock requiring epinephrine and norepinephrine infusions, intravenous albumin and hydrocortisone (septic shock stress dose). Blood and urine cultures were sterile. The patient stabilized quickly with this management and was successfully weaned from inotropic support within 24 h, eventually making a full recovery. Past medical history was also notable for SARS-CoV-2 infection in January 2022, which was self-limiting and did not require hospitalization. The patient remains well and has had no other significant viral or other infectious diseases reported.

#### P4

A 14-mo-old Alaska Native female developed acute hypoxemic respiratory failure and was transferred from an outside hospital to the pediatric ICU for BiPAP support in the setting of a progressive neuromuscular disorder of unknown etiology and a vesicular rash. The child was a term infant and was developing normally until ∼8 mo old when she began to exhibit abnormal movements with developmental regression and failure to thrive on nasogastric feeds. At 13 mo old, she showed worsening hypotonia, gross motor regression, weight loss, and jerking movements. She was hospitalized at 13 mo for respiratory distress and diagnosed with influenza A and associated pneumonia requiring supplemental oxygen and intravenous antibiotics. She received two courses of oseltamivir. On day 6 of this hospitalization, she received the MMR, varicella, Haemophilus influenza B, pneumococcal 13-valent vaccine, inactivated influenza, and hepatitis A vaccine per standard pediatric schedule catch up. 1 wk later, she developed worsening respiratory distress and increasing high-flow nasal cannula oxygen requirements in the ICU. 18 d following immunization, β-lactam antibiotics were discontinued due to concern for a drug reaction. Her hospitalization was complicated by EBV infection (IgM positive, IgG negative) and at 23 d after immunization by a new papular rash. At 30 d after immunization, she was transferred to a tertiary referral hospital into the ICU, and upon admission, a vesicular rash with variable crusting was appreciated on the face, chest, abdomen, and all extremities. Skin lesion viral PCR was positive for VZV and negative for HSV1/2. Blood PCR was similarly positive for VZV with 880 copies/ml (log 2.9). Rapid CSF PCR was positive for HHV6, negative for CMV, enterovirus, HSV1/2, parechovirus, and VZV. Blood PCR was below the level of detection for HHV6. She subsequently underwent BAL showing positive PCR for VZV, negative for a panel of respiratory viruses and negative for CMV and HSV1/2. The VZV was sent to the Centers for Disease Control and Prevention for typing and was confirmed to be Oka vaccine strain. She was treated with intravenous acyclovir and was able to clear VZV with three negative blood PCRs prior to drug discontinuation. An immunologic work-up was initiated given her vaccine-strain VZV testing. Upon review, the child had normal newborn screening T cell receptor excision circle testing. During her hospitalization, she demonstrated normal immunoglobulin levels (IgG, IgM, IgA), normal lymphocyte subsets (CD3^+^, CD19^+^, CD56^+^, CD4:CD8 ratio, 69% CD4^+^CD45RA^+^), and adequate lymphocyte proliferation response to mitogens (phytohemagglutinin). She had mildly decreased proliferative response to anti-CD3 testing and limited proliferation in response to tetanus. CD107a degranulation testing was normal indicating no inherent defect in her cytolytic response. Commercial WES revealed a homozygous variant in *IFNAR2* (c.157T>C, p.Ser53Pro) absent in gnomAD and 1000 Genomes databases. Parents were not available for testing. In winter 2020, P4 contracted SARS-CoV-2 virus and received remdesivir and dexamethasone for 10 d and was discharged home on room air. 2 mo later, she developed recurrent hypoxia with bilateral radiological infiltrates on CXR and again tested positive for SARS-CoV-2 on a nasopharyngeal swab. She received a second course of remdesivir and dexamethasone for a further 10 d. Echocardiographic examination was normal at that time. She was discharged after 2 wk with an airway clearance plan. Since then she experienced a further admission with respiratory difficulties (testing for SARS-CoV-2 negative) and required parenteral antibiotics for urosepsis. Owing to progressive neurological decline and frequency of hospitalization, after extensive discussion between the family and treating clinical team, P4 was subsequently transferred for hospice care, succumbing to her illness aged 3 yr.

#### P5

A 13-mo-old Alaska Native male infant with no significant medical history developed ARDS and was transferred from an outside hospital to the pediatric ICU. 11 d prior to admission, the child had presented for bilateral conjunctivitis and received ophthalmic erythromycin ointment and subsequently received M-M-R-II, VZV, haemophilus influenza B (PRP-OMP), pneumococcal conjugate 13-valent vaccine, inactivated influenza, and hepatitis A vaccine per standard pediatric schedule. 2 d following immunization, he presented again for ear pain and started amoxicillin for an acute otitis media. The next day, he was seen in the emergency department for fever (102°F), cough, and worsening conjunctivitis and was switched to broader coverage antibiotics. 4 d after vaccination the child was admitted for fever, respiratory distress, stridor, oliguria, somnolence, and cervical lymphadenopathy. Treatment included intravenous fluids and initially dexamethasone and racemic epinephrine for stridor. Influenza A/B testing was negative. LP was reassuring (WBC 2 cells, RBC, protein, glucose normal) and cultures from blood and CSF were ultimately negative. The child developed inspiratory and expiratory stridor with erythematous exudative pharynx with ulcerations. The left tympanic membrane was bulging with pus. Under sedation and intubation, severe inflammation was observed in the nasopharynx and oropharynx with exudative tonsillitis and edematous epiglottitis with “white polka dots” described. The child was treated with meropenem and vancomycin. A neck CT scan revealed diffuse soft tissue thickening of the posterior nasopharynx, mildly thickened uvula, and diffuse soft tissue thickening of the oropharynx and hypopharynx with phlegmon and no distinct abscess. A respiratory viral panel was positive for RSV and adenovirus. The child had progressive worsening of respiratory failure on FiO_2_ 80–100% and inhaled nitric oxide without improvement. He was transferred to a tertiary referral center. The child’s past medical history included a term birth complicated by meconium. He had a history of only episodes of acute otitis media and otherwise normal growth and development. P5 was the youngest of six siblings, the rest of whom were reported to be healthy. Both the mother and father reported a history of recurrent otitis media, with his father reporting hearing loss secondary to recurrent left-sided otitis media. He had received all age-appropriate vaccines including the rotavirus (RotaTeq). Upon transfer 12 d after vaccination, the critically ill child required venovenous extracorporeal membrane oxygenation (VV-ECMO), renal replacement, and plasmapheresis. Viral studies indicated serum adenovirus (1,995 copies/ml, log 3.3), and thus cidofovir was started. He also received anakinra for possible HLH. On his third day of admission, one papular and one papulovesicular skin lesion were identified, testing positive by PCR for HSV-1, and acyclovir was started. A bone marrow biopsy revealed hemophagocytic histiocytes, and moderate dose dexamethasone was commenced to treat HLH. He was switched to veno-arterial (VA)-ECMO. HSV-1 PCR from blood revealed a high viral load at 2 million copies/ml (log 6.3). BAL was negative for bacteria and fungi by culture and PCR testing. Pneumocystis testing and smears for acid/alcohol fast bacilli were negative. PCR testing from the BAL was positive for adenovirus, RSV, CMV, EBV, HHV6, and HSV-1. Notably, he was negative for VZV. Cytology revealed rare multinucleated giant cells. CSF testing was strongly positive for HSV1 (Log 5.0–5.1). Serology was not available to assay prior exposure to herpesviruses. Needle biopsy for the left cervical lymph node showed rare scattered EBV-positive staining cells consistent with an earlier infection. He was maintained on meropenem, linezolid, acyclovir, cidofovir, and given methylprednisolone (10 mg/kg) for ongoing concern for HLH. RT-PCR testing at the Centers for Disease Control and Prevention was positive for vaccine strain MeV from the nasopharyngeal swab, serum, and urine; for vaccine strain mumps from NP swab and urine; vaccine strain rubella from NP swab, serum, and urine. Given the vaccine strain information, IVIG was given, along with high dose vitamin A and enteral ribavirin. The child was compassionately extubated the next day due to refractory shock and ARDS in the setting of a presumed primary immunodeficiency. Normal immunoglobulins and lymphocyte subset percentages were found initially while on ECMO with normal Hib antibody titers and normal NK cell function testing, accompanied by normal perforin, granzyme B, and X-linked inhibitor of apoptosis protein (XIAP) protein expression by flow cytometry. Post-mortem genetic testing (Immunoplex Gene panel) revealed a homozygous variant in *IFNAR2* (c.157T>C, p.Ser53Pro) absent in gnomAD and 1000 Genomes databases and a hemizygous variant of uncertain significance in the *MSN* gene (c.1095G>A). Parents and siblings have not yet undergone genetic testing.

### Ethics statement and clinical genetic studies

Laboratory studies on the samples collected from the patients and family members for the purposes of functional validation were conducted as a part of clinical care. Parental consent was obtained for all children in the report. Approval for functional immunology analysis of patient cells was given by the Danish National Committee on Health Research Ethics (project ref: 1-10-72-275-15) and all protocols followed the principles stated in the Declaration of Helsinki. Approval to report the Alaskan cases was provided by the Yukon-Kuskokwim Health Corporation’s Executive Board of Directors, the Alaska Native Tribal Health Consortium’s Health Research Review Committee, and Southcentral Foundation’s Executive Committee. WGS data were obtained from participants in the Greenlandic Population Study 2015–22. The study was approved by the Commission for Scientific Research in Greenland (project 2011-13, ref. no. 2011-056978, project 2013-13, Ref: 2013-090702, and KVUG 2017-10) and written informed consent was obtained from all participants. In the genomic study from Nunavik, written informed consent was obtained from all participants, and the study was approved by the McGill University Ethics Committee (ND 04.101) and the Nunavik Nutrition and Health Ethics Committee.

### Next-generation sequencing analysis

Whole exome or panel sequencing and reporting were undertaken by commercial providers (P2–5), and WGS (P1) was undertaken according to the following method: DNA from whole blood samples was isolated using the liquid handling automated station (Tecan). Genomic DNA (500 ng) was subjected to WGS using Nextera DNA Flex library preparation kit and sequenced on a Novaseq 6000 (Illumina) to an average coverage depth of minimum 30-fold, and minimum 98% of the genome was sequenced to a coverage depth of 10-fold (reference genome hg19). Data were processed through GATK v4.1.0 in accordance with the GATK best practice developed by the Broad Institute. Structural variants were called by Manta (Chen et al., 2016) in combination with Delly2 (Rausch et al., 2012), Lumpy (Layer et al., 2014), and CNVnator (Abyzov et al., 2011), which added manual inspection of the sequences. The variants were classified in accordance with the American College of Medical Genetics and Genomics and the Association for Molecular Pathology guidelines (Richards et al., 2015). For sharing of the patient WGSs, a data transfer agreement between relevant parties/institutions will be created in case of the need for data or material transfer for scientific or medical purposes.

### Variant population genetics analysis

The frequency of the *IFNAR2* (c.157T>C, p.Ser53Pro) variant was estimated using unpublished WGS data from individuals from Greenland and published WES data from individuals from Nunavik (Zhou et al., 2019). The variant was also imputed from a larger dataset of genome-wide SNP chip data (Illumina MEGA chip) from Greenland. Imputation was carried out as follows: data were pre-phased using SHAPEIT2 (Delaneau et al., 2011) using information from all trios and duos in the data. We used both the high depth whole genomes from 448 Greenlandic individuals and seven European and East Asian populations (1000 Genomes Project Phase 3; Genomes Project et al., 2015) as reference panels. We phased and imputed the SNP chip genotyped individuals using shapeit2 and IMPUTE2 (Howie et al., 2011), respectively. The *IFNAR2* variant had an info score of 0.987 in the imputation. This resulted in a final data set of 4,630 phased genomes. The Greenlandic individuals were merged with individuals from the superpopulations of East Asia, Europe, and America from the 1000 Genomes Project. Sites with MAF <5% across all individuals were removed. Remaining sites were LD-pruned (r2-threshold = 0.8) and principal components were computed, all using PLINK (v1.90b6; Chang et al., 2015).

We calculated the local ancestry using an Inuit reference based on unadmixed Greenlanders and unrelated individuals from 1000 Genomes populations (Toscani in Italy [TSI], Utah residents with Northern and Western European ancestry [CEU], British in England and Scotland [GBR], and Iberian populations in Spain [IBS]) as the European reference. The unadmixed Greenlanders were selected as individuals having >99% Inuit-ancestry based on ADMIXTURE (Alexander et al., 2009). Local ancestry was inferred using RFMix (v2.03-r0; Maples et al., 2013), where we used the phased data of sites with MAF > 5% across all individuals. We used the local ancestry for visualization and ancestry-specific allele frequency calculation. 11 individuals with East Asian ancestry were excluded from the local ancestry analysis.

### Cells and cytokines

Primary human dermal fibroblast lines were generated from skin biopsies obtained from P1 and a heterozygous parental carrier of the *IFNAR2* p.Ser53Pro variant. Healthy control human dermal fibroblast lines were obtained from PromoCell or existing stocks. Where indicated, fibroblast lines were immortalized by co-transfection of the hyperactive sleeping beauty transposon (SB100X; Sharma et al., 2013) and simian virus 40 (SV40) plasmids (Brinck Andersen et al., 2021) using Lipofectamine 3000 Transfection Reagent (L3000001; Invitrogen) according to the manufacturer’s instructions. The IFNAR2-deficient patient dermal fibroblast line was obtained under separate ethical consent, as described previously (Duncan et al., 2015). EBV-transformed B cells were generated from P2 and healthy controls, as previously described (Duncan et al., 2019). Permissive cell lines (HEK293T/FT cells, Vero cells) were obtained from Thermo Fisher Scientific (R70007; Invitrogen) and the American Tissue Culture Collection (accession CLL-81TM). Vero E6 cells were a kind gift of Richard Randall (University of St Andrews, St Andrews, UK). Adherent cells were maintained in DMEM supplemented by 10% (vol/vol) FCS, 1% (vol/vol) penicillin/streptomycin (100 U/ml and 100 μg/ml respectively; Sigma-Aldrich, P0781), and 1% L-glutamine 2 mM; Sigma-Aldrich, G7513), termed DMEM-10. PBMCs were isolated from peripheral blood samples taken from P1 in lithium heparin or P2 in EDTA-containing vacutainer tubes (BD Biosciences [BD]) and were isolated using Ficoll (Lymphoprep; STEMCELL Technologies) according to manufacturer’s instructions. PBMC and EBV-transformed B cells were maintained in RPMI-1640 medium (R0883; Sigma-Aldrich) supplemented with 10% (vol/vol) FCS (10270-106; Gibco), 1% (vol/vol) penicillin/streptomycin (100 U/ml and 100 μg/ml respectively; P0781; Sigma-Aldrich), and 1% (vol/vol) L-glutamine (2 mM; G7513; Sigma-Aldrich), termed RPMI-10. All cells were cultured at 37°C in 5% CO_2_. Human recombinant IFNα2b (Intron-A; Schering-Plough) and IFNγ (Immunikin; Boehringer Ingelheim) were used at 1,000 IU/ml. Human recombinant IFNβ (PeproTech) was used at 100 IU/ml.

### Viruses

Herpes simplex virus 1 (HSV1, 17+ strain) was kindly provided by William James (University of Oxford, Oxford, UK). HSV1 KOS strain was a kind gift from Peter O’Hare (Imperial College London, London, UK). Encephalomyocarditis virus (EMCV) and MuV (Enders strain) were kindly provided by Richard Randall (University of St Andrews, St Andrews, UK). MeV (Edmonston strain) was obtained from the American Type Culture Collection (VR-24). VZV (rOka strain) was obtained from the American Type Culture Collection (VR1832). Viruses were propagated on permissive cells: Vero cells (HSV1 KOS and 17+, MeV), vero E6 cells (MuV) or MeWo-cells (VZV), and the titer was determined by plaque assay according to standard methods (HSV1 17+, MuV, EMCV) or as described previously for VZV (Ogunjimi et al., 2017) and HSV1 KOS (Bodda et al., 2020). For MeV, cells were cultured in DMEM supplemented with 330 µg/ml of pooled IgG (Gammanorm; Octapharma), whereas for other viruses, cells were cultured in 1.2% colloidal microcrystalline cellulose (Sigma-Aldrich) as the semisolid overlay (Bodda et al., 2020; Gothe et al., 2020). Cells were fixed with 4% formaldehyde and stained with 0.5% crystal violet for plaque visualization.

Cells were infected for experimental purposes by exposure to a known viral inoculum at a range of MOI in DMEM. At an appropriate time after infection (1 h for HSV1, 2 h for Mev/Muv, 4 h for VZV), the inoculum was removed and replaced with fresh medium (DMEM-10). Cells were incubated for varying times prior to lysis or imaging, as described in the sections below, with the removal of the supernatant for virus quantification of infectious virus content by either plaque assay or end-point dilution assay on Vero cells. Briefly, in the end-point dilution assay, 30,000 cells were seeded per well in 96-well plates (eight replicates) and incubated with a 10-fold dilution of virus-containing supernatant. At 72 h (HSV1 KOS) or 7 d (MeV) after infection, the wells were inspected for cytopathic effect and the 50% tissue culture infective dose (TCID50) was calculated, using the Reed-Muench method (Skouboe et al., 2018).

### Generation of *IFNAR2* expression constructs

Two approaches were taken to generate lentiviral expression constructs in the collaborating laboratories, and both are summarized below. In the first, the coding sequences of *IFNAR2* and *IFNAR2* c.157T>C were PCR-amplified from a *IFNAR2* encoding plasmid (kind gift from Rune Hartmann) using the primers 5′-GGC​CTT​TCG​ACC​TCT​AGC​GGG​ATC​CAC​CGG​TCG​CCA​CCA​TGC​TTT​TGA​GCC​AGA​ATG​C-3′ and 5′-GGT​TGA​TTA​TCG​GAA​TTC​CCT​CGA​GGC​CGC​TTC​ATC​TCA​TTA​TAT​AAC​CAT-3′ or 5′-GGC​CTT​TCG​ACC​TCT​AGC​GGG​ATC​CAC​CGG​TCG​CCA​CCA​TGC​TTT​TGA​GCC​AGA​ATG​C-3′, 5′-GGT​TGA​TTA​TCG​GAA​TTC​CCT​CGA​GGC​CGC​TTC​ATC​TCA​TTA​TAT​AAC​CAT-3′, 5′-TAA​GAT​GGA​CCG​GAA​ATT​TC-3′, and 5′-GAA​ATT​TCC​GGT​CCA​TCT​TAC​CAT​GGG​AAT​TAA​AAA​ACC​AC-3′. The PCR-fragments were inserted into BamHI/XhoI-digested pCCL/PGK-eGFP using the NEBuilder HiFi DNA Assembly Cloning Kit (New England Biolabs) according to the manufacturer’s protocol. The resulting plasmids were designated pCCL/PGK-IFNAR2 and pCCL/PGK-IFNAR2 c.157T>C, respectively. Both plasmids were verified by Sanger sequencing.

The second approach involved site-directed mutagenesis of a Gateway-compatible lentiviral vector containing the full-length open reading frame of human *IFNAR2* transcript variant 1 (NM_207585) under the control of the constitutive promoter EF1α, with a GFP-Puromycin selection marker under an RSV promoter, previously purchased from AMS Bio (Abingdon; Duncan et al., 2015). Agilent QuikChange XL kit was used to create the p.Ser53Pro mutation on *IFNAR2* lentiviral vector. Site-directed mutagenesis primers were designed by using Agilent QuikChange primer design tool: 5′-TTT​TTT​AAT​TCC​CAT​GGT​AAG​ATG​GAC​CGG​AAA​TTT​CGC​AAT​GAT​A-3′; 5′-TAT​CAT​TGC​GAA​ATT​TCC​GGT​CCA​TCT​TAC​CAT​GGG​AAT​TAA​AAA​A-3′.

Additionally, *IFNAR2* WT and p.Ser53Pro open reading frames were subcloned to CMV immediate early promoter-driven and N-terminal hemagglutinin (HA)-tagged expression vector pCR3-N-HA via pDONR207 by Gateway cloning according to manufacturer’s instructions (12535-019 and 12535-027; Thermo Fisher Scientific). Both plasmids were verified by Sanger sequencing (GATC sequencing; Eurofins).

### Lentiviral production and transduction

Two approaches were adopted as summarized below. In the first, third-generation lentiviral vectors were produced as previously described (Hollensen et al., 2017). Briefly, HEK293T cells were transfected with pMD.2G, 3 μg pRSV-Rev, 13 μg pMDLg/pRRE, and 13 μg lentiviral transfer vector for 10-cm dishes or 9.07 μg pMD.2G, 7.26 μg pRSV-Rev, 31.46 μg pMDLg/pRRE, and 31.46 μg lentiviral transfer vector for 15-cm dishes using a standard polyethylenimine transfection protocol. 5 h after transfection, the medium was changed. 2 d after transfection, viral supernatants were harvested, filtered, and titered by quantification of the number of lentiviral integrations in dermal fibroblasts as previously described (Bak et al., 2013). Dermal fibroblasts were transduced using MOI of 1. Both lentiviral supernatants and cell culture medium were supplemented with 8 μg/ml hexadimethrine bromine (Polybrene; Merck). 1 d after transduction, the medium was changed. 2 d after transduction, pCCL/PGK-EGFP–treated cells were inspected for green fluorescence as a proxy for successful transduction before the cells were harvested for experiments. Alternatively, HEK293FT cells were co-transfected with the lentiviral transfer vector, pCMV-VSV-G (gifted by David Young [Newcastle University, Newcastle, UK]) and psPAX2 at a ratio of (1:1:1; Gothe et al., 2020) using FuGENE HD reagent (Promega) according to the manufacturer’s protocol. After 48 h, Lenti-X Concentrator (Takara) was added to the filtered media according to the manufacturer’s protocol, and subsequent viral pellets were stored at −80°C. Primary fibroblasts were spinoculated at 2,000 rpm for 90 min with lentivirus in DMEM-10 (without penicillin/streptomycin) containing 8 μg/ml hexadimethrine bromine (Polybrene; Sigma-Aldrich; Duncan et al., 2019). Fibroblasts were subsequently incubated with lentivirus-containing media for up to 24 h, after which the medium was replaced with DMEM-10. After 48 h, the transduced cells were selected with 0.5 μg/ml puromycin (Sigma-Aldrich). Antibiotic-containing medium was refreshed every 72 h.

### Transient transfection

HEK293FT cells were transfected with expression vectors encoding WT or p.Ser53Pro *IFNAR2* using FuGENE HD reagent (Promega) according to the manufacturer’s instructions. As indicated, CHX (Cell Signaling Technologies) was added at the indicated concentrations 24 h after transfection, alongside DMSO vehicle control.

### Live cell viability assay

Dermal fibroblasts were treated and infected as stated in the respective experimental descriptions. At the point of endpoint analysis, the medium was removed and replaced with Live Cell Imaging Solution (Thermo Fisher Scientific) containing 2 drops per ml of propidium iodide and Hoechst. Plates were then incubated for 15 min at 37°C and 5% CO_2_ before image collection using an EVOS FL fluorescence microscope (Thermo Fisher Scientific). Image analysis was performed using a bespoke pipeline developed in CellProfiler (Broad Institute) to calculate the percentage of dead cells in each condition (Hanrath et al., 2022 *Preprint*). All experiments were performed in technical duplicates, and the average value of *n* = 4 images per well was used for analysis.

### Immunofluorescence analysis

Primary fibroblasts or transfected HEK293FT cells in 8-well chamber slides (µ-Slide 8 Well ibiTreat chamber slide; ibidi) were fixed with 4% formaldehyde for 20 min and washed with PBS. Slides were blocked with 10% donkey serum/0.3% Triton Xs-100 in PBS and then incubated with primary antibodies (sheep anti-IFNAR2, R&D Biosciences, AF7014; mouse anti-RCAS1, Cell Signaling Technology, 67856; or mouse anti-Calnexin, BD, 610524) at 4°C overnight. Subsequently, they were washed with 0.1% Tween in PBS and incubated with appropriate secondary antibodies (anti-mouse Alexa 488; anti-sheep Alexa 594; Thermo Fisher Scientific) for 1 h at room temperature, followed by nuclear staining with 0.2 μg/ml DAPI (Thermo Fisher Scientific). Slides were mounted with ProLong Glass Antifade Mountant (Thermo Fisher Scientific) and imaged at room temperature on a Leica SP8-STED Confocal Microscope (Leica Microsystems) equipped with supercontinuum white light lasers (470–670 nm), solid state 405 nm laser, and hybrid detectors. Images were acquired via oil immersion at 63× magnification with a 1.4 aperture lens using Leica LAS X software. Confocal images were deconvolved and Pearson colocalization coefficients were analyzed using the software by Huygens Professional. The type of deconvolution used by Huygens Professional is classic maximum likelihood estimation.

### Immunoblotting

Confluent monolayers of fibroblasts or 5 × 10^5^ EBV B cells were washed with PBS and lysed using radioimmunoprecipitation assay buffer (150 mM sodium chloride, 1% Triton X-100, 0.5% sodium deoxycholate, 0.1% sodium dodecyl sulphate, 50 mM Tris pH8) supplemented with protease inhibitor (Halt [Thermo Fisher Scientific] or Complete [Roche]), Benzonase nuclease [Sigma-Aldrich]), 1 mM sodium orthovanadate, 10 mM sodium fluoride, 10% (vol/vol) dithiothreitol, and 1× NuPAGE LDS sample buffer (Thermo Fisher Scientific) or alternatively 4× Laemmli sample buffer (Bio-Rad) with 5% vol/vol 1 M dithiothreitol. Sample DNA was sheared using a 29G needle (or Benzonase if not previously added) to reduce viscosity and either an equal volume of lysate was loaded, or the protein concentration was measured with the BCA protein assay (Thermo Fisher Scientific) prior to loading. Lysates were denatured at 70°C for 15 min then loaded on to 4–12% Bis-Tris gel alongside a prestained protein ladder (PageRuler Plus; Thermo Fisher Scientific) for gel electrophoresis in 1× NuPAGE 3-(N-morpholino)propanesulfonic acid SDS Running Buffer (Invitrogen). Proteins were transferred to 0.45 μM polyvinyl difluoride membranes (Millipore) at 25V using 1× NuPAGE Transfer Buffer (Invitrogen) in 20% methanol. Membranes were blocked for 60 min using either 5% bovine serum albumin in Tris-buffered saline with 0.1% Tween or 5% skim milk prior to immunostaining. Membranes were incubated overnight with primary antibodies followed by the appropriate secondary antibody. Primary antibodies were from Cell Signaling Technologies, unless specified: Sheep anti-IFNAR2 (C-terminal, AF7014; R&D Systems); rabbit anti-USP18 (D4E7); rabbit anti-RSAD2 (13996); rabbit anti-ISG15 (2743); rabbit anti-STAT1 (9172); rabbit anti-pSTAT1 (7649); mouse anti-pSTAT1 (612132; BD); rabbit anti-MXA (sc-166412; Santa Cruz Biotechnology); mouse anti-MuV NP (ab9880; Abcam); sheep anti-HSV gD (sc-21719; Santa Cruz Biotechnology); mouse anti-HSV ICP5/VP5 (ab6508; Abcam); mouse anti–α-tubulin (3873); rabbit anti-GAPDH (5174); mouse anti-GAPDH (sc-25778; Santa Cruz Biotechnology); and rabbit anti-GFP (2956). Membranes were developed with Immobilon ECL substrate (Millipore) or Clarity/Clarity Max ECL substrate (Bio-Rad), and chemiluminescent images were visualized with the LI-COR Odyssey (LI-COR) or ChemiDoc system (Bio-Rad).

### Flow cytometry analysis of IFNAR2 expression

Immunophenotyping of PBMCs from P1 and healthy controls was performed by flow cytometry using the following monoclonal mouse anti-human antibodies: IFNAR2 PE (clone REA124, REAfinity; Miltenyi Biotec 130-099-555) in a panel with CD14 FITC (BD, 555397), CD3 PerCP-Cy5.5 (BD, 624060), CD19 PE-Cy7 (BD, 341113), CD4 APC (BD, 345771), CD8 APC-Cy7 (BD, 348813), and CD45 V500 (BD, 560777). Isotype control antibodies were: IgG1 PE (REAfinity, Miltenyi) and IgG1 isotypes conjugated with FITC, PerCP-Cy5.5, PE-Cy7, APC, APC-Cy7, and V500, respectively (all from BD). In brief, PBMC samples were defrosted, washed, and resuspended in 100 μl FACSFlow buffer (BD) and antibodies were added. Cells were stained for 30 min, washed, and analyzed on a BD Fortessa flow cytometer (Becton Dickinson Biosciences) equipped with the relevant lasers and filters. Immunophenotyping of PBMCs from P2 and healthy travel controls was performed as outlined above with the following modifications: cells were rested in prewarmed RPMI-10 for 2 h prior to staining with antibodies for 50 min at room temperature in 200 μl FACS staining buffer (PBS + 2% vol/vol FCS). Antibodies used, in addition to the IFNAR2 antibody above, were: CD19 PerCP-Cy5.5 (Biolegend [BL], 302230); CD20 BV605 (BD, 740333); CD56 PE-CF594 (BD, 564849); CD14 APC (BL, 301808); CD16 BV650 (BD, 563546); CD3 BUV395 (BD, 563546); HLA-DR BV711 (BL, 307644). Cells were then washed and incubated with 7AAD (1:40 dilution; BL) prior to acquisition on a Symphony A5 flow cytometer (Beckman Coulter). Data were analyzed by FlowJo V10 (BD).

Surface expression of IFNAR2 was assessed by flow cytometry analysis of HEK293FT cells transfected with *IFNAR2* expression constructs as follows: 24 h after transfection cells were lifted by gentle scraping into 5 mM EDTA/PBS and incubated in FACS staining buffer with IFNAR2 PE (clone REA124) or isotype control IgG1 PE (REAfinity; Miltenyi Biotech) for 50 min at room temperature and then washed and incubated with 7AAD (1:40 dilution; BL) before acquisition on a Symphony A5 flow cytometer (Beckman Coulter). Data were analyzed by FlowJo V10 (BD).

### RT-PCR

RNA was purified using the NucleoSpin 96 RNA Core Kit (MACHEREY-NAGEL) according to the manufacturer’s protocol. First-strand cDNA synthesis was carried out using the Iscript gDNA Clear cDNA Synthesis Kit (Bio-Rad) according to the manufacturer’s protocol. For quantification of *IFNB1*, *IFIT1*, *IFNAR2*, *IRF7*, and *MX1* mRNA levels qPCR reactions were prepared using TaqMan Fast Advanced Master Mix (Thermo Fisher Scientific) and gene-specific primers and probes (*TBP*: Hs00427620_m1; *IFNB1*: Hs01077958_s1; *IFIT1*(ISG56): Hs03027069_s1; *IFNAR2*: Hs01022064_m1; *IRF7*: Hs01014809_g1; *MX1*: Hs00895608_m1 [Thermo Fisher Scientific]) according to the manufacturer’s protocol. For the quantification of VZV *ORF40* and *ORF63* RNA levels qPCR reactions were prepared using Brilliant III Ultra-Fast SYBR Green QPCR Master Mix (Agilent Technologies) and gene-specific primers for *GAPDH* (5′-TCTTTTGCGTCG-3′, 5′-ACCAGGCGCCCA-3′), *ORF40* (5′-ACTTGGTAACCG-3′, 5′-CGGGCTACATCA-3′), and *ORF63* (5′-GCGCCGGCATGA-3′, 5′-GACACGAGCCAA-3′) according to the manufacturer’s protocol. A CFX96 Real-Time System (Bio-Ras) was used for the quantification of RNA levels relative to the expression of *TBP* or *GAPDH* using the $2^{{-\Delta \Delta C}_{T}}$ method (Livak and Schmittgen, 2001), and the expression levels were normalized to the controls.

### Endoglycosidase treatment

Protein lysates from healthy donor fibroblasts transduced with either WT *IFNAR2* or p.Ser53Pro *IFNAR2* were treated with the O-Glycosidase and Neuraminidase Bundle kit (E0540S; New England Biolabs) according to manufacturer’s instructions. Protein lysates from HEK293FT cells transfected with either WT *IFNAR2* or p.Ser53Pro *IFNAR2* were treated with PNGase F (New England Biolabs) according to the manufacturer’s instructions. IFNAR2 expression was analyzed by immunoblotting as described above.

### In silico variant prediction and protein modeling

PhastCons is a method to determine the grade of conservation of a given nucleotide, given as a score from 0-1 (Siepel et al., 2005). MutationTaster uses values that are precomputed and offered by the University of California Santa Cruz (Schwarz et al., 2010). The Combined Annotation Dependent Depletion score is a tool for integrating conservation and deleteriousness predictions (Rentzsch et al., 2019). Additional tools for predicting the impact of variants (e.g., PolyPhen-2, SIFT, and Align-GVGD) were utilized (Adzhubei et al., 2010; Tavtigian et al., 2006; Vaser et al., 2016). A graphical depiction of the binary complex between human IFNα2 and IFNAR2 (PDB accession no. 3S9D) was created using PyMOL software version 2.0.7 (Schrodinger). The PremPS database (Chen et al., 2020) was used to calculate protein-free energy changes (ΔΔGG), and the DynaMUT database (Rodrigues et al., 2018) was used to assess changes in vibrational entropy energy (ΔΔSVib ENCoM) for the p.Ser53Pro mutant. For these predictions, PDB 1N6V IFNAR2 was used. This structure lacks 27 N-terminal residues, therefore p.Ser53Pro is denoted as S26 in this structure.

### Statistical analysis

All experiments were repeated a minimum of *n* = 3 times unless otherwise stated. The mean of technical replicates for each biological repeat was used in the data analysis. Data were analyzed using Welch’s or Student’s two sample *t* test (two groups), one-way ANOVA (same group, multiple conditions or multiple groups, same condition), or two-way ANOVA (multiple groups and conditions), with relevant post-hoc corrections for multiple comparisons, as described in the respective figure legends. TCID50 values were log-transformed prior to analysis. Analysis was done with Prism version 9.2 (GraphPad Software). All tests were two-tailed and an adjusted *α* < 0.05 was considered statistically significant.

### Online supplemental material

Included in the supplemental material are three supplementary figures showing chest radiography from P3, describing variant modeling and showing all rare variants identified in WGS data from P1 (Fig. S1), flow cytometry gating strategies (Fig. S2), STAT1 immunoblotting from P2, and reconstitution of IFNAR2-deficient fibroblasts alongside antiviral responses of these cells (Fig. S3).

## Supplementary Material

SourceData F1 contains original blots for Fig. 1.

SourceData F2 contains original blots for Fig. 2.

SourceData F3 contains original blots for Fig. 3.

SourceData F4 contains original blots for Fig. 4.

SourceData F5 contains original blots for Fig. 5.

SourceData FS3 contains original blots for Fig. S3.
