# Supplementary figures and images for: Life-threatening viral disease in a novel form of autosomal recessive IFNAR2 deficiency in the Arctic
Source: J Exp Med. 2022 Apr 20;219(6):e20212427. doi: 10.1084/jem.20212427 (PMC9026249; doi:10.1084/jem.20212427)

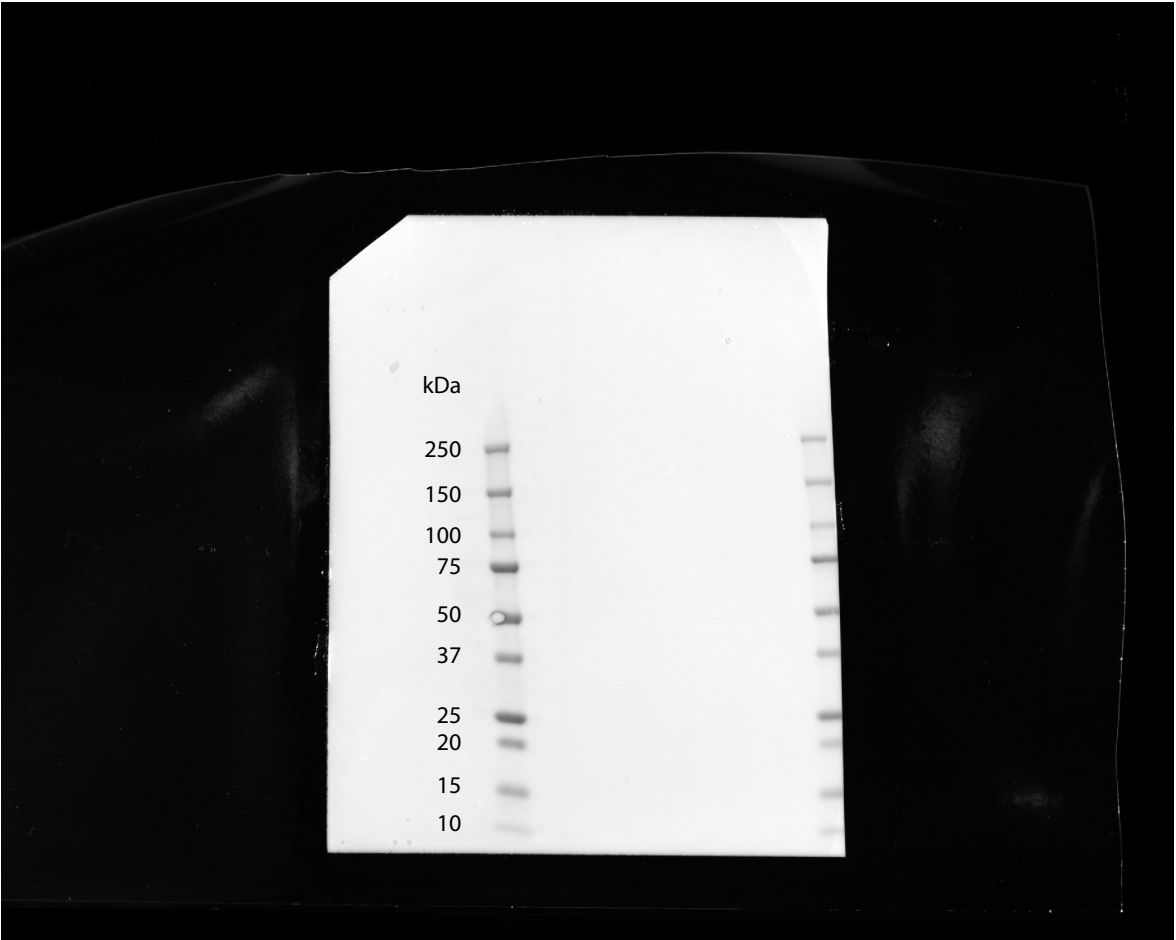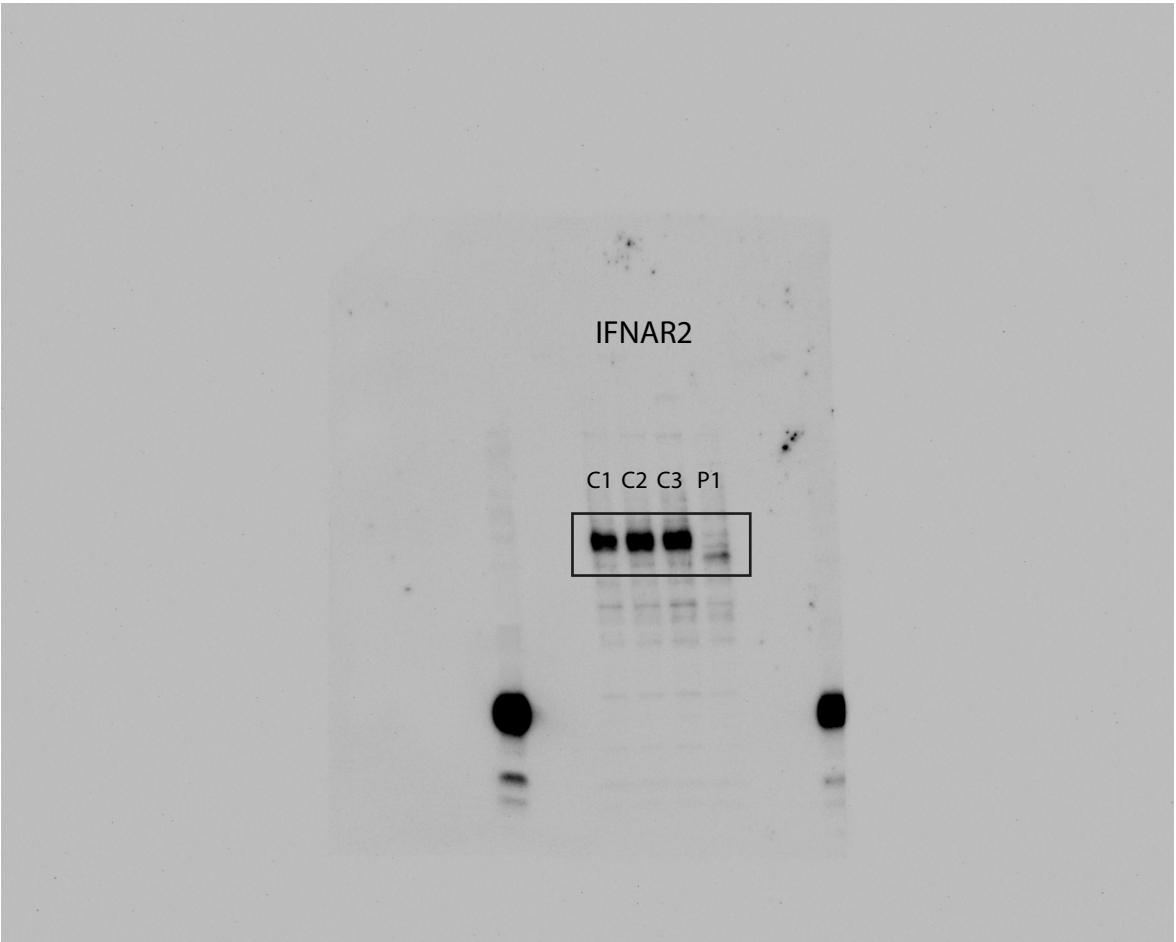

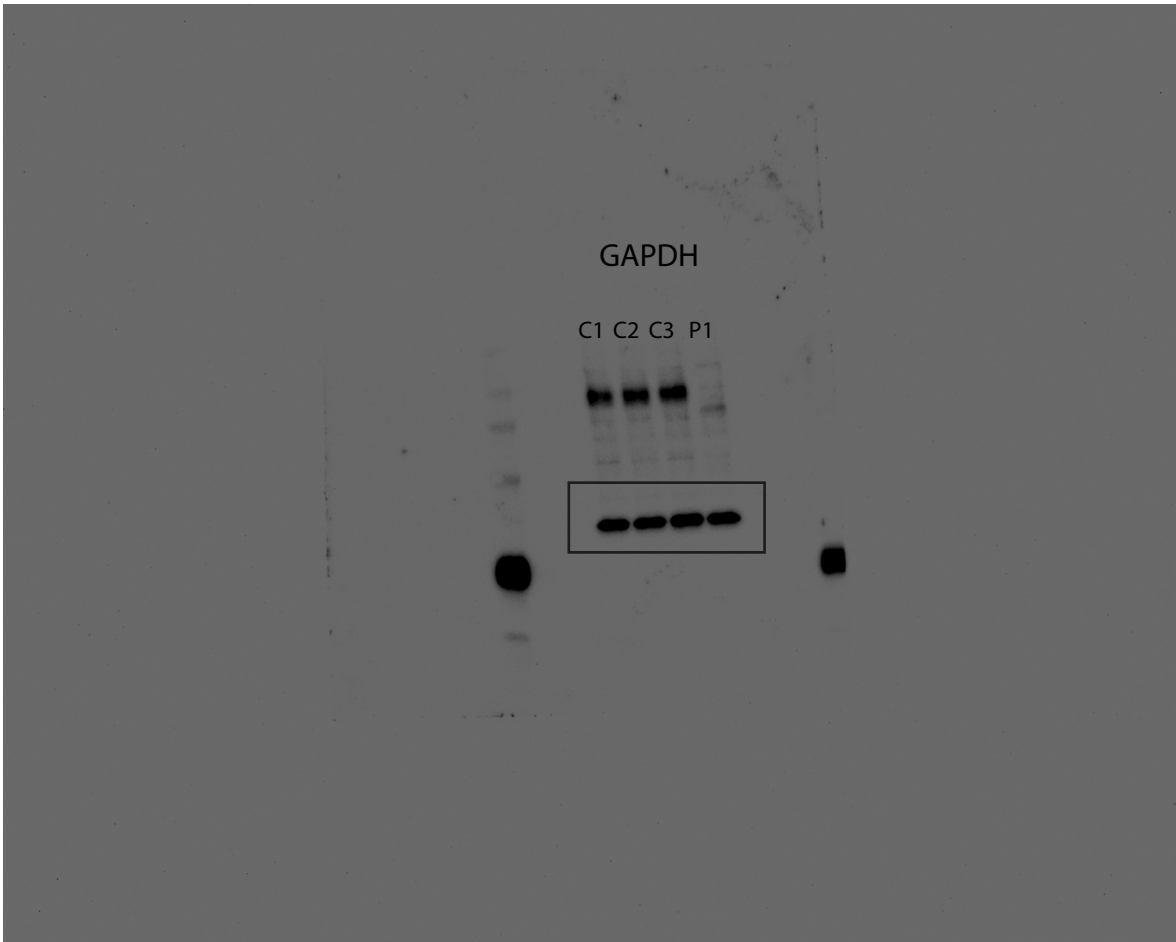

Supplement: SourceData F1 — contains original blots for Fig. 1. [file JEM_20212427_SourceDataF1.pdf]

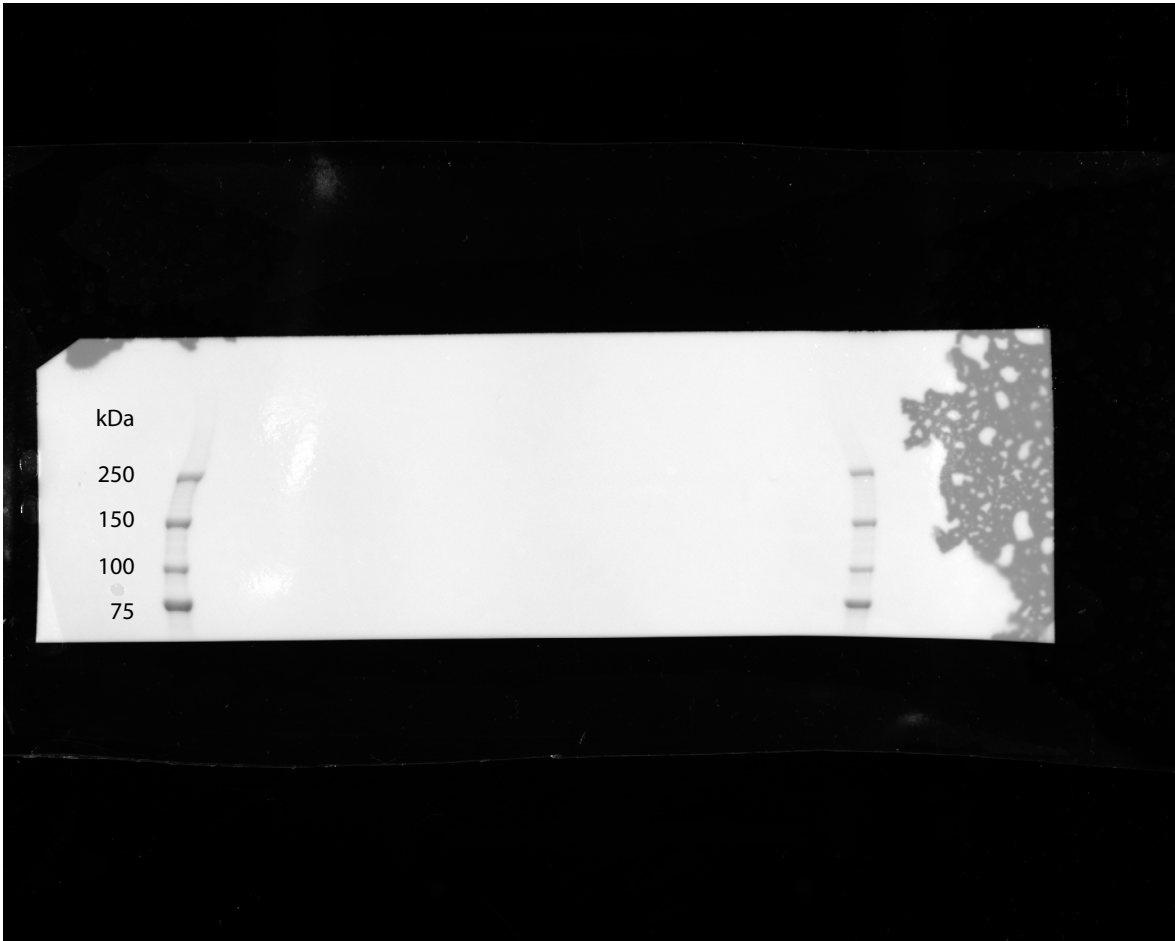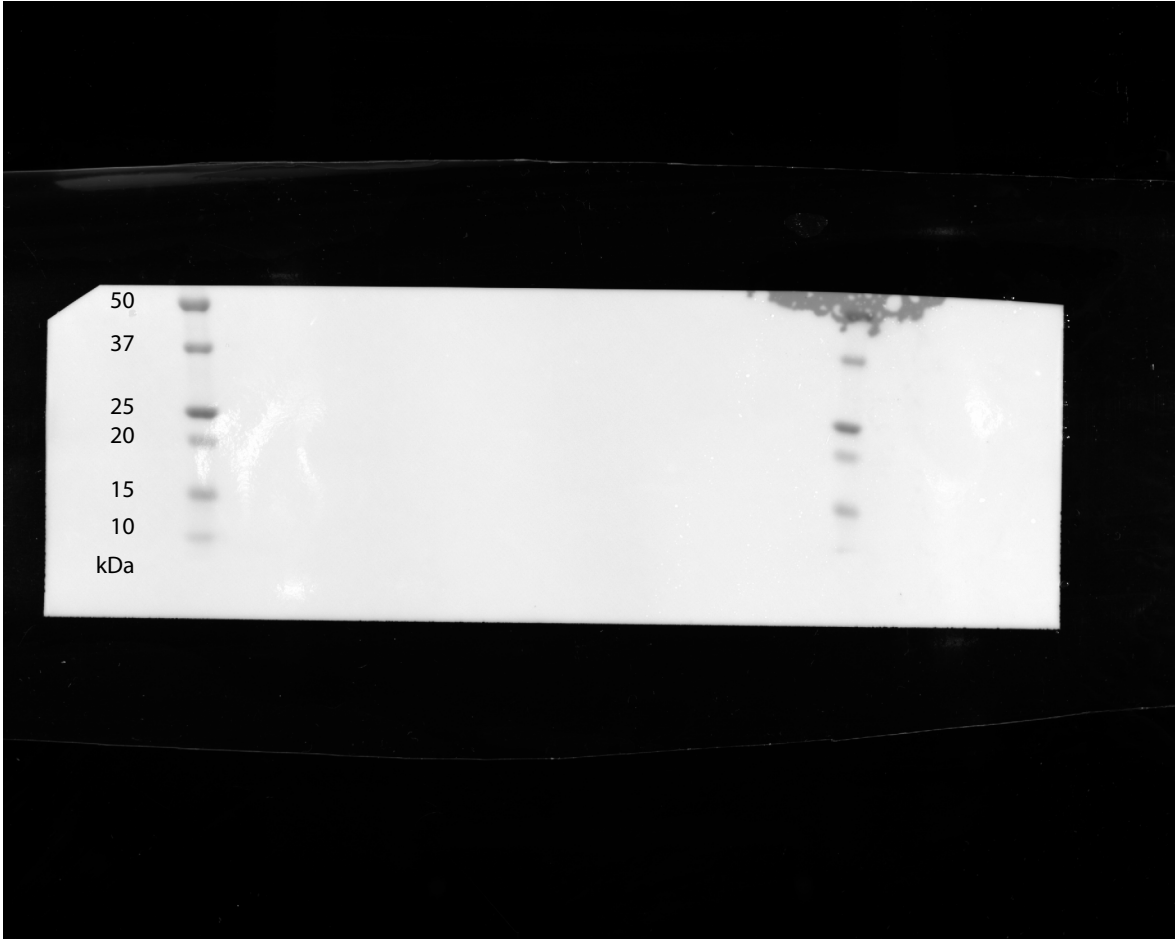

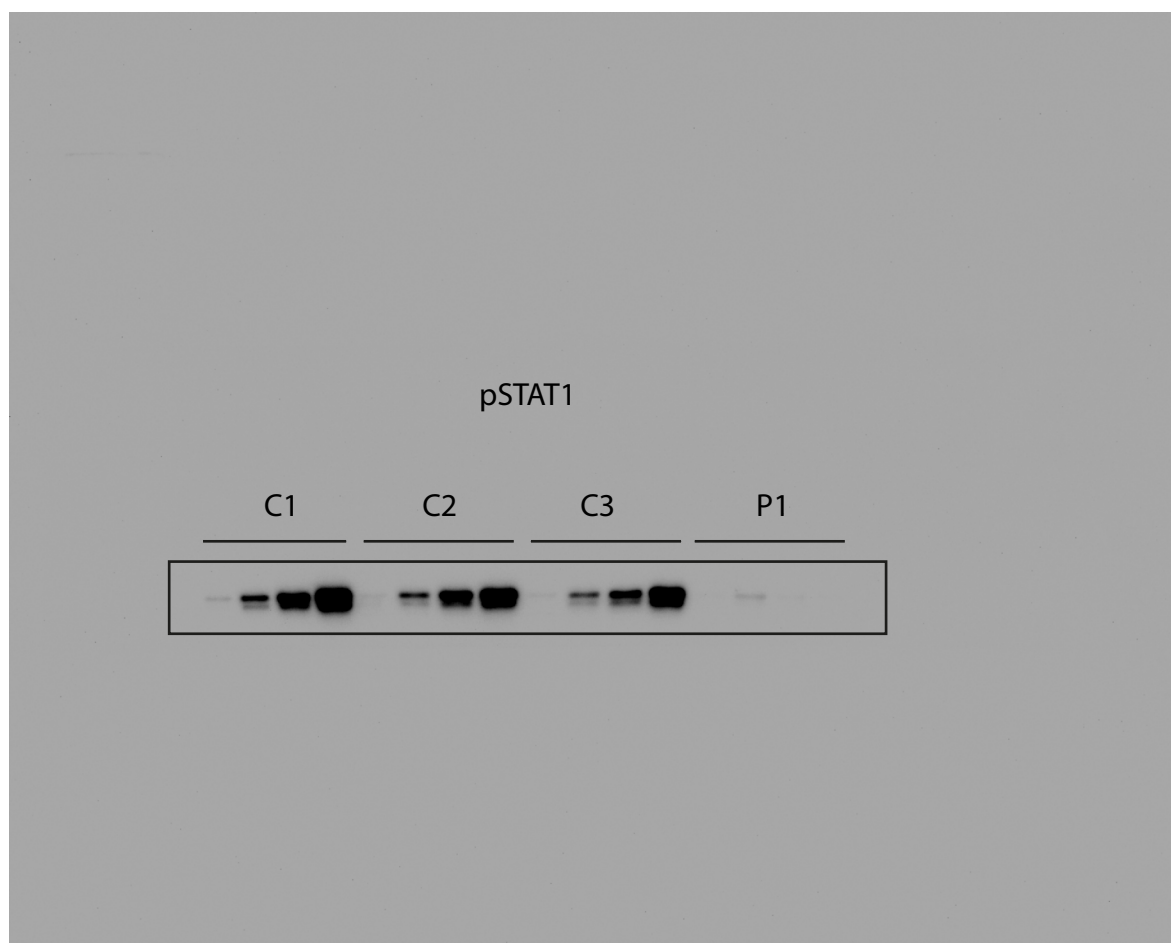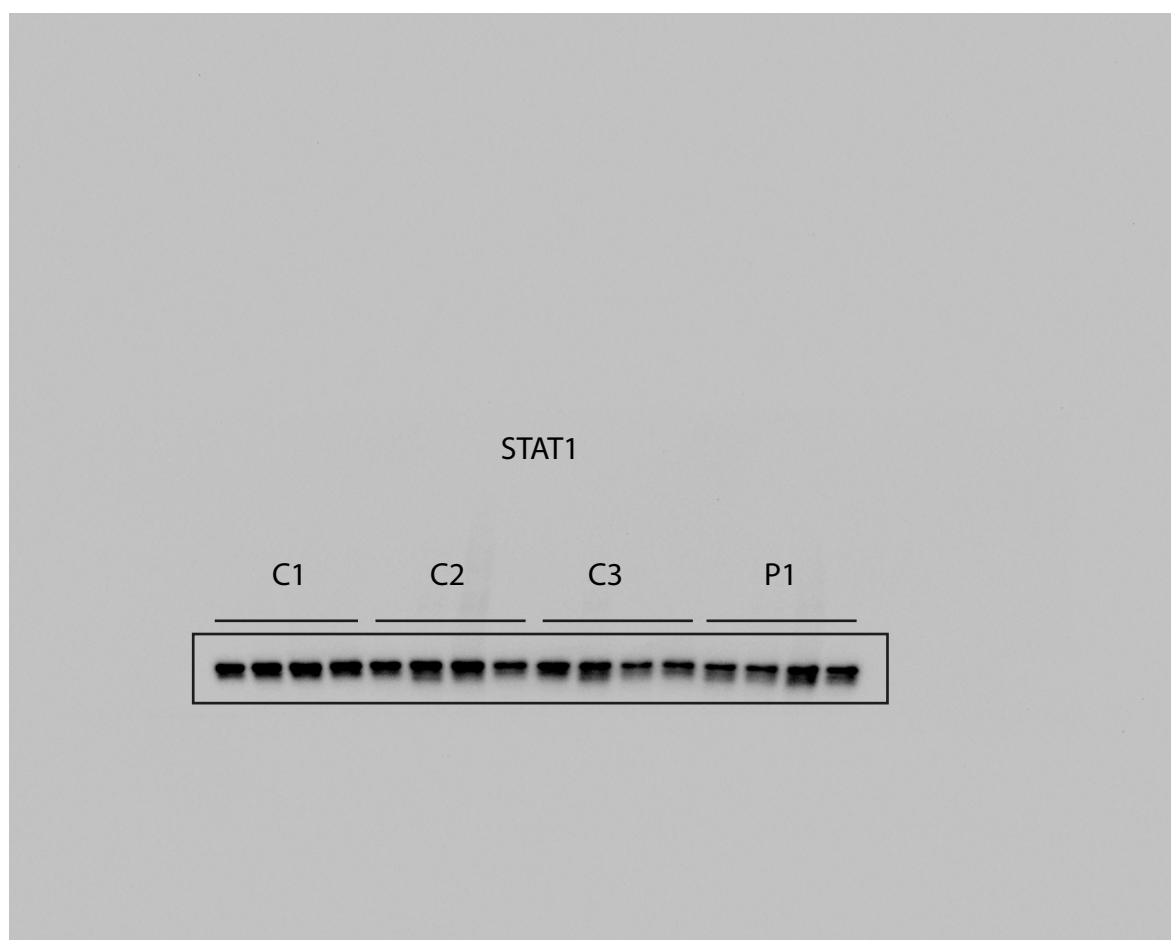

Source data: Figure 2A

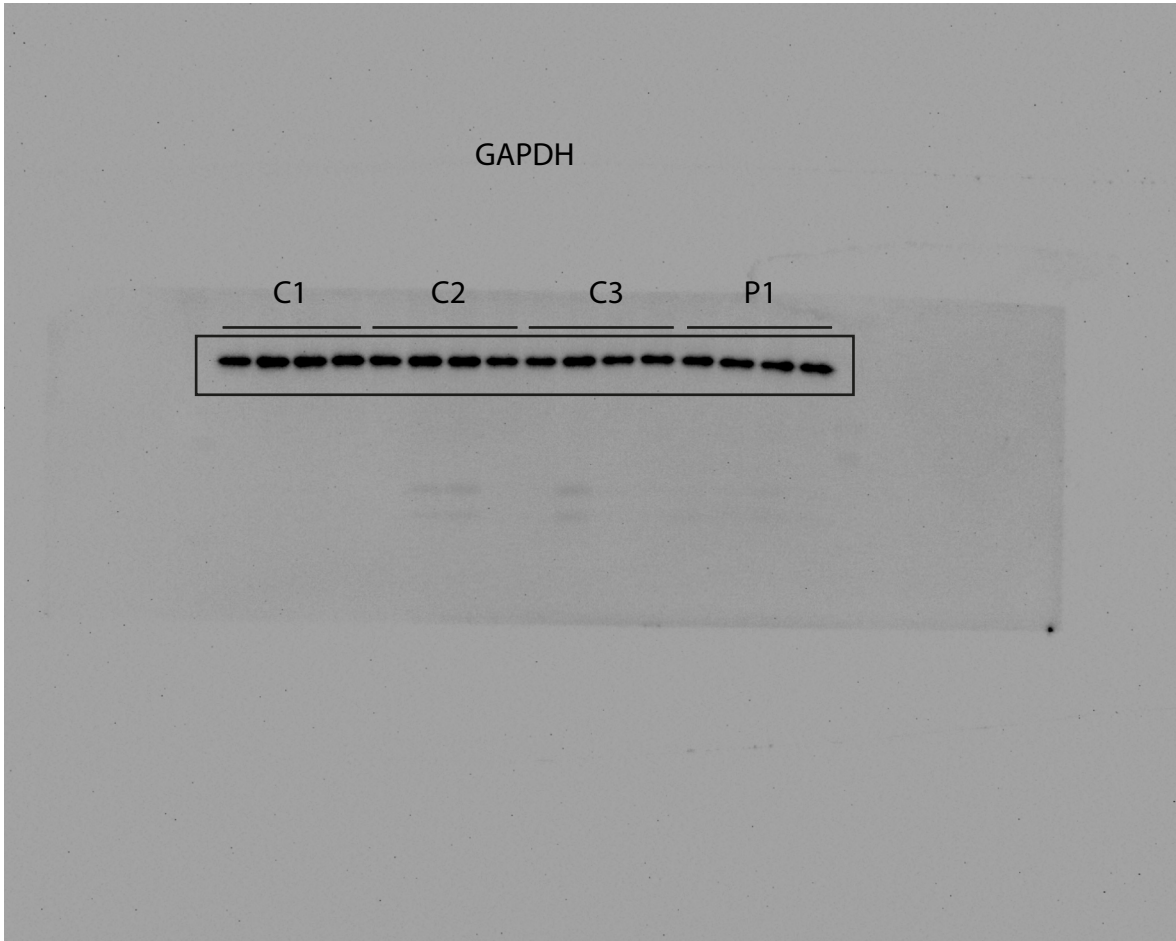

Source data: Figure 2B

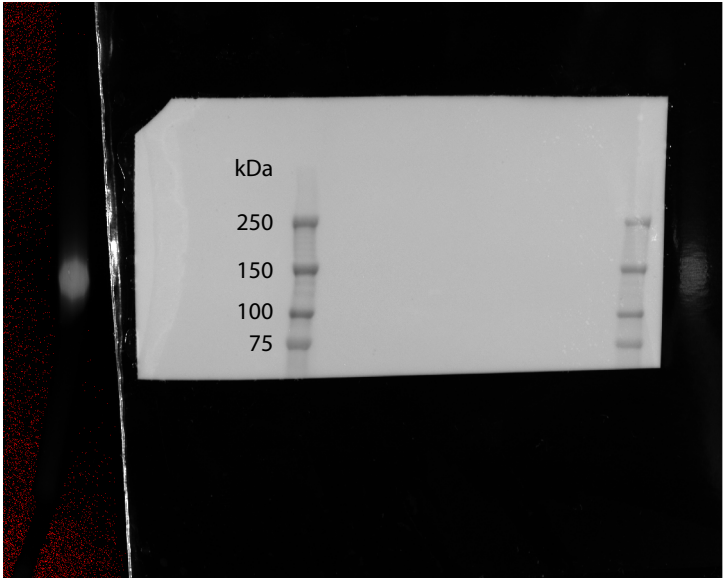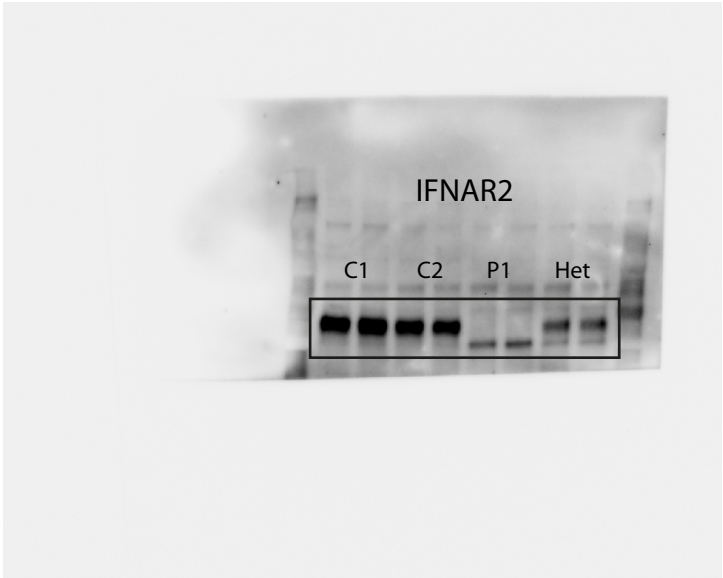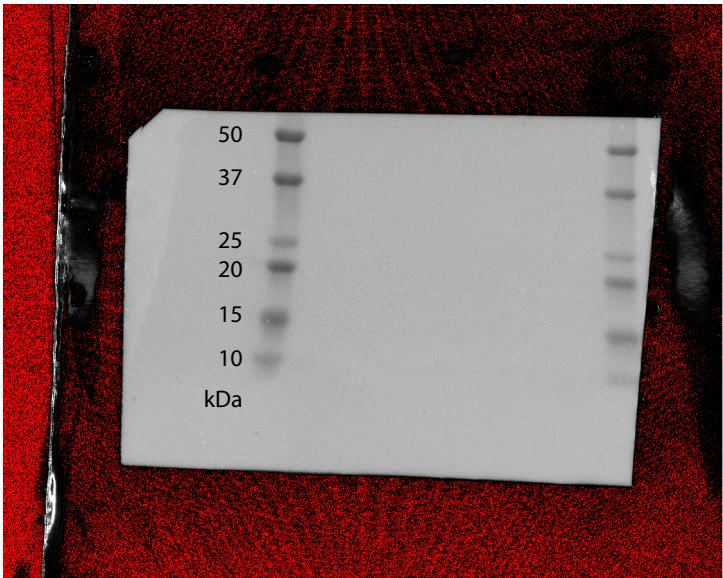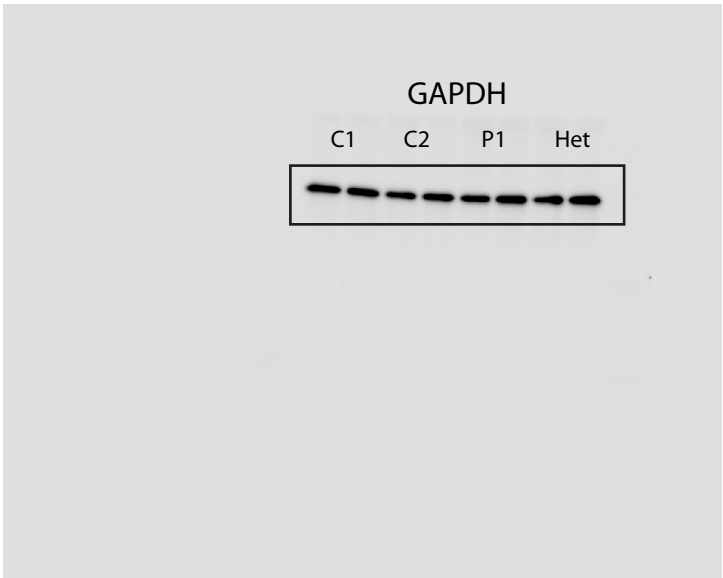

Source data: Figure 2B

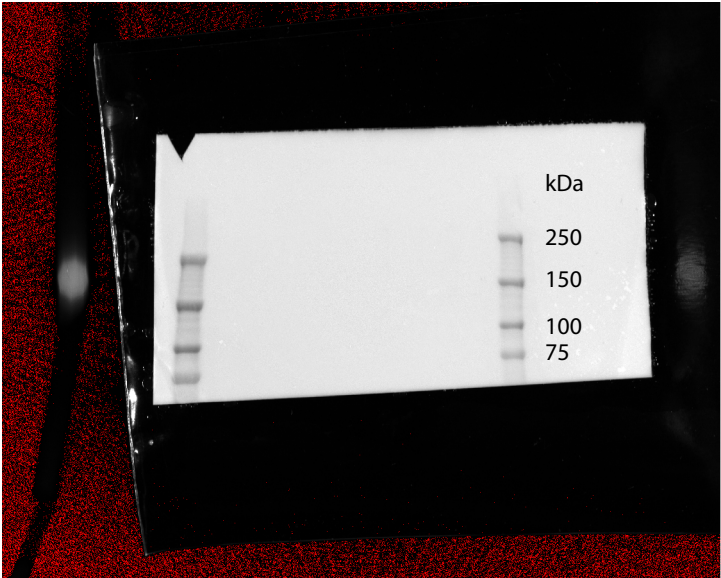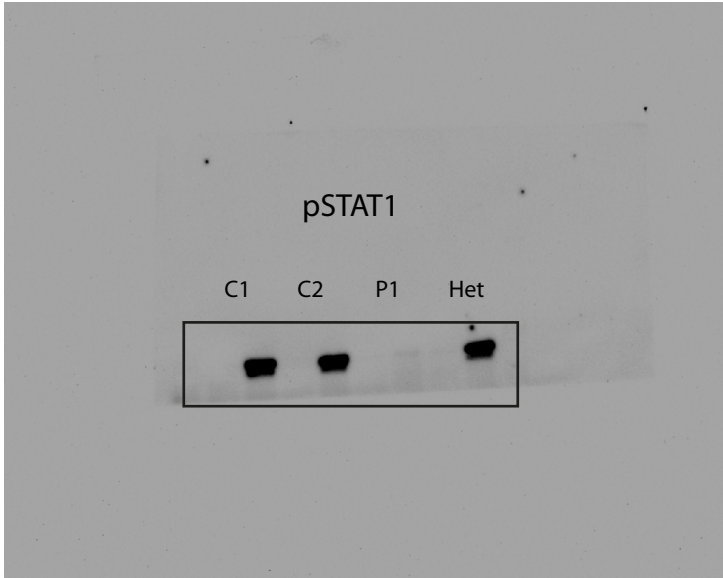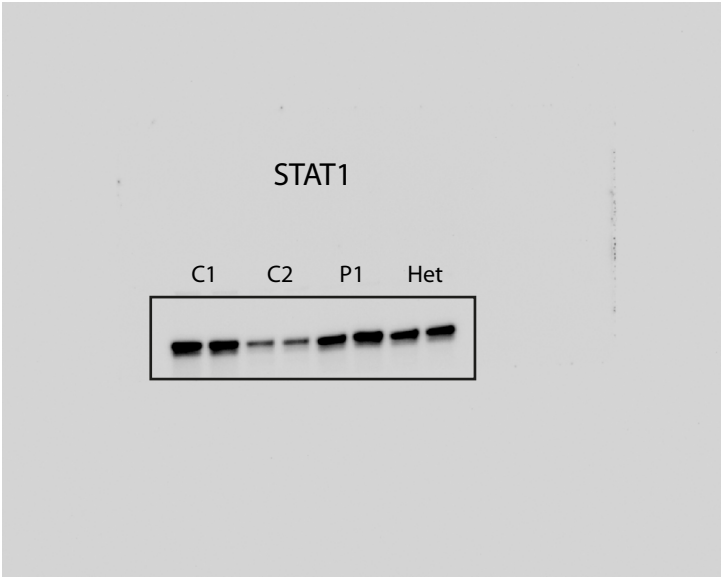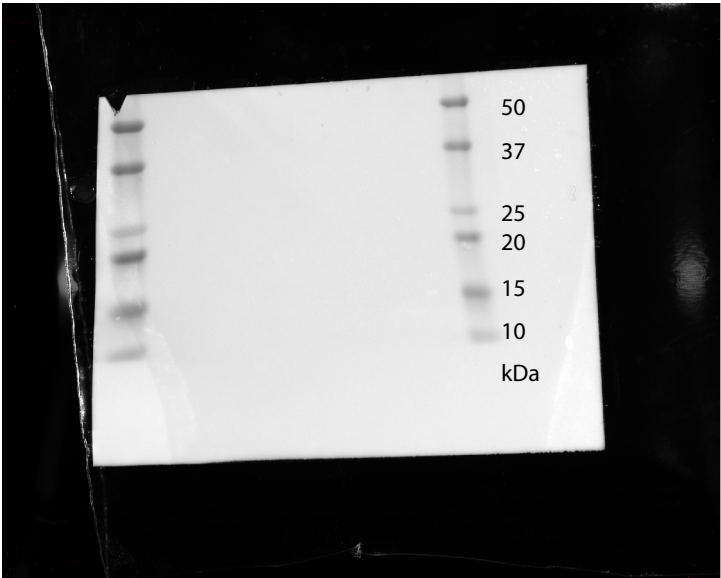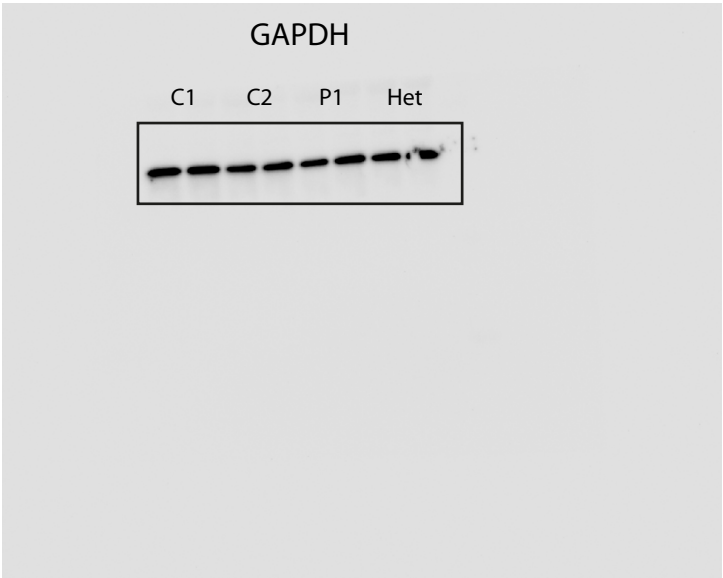

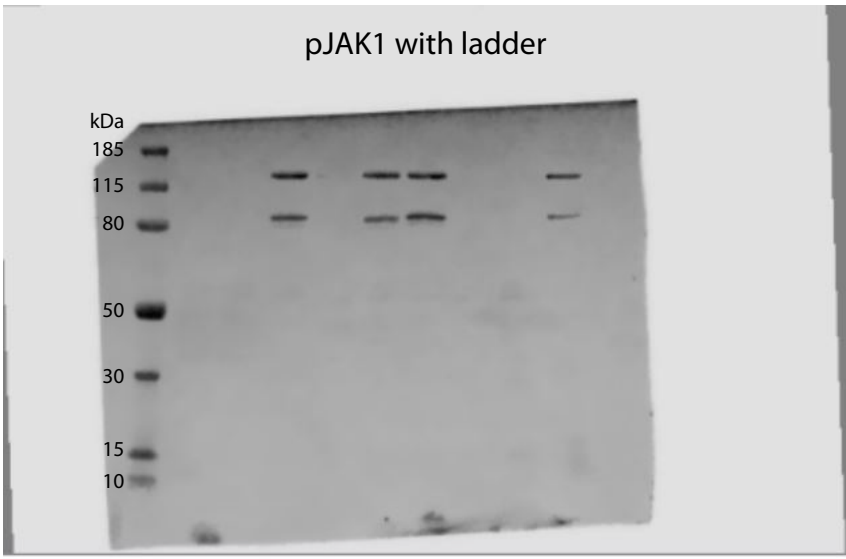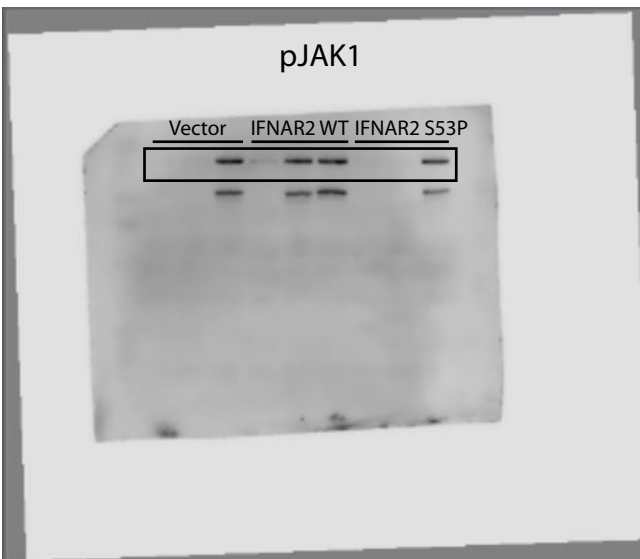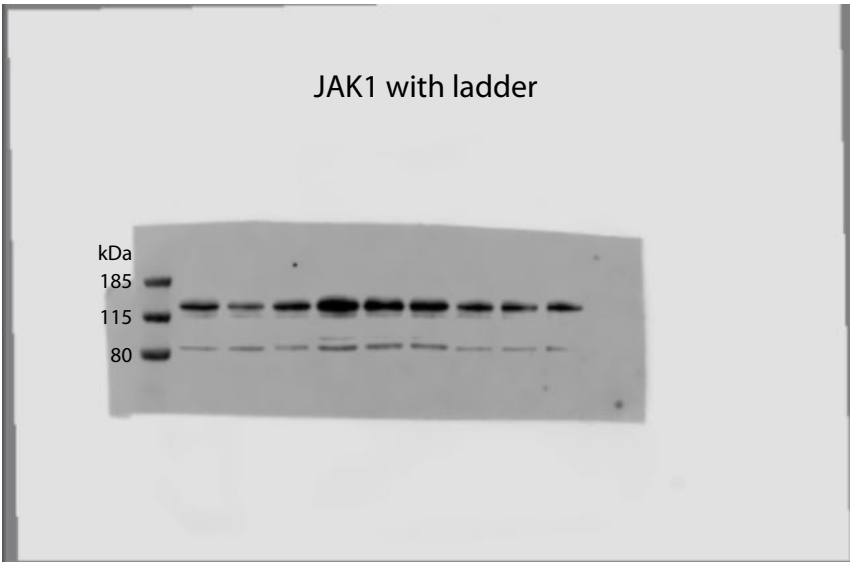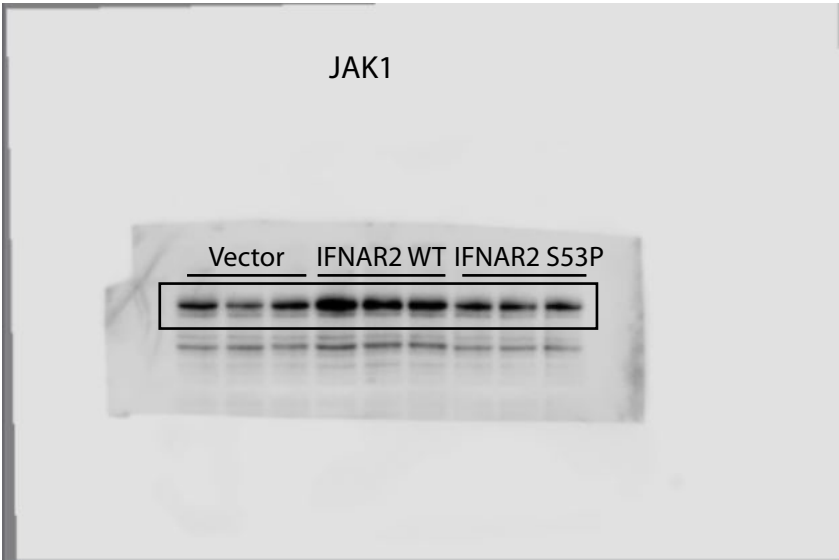

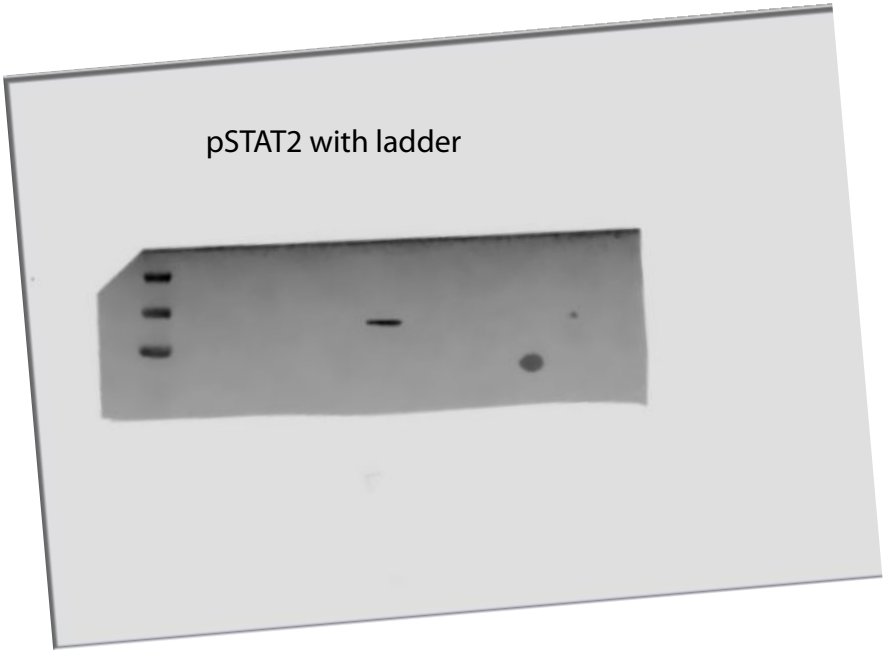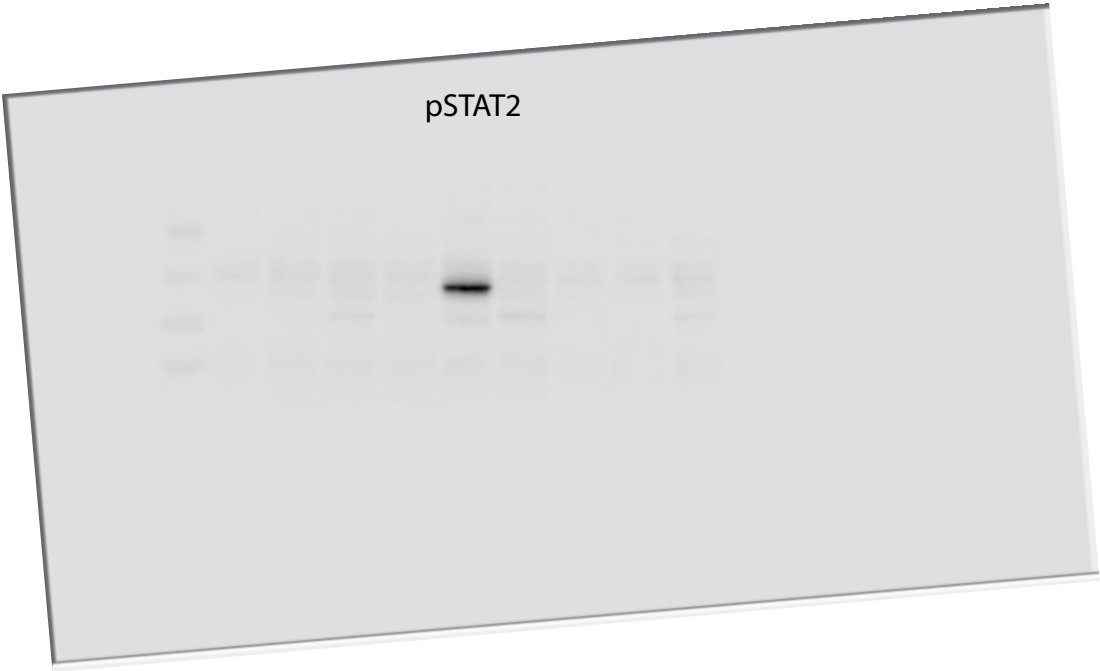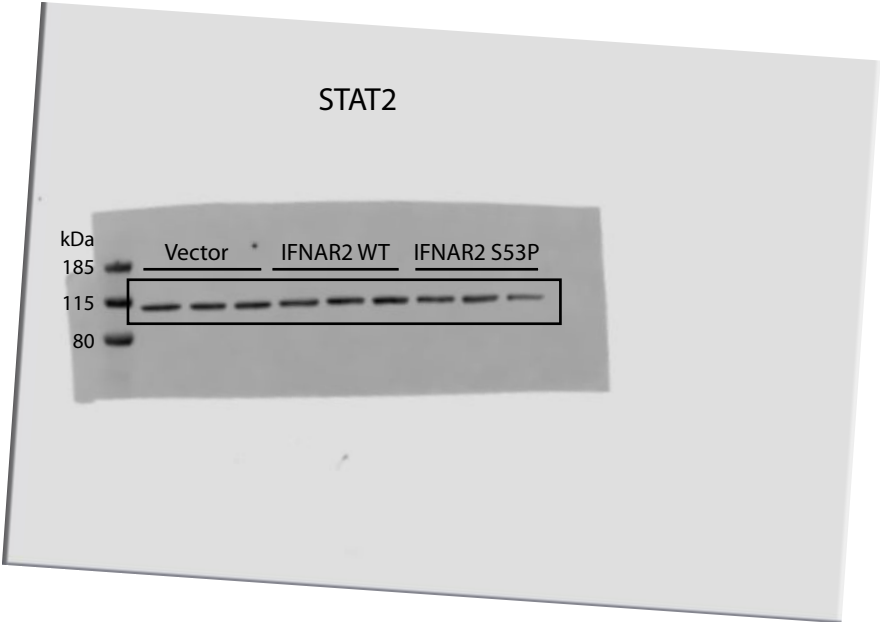

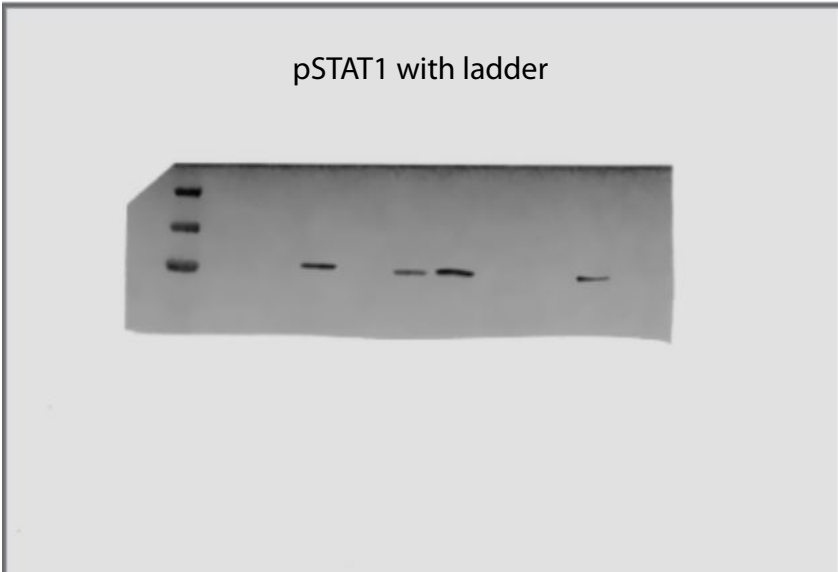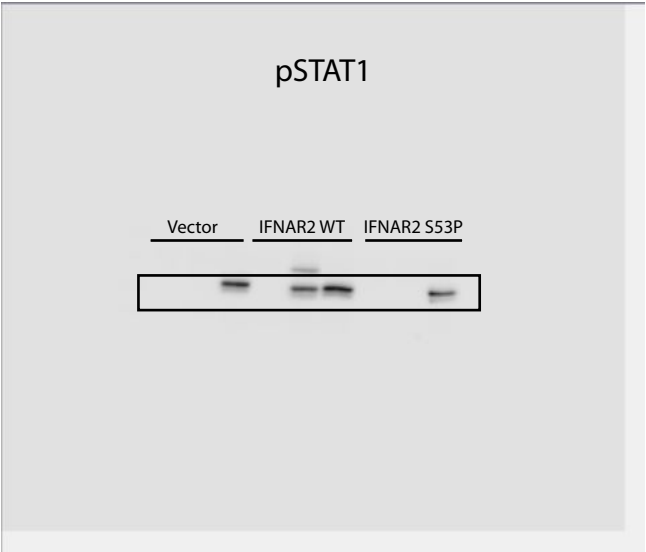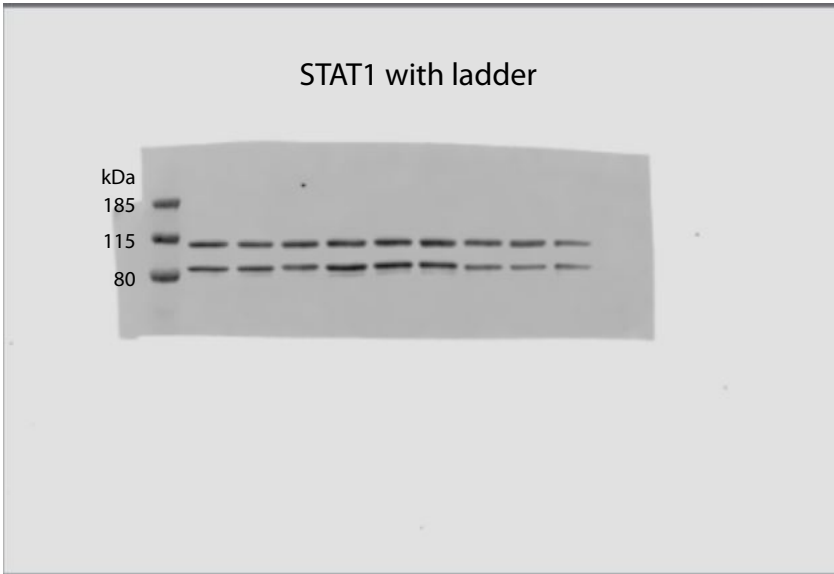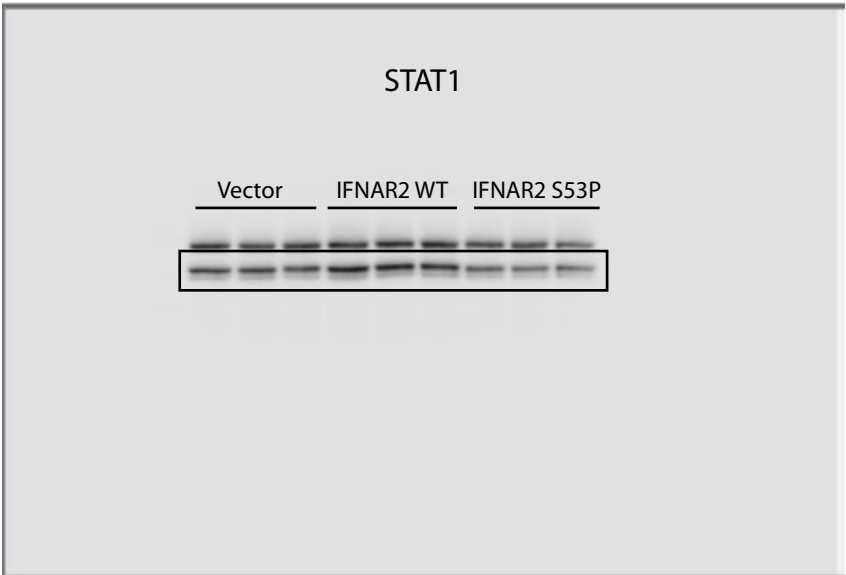

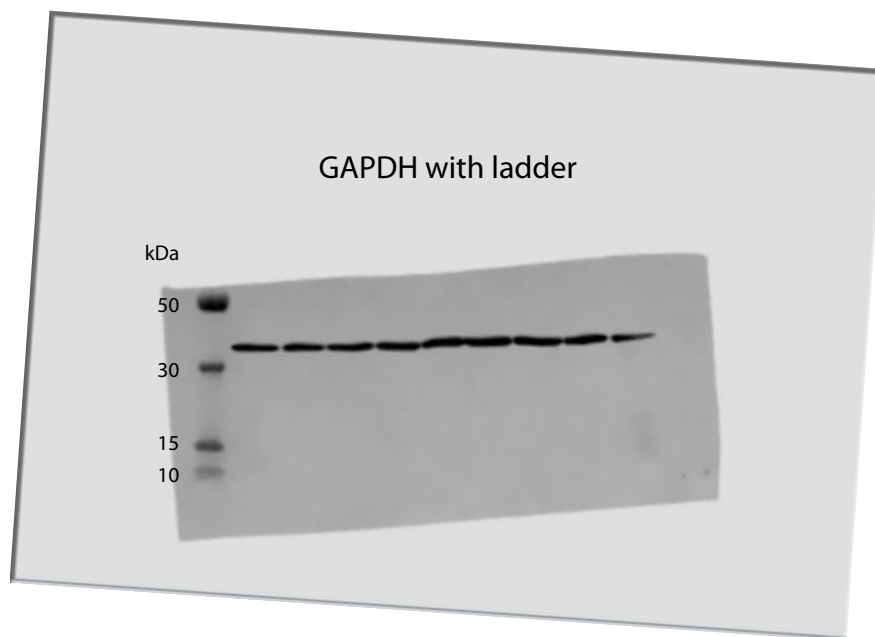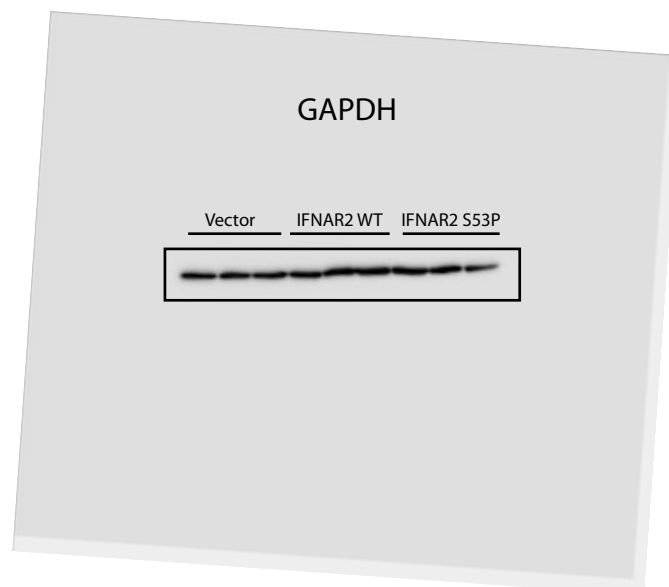

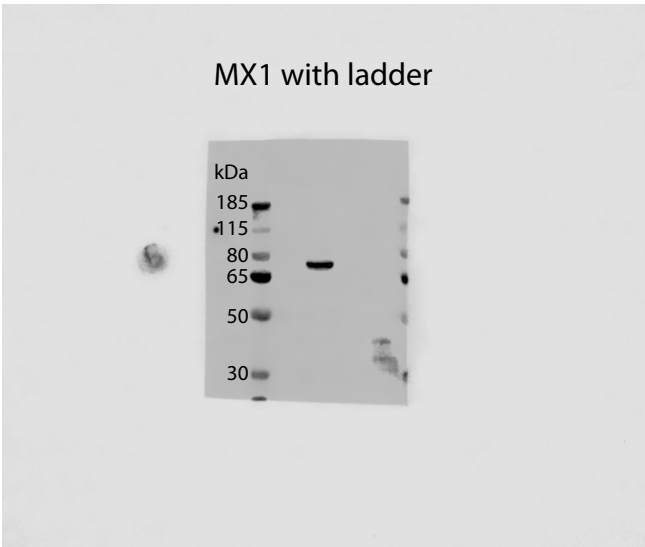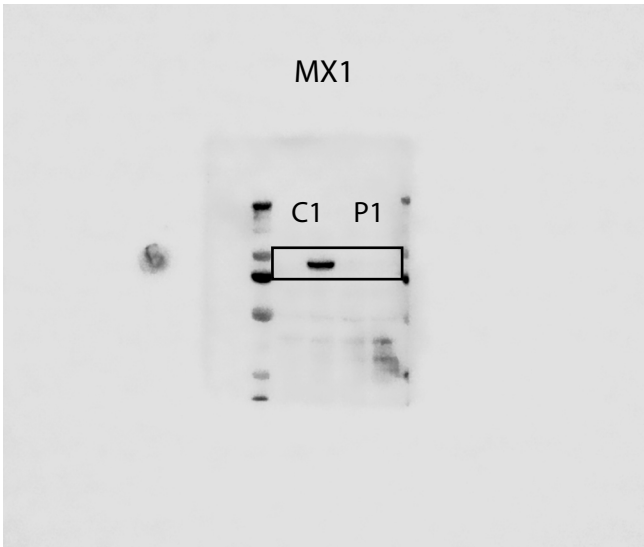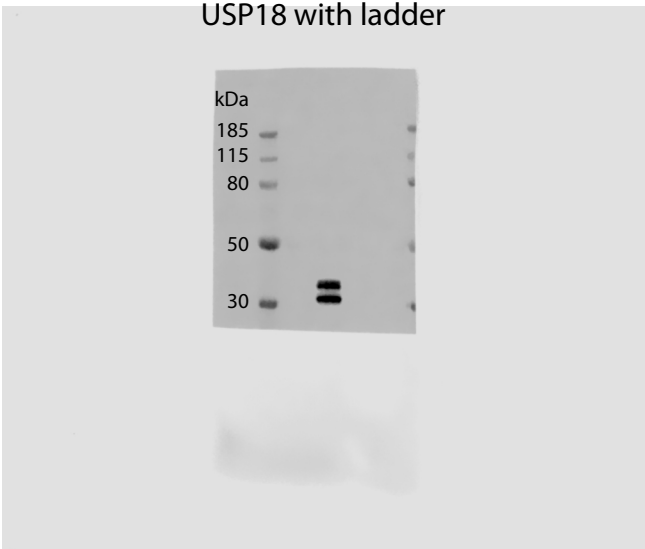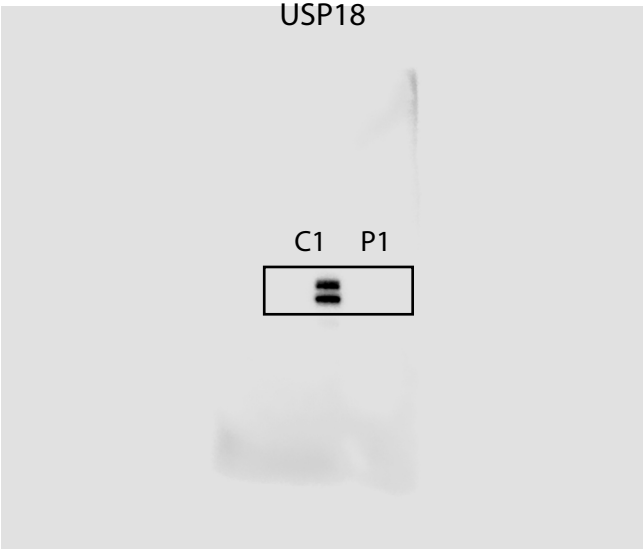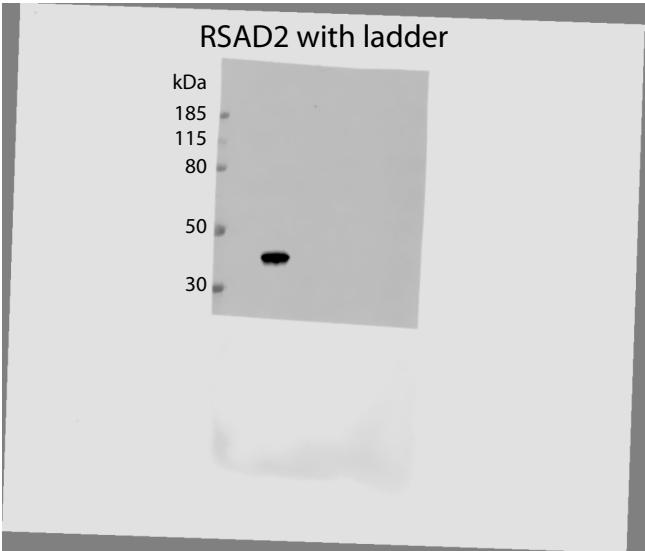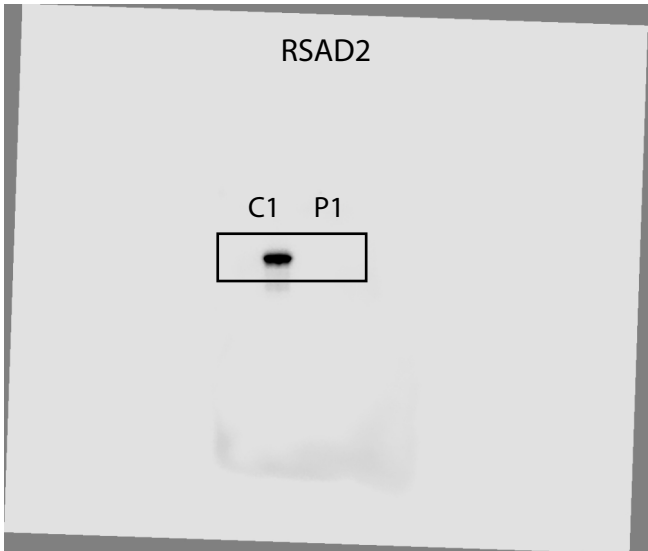

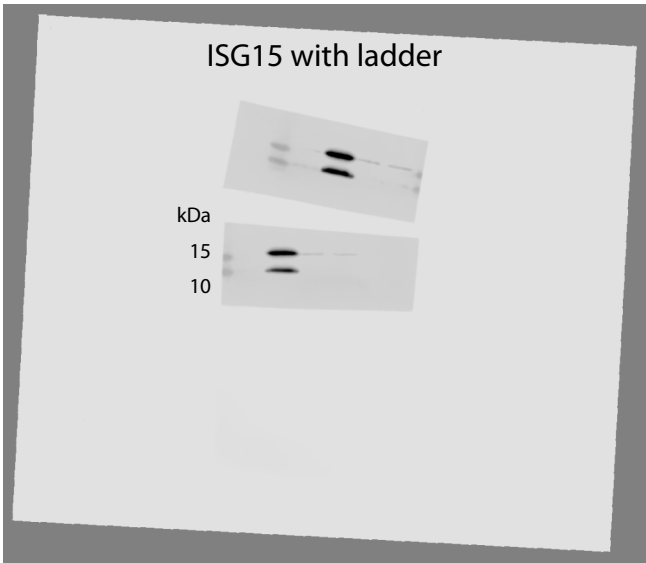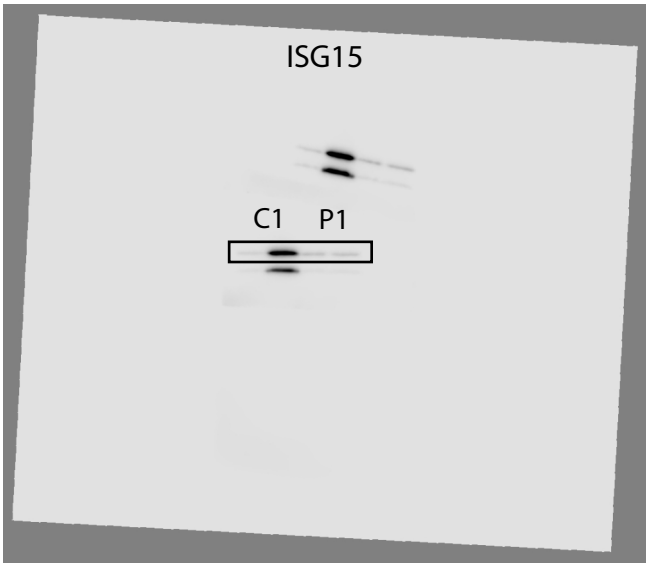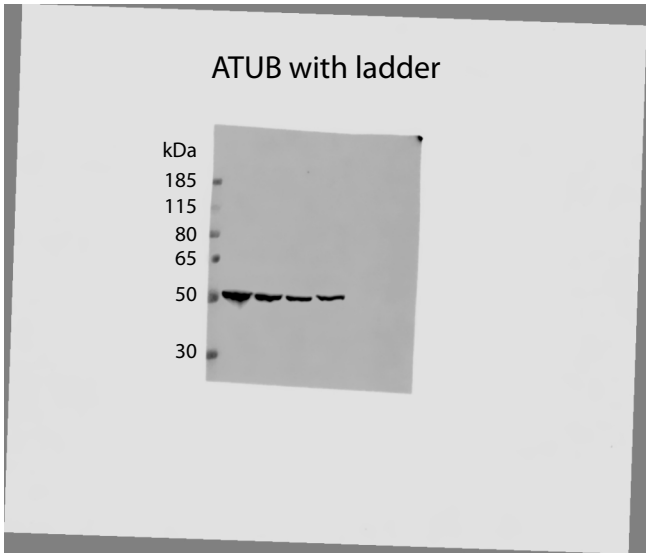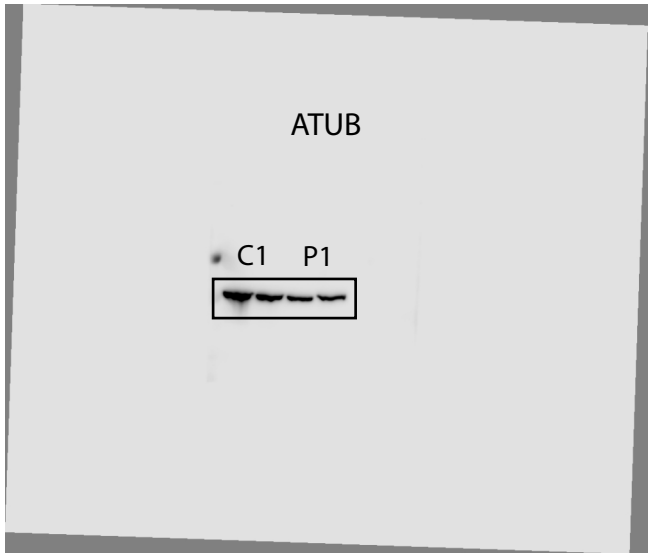

Supplement: SourceData F2 — contains original blots for Fig. 2. [file JEM_20212427_SourceDataF2.pdf]

Source data: Figure 3B

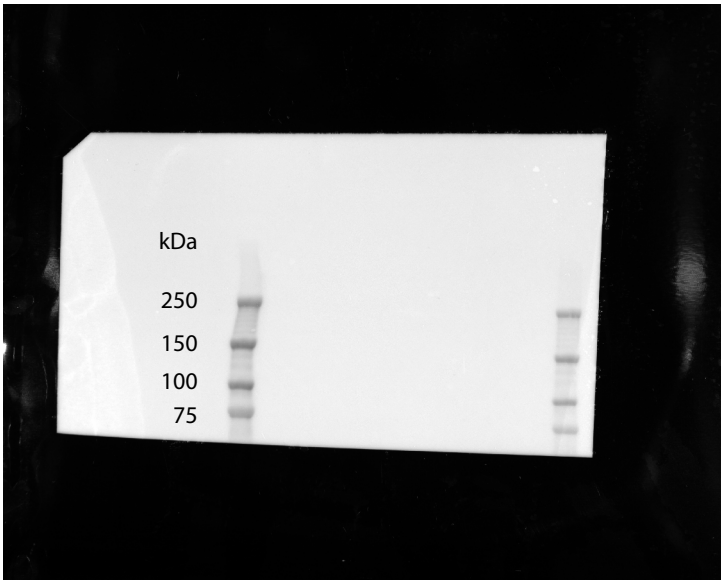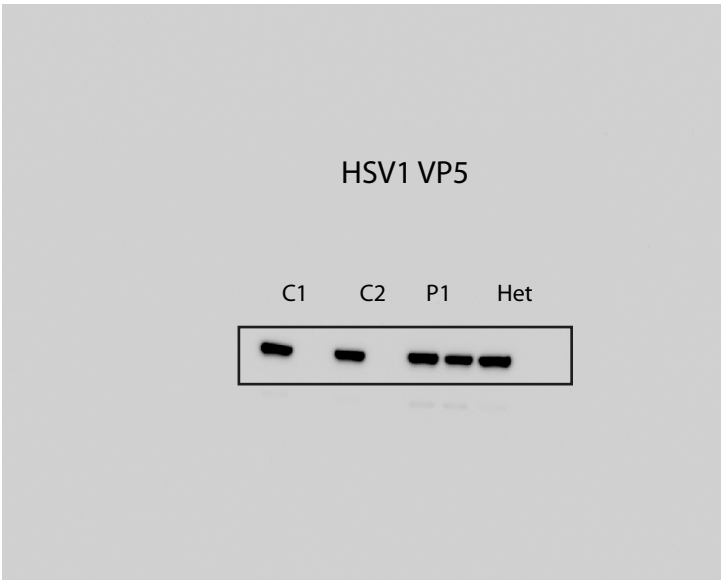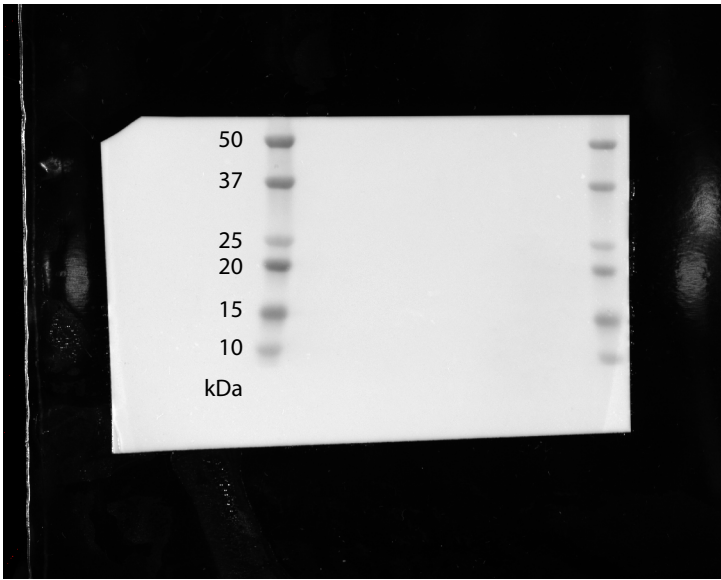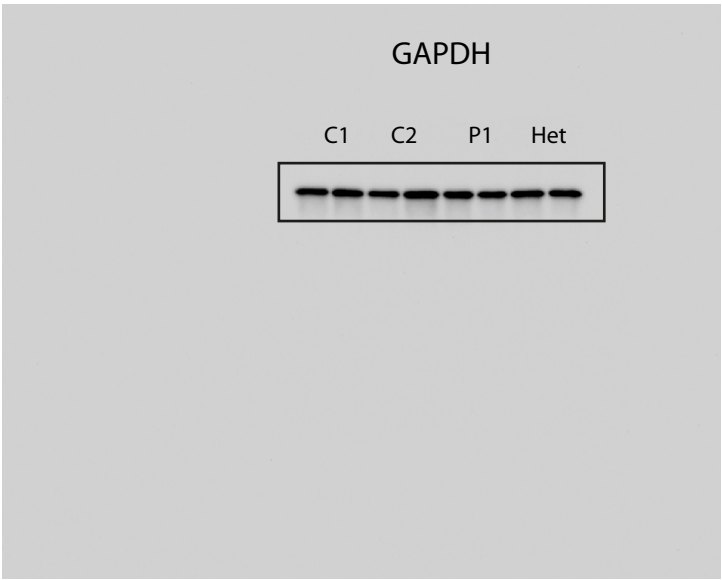

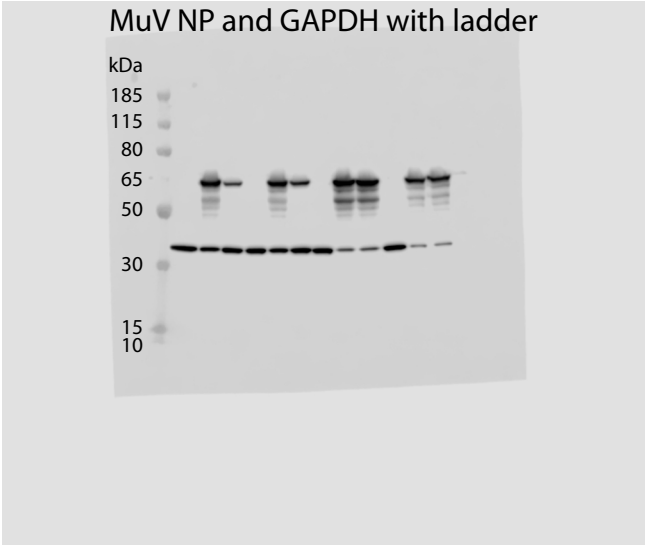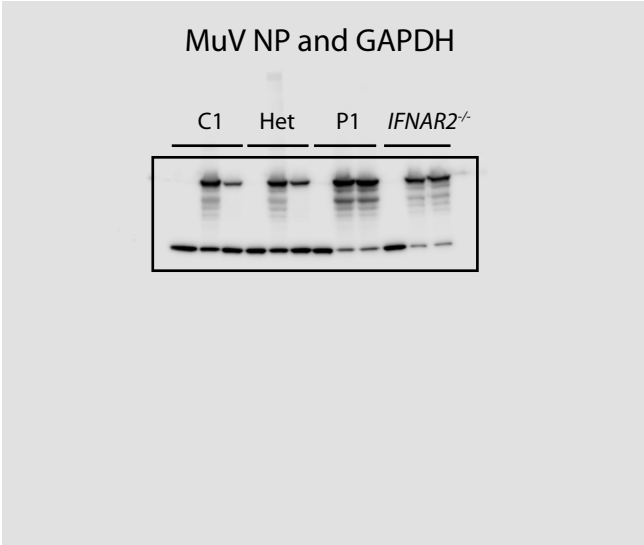

Supplement: SourceData F3 — contains original blots for Fig. 3. [file JEM_20212427_SourceDataF3.pdf]

Source data: Figure 4A

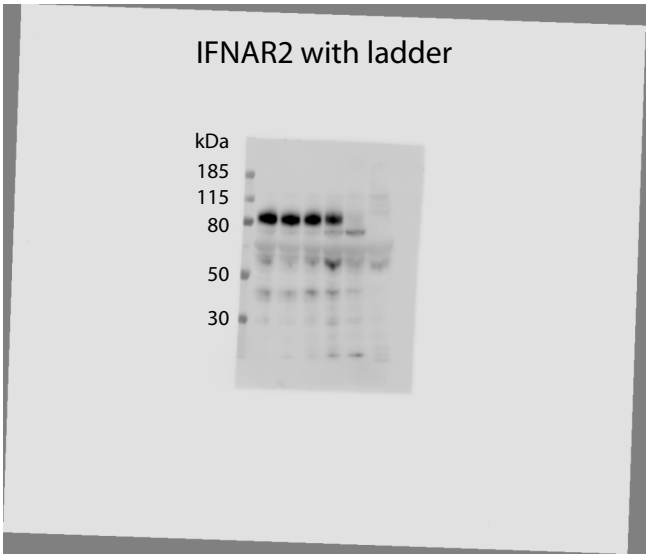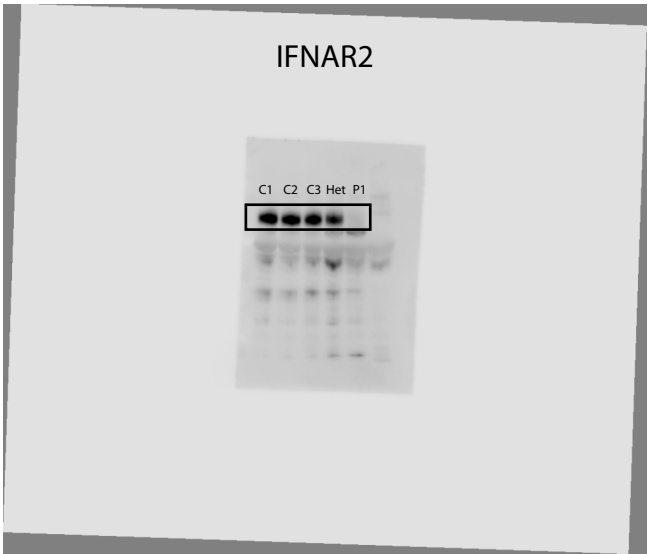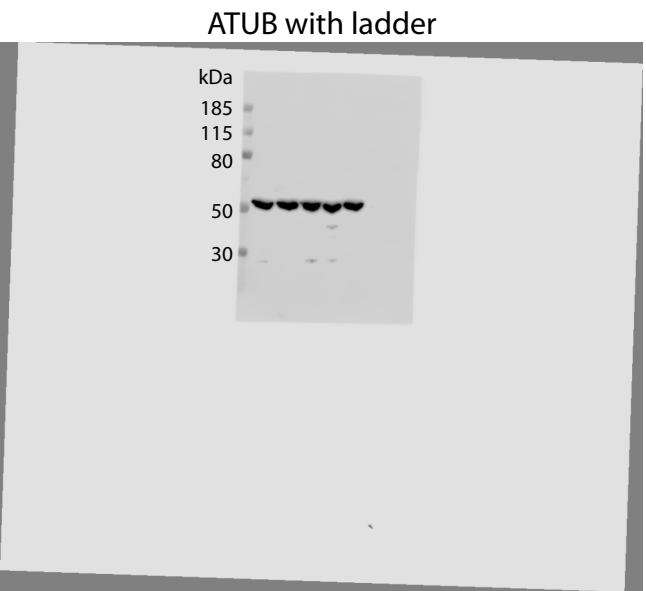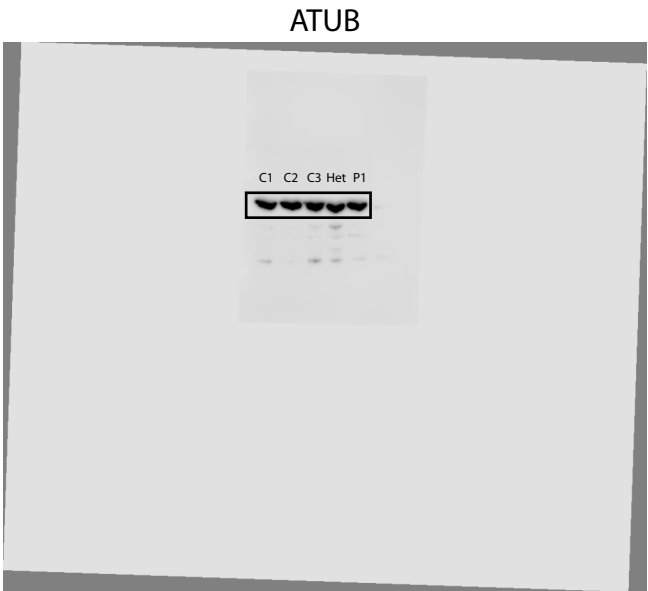

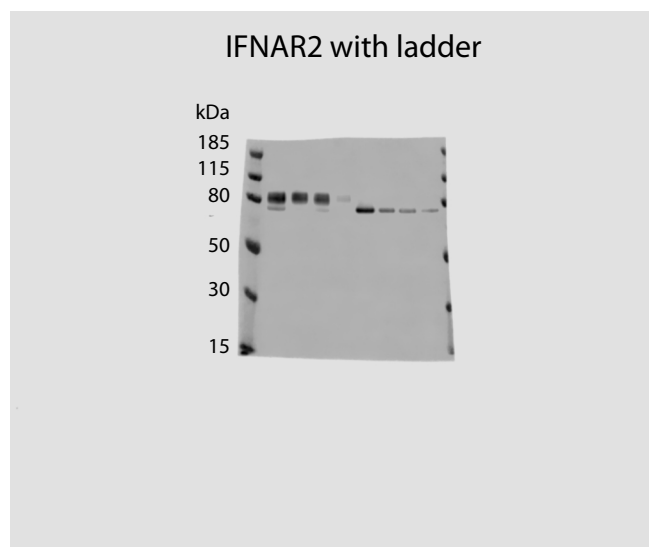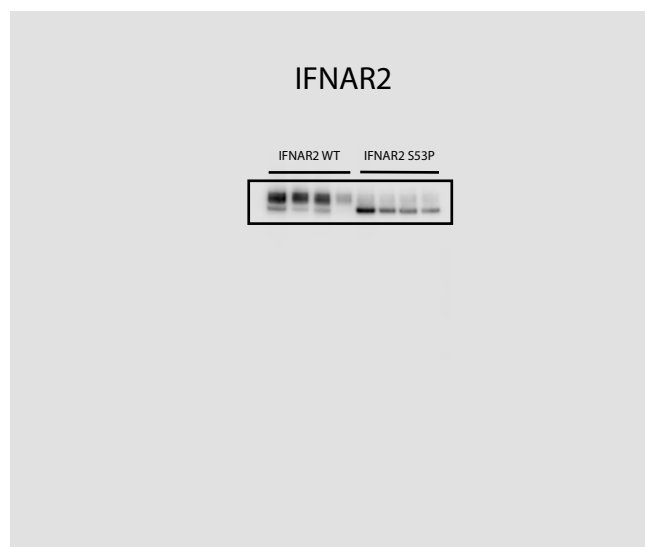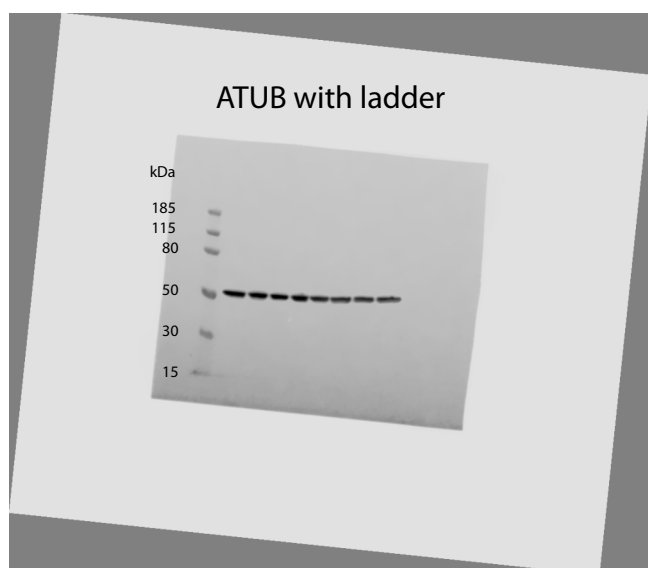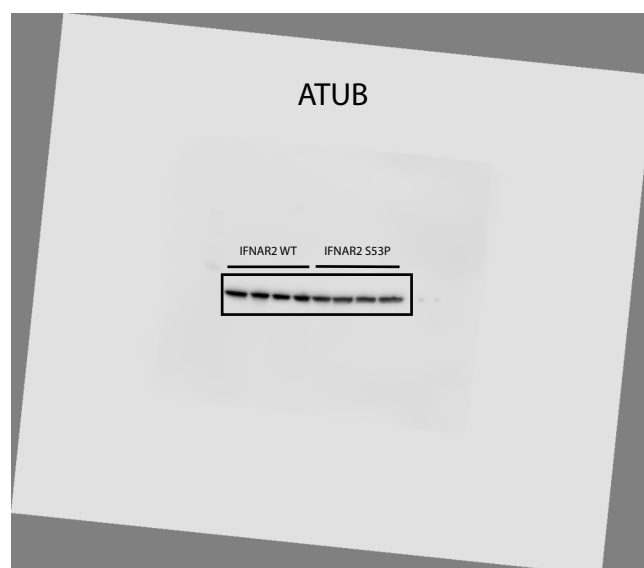

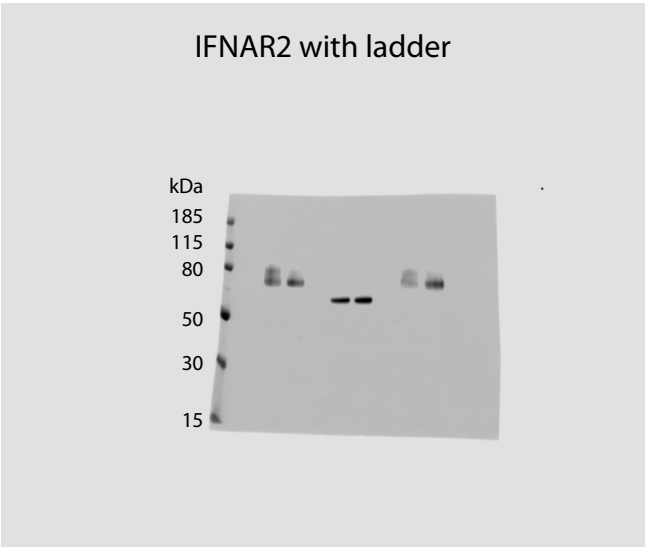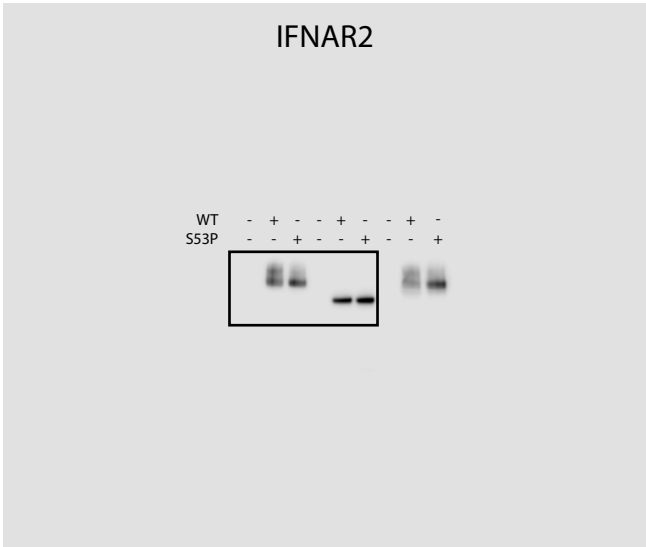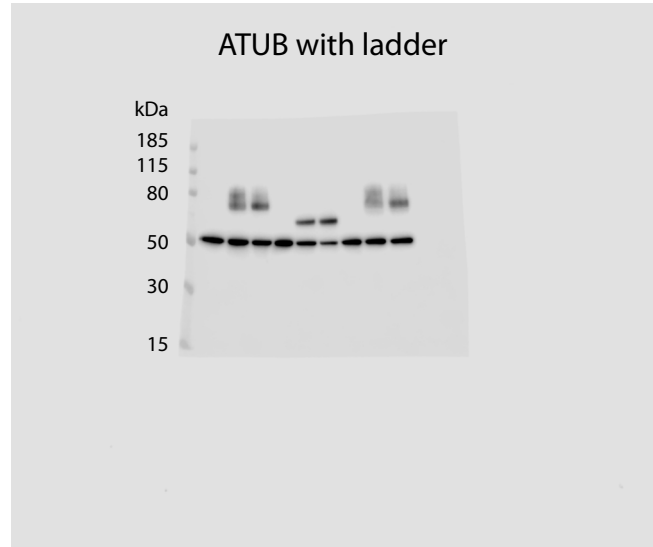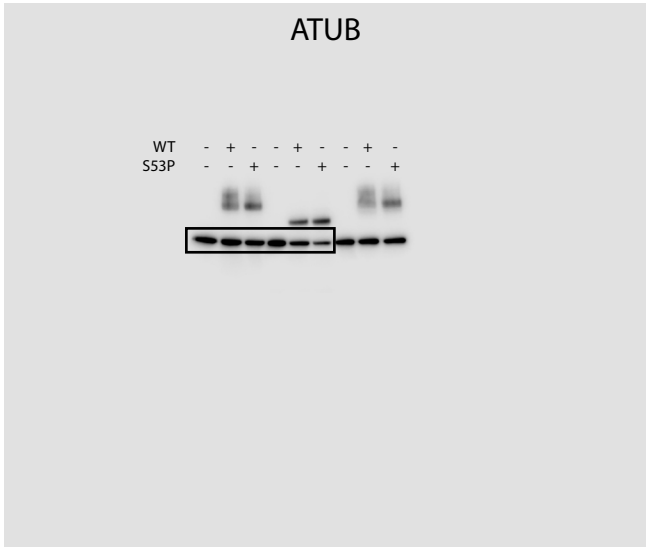

Source data: Figure 4E

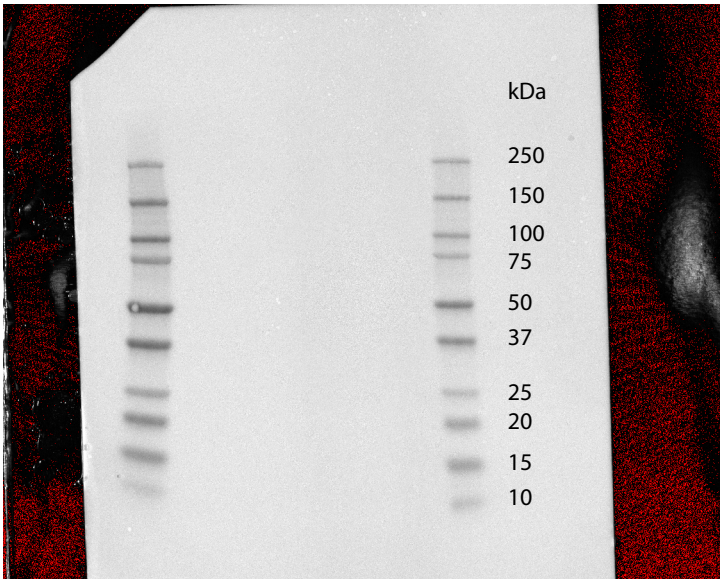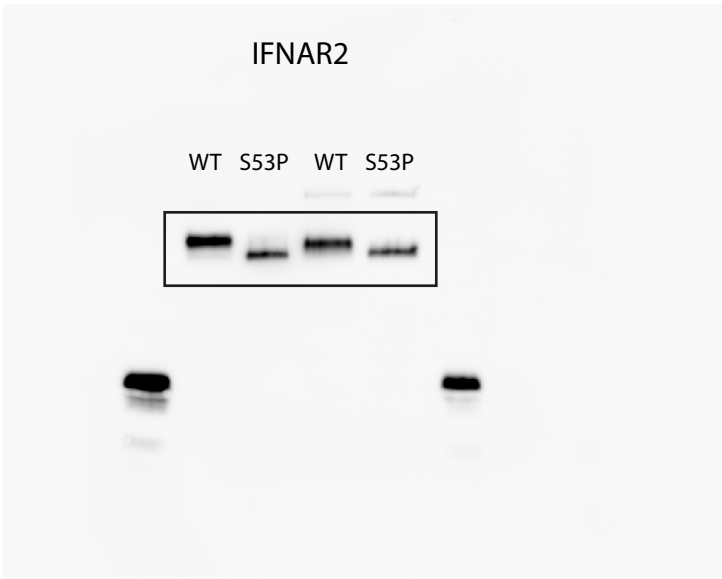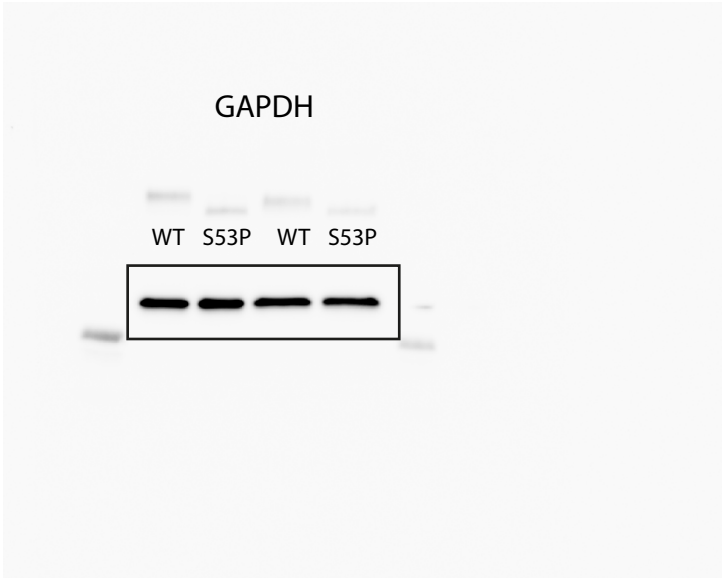

Supplement: SourceData F4 — contains original blots for Fig. 4. [file JEM_20212427_SourceDataF4.pdf]

Source data: Figure 5A

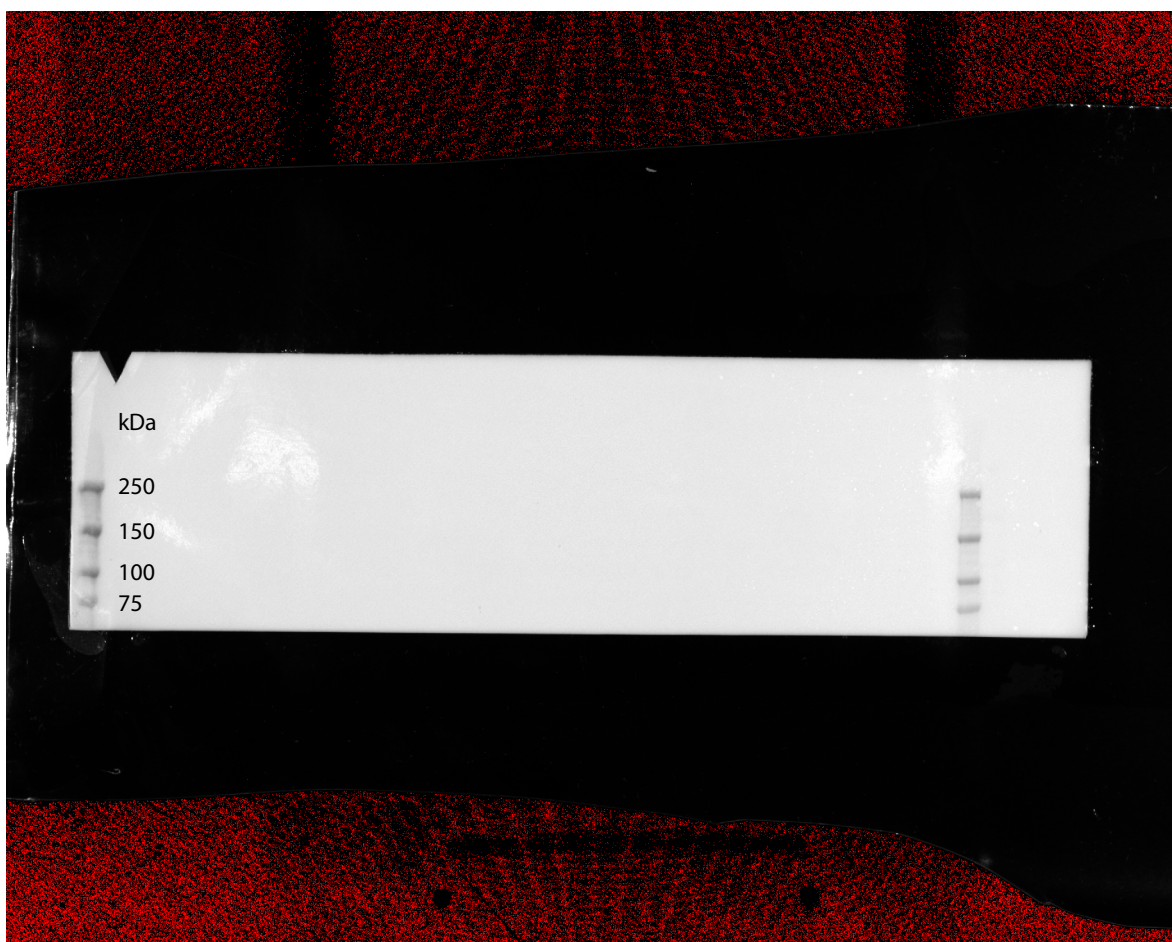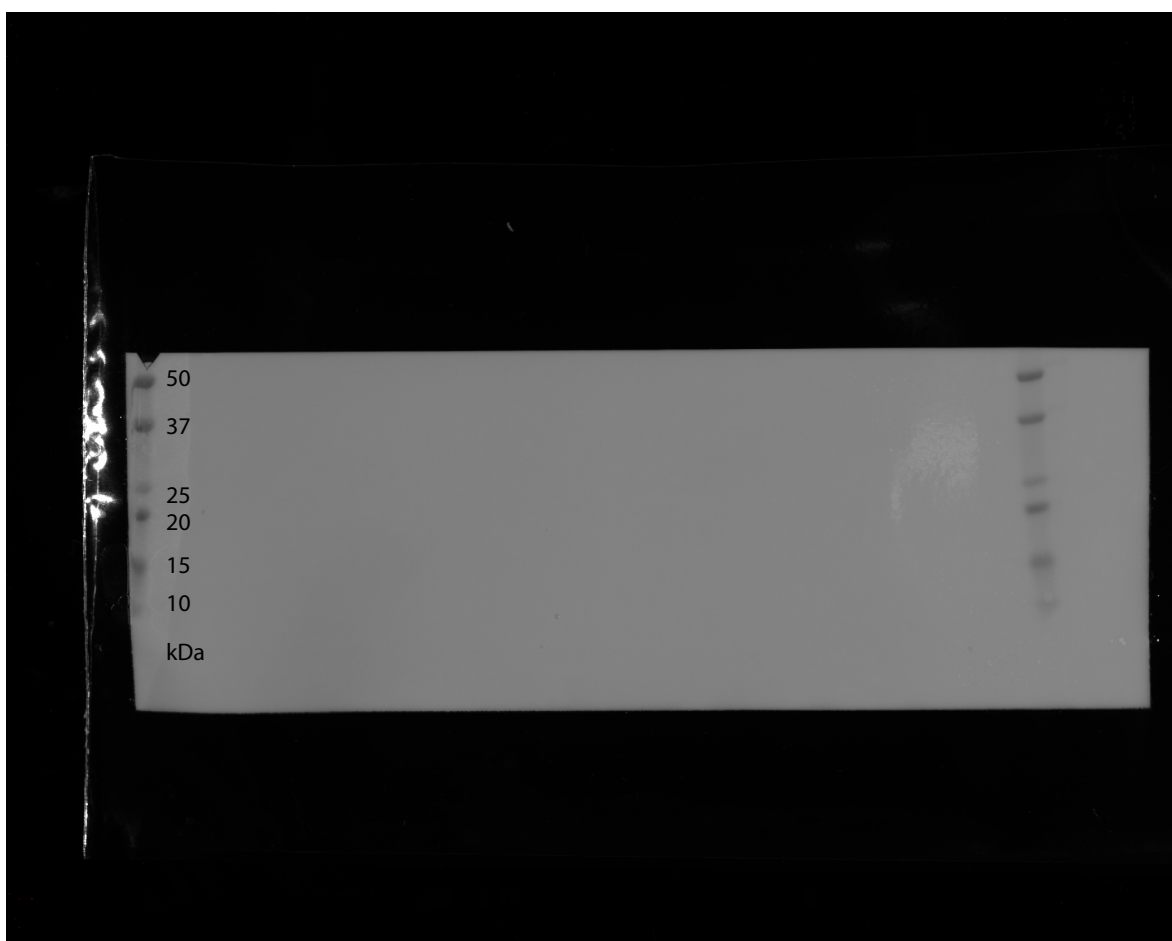

Source data: Figure 5A

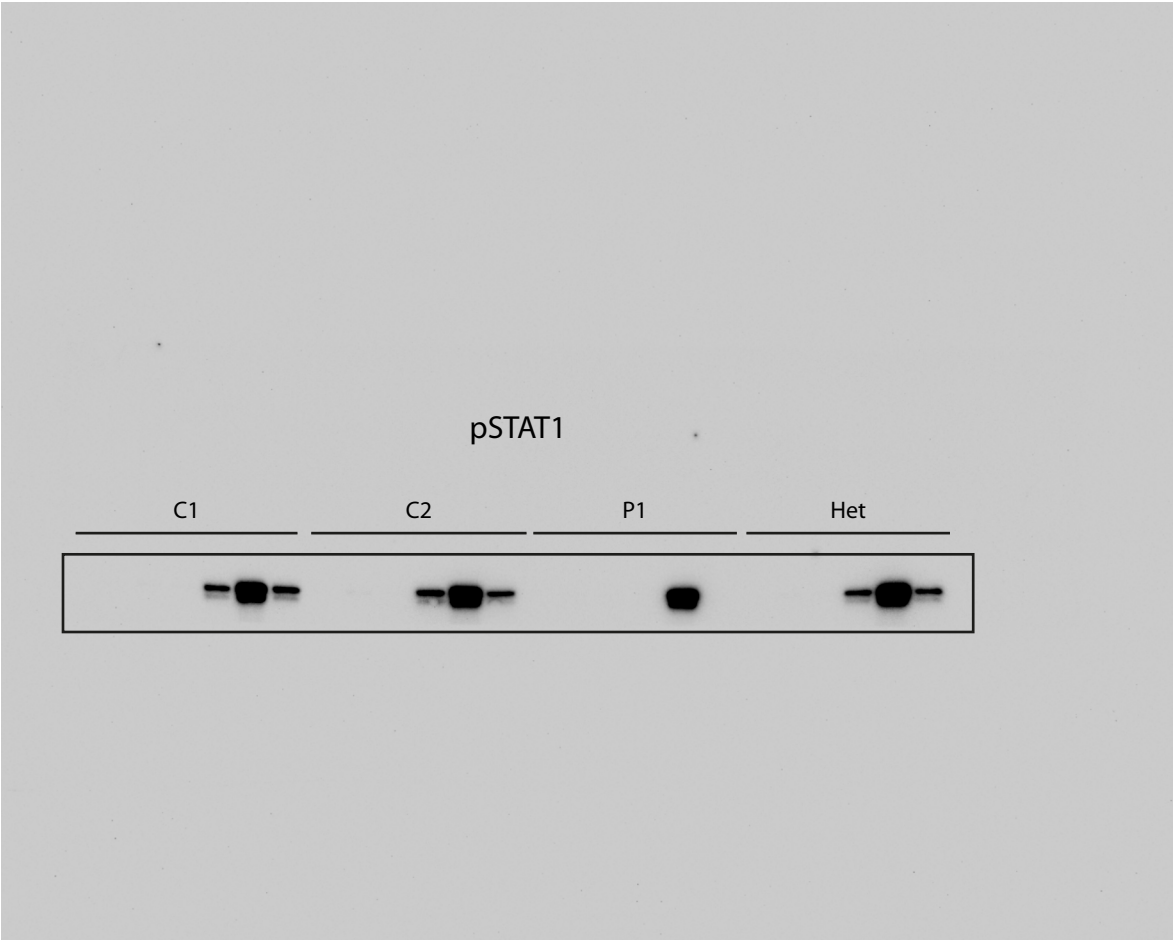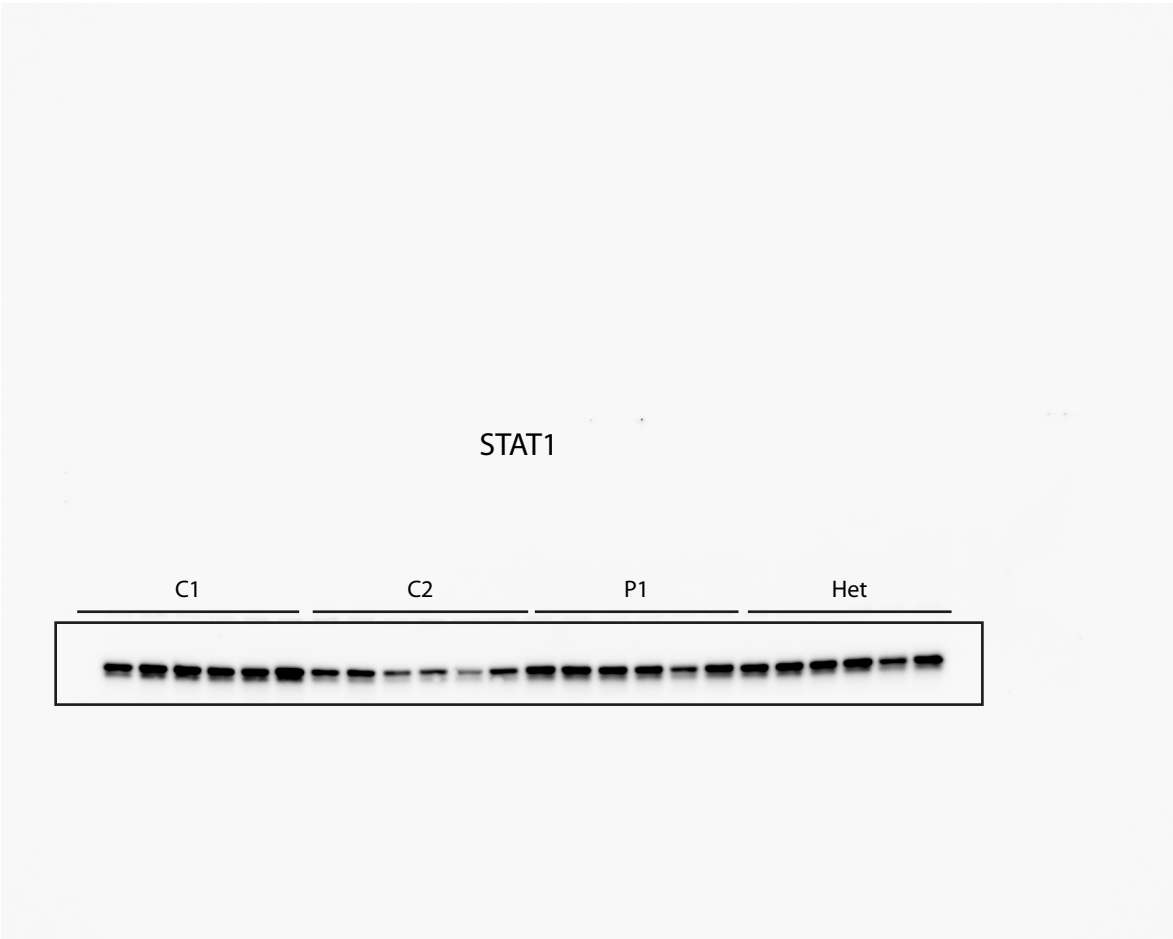

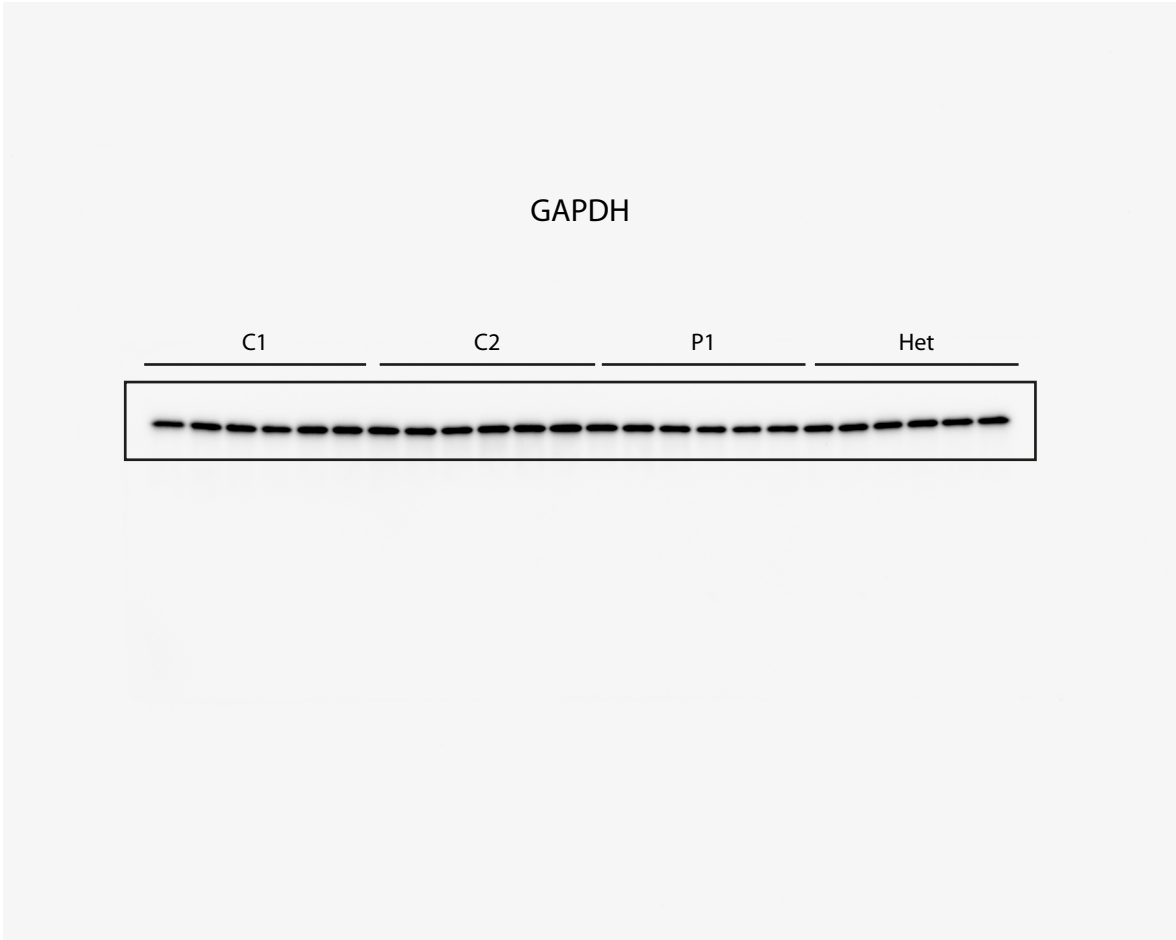

Source data: Figure 5A

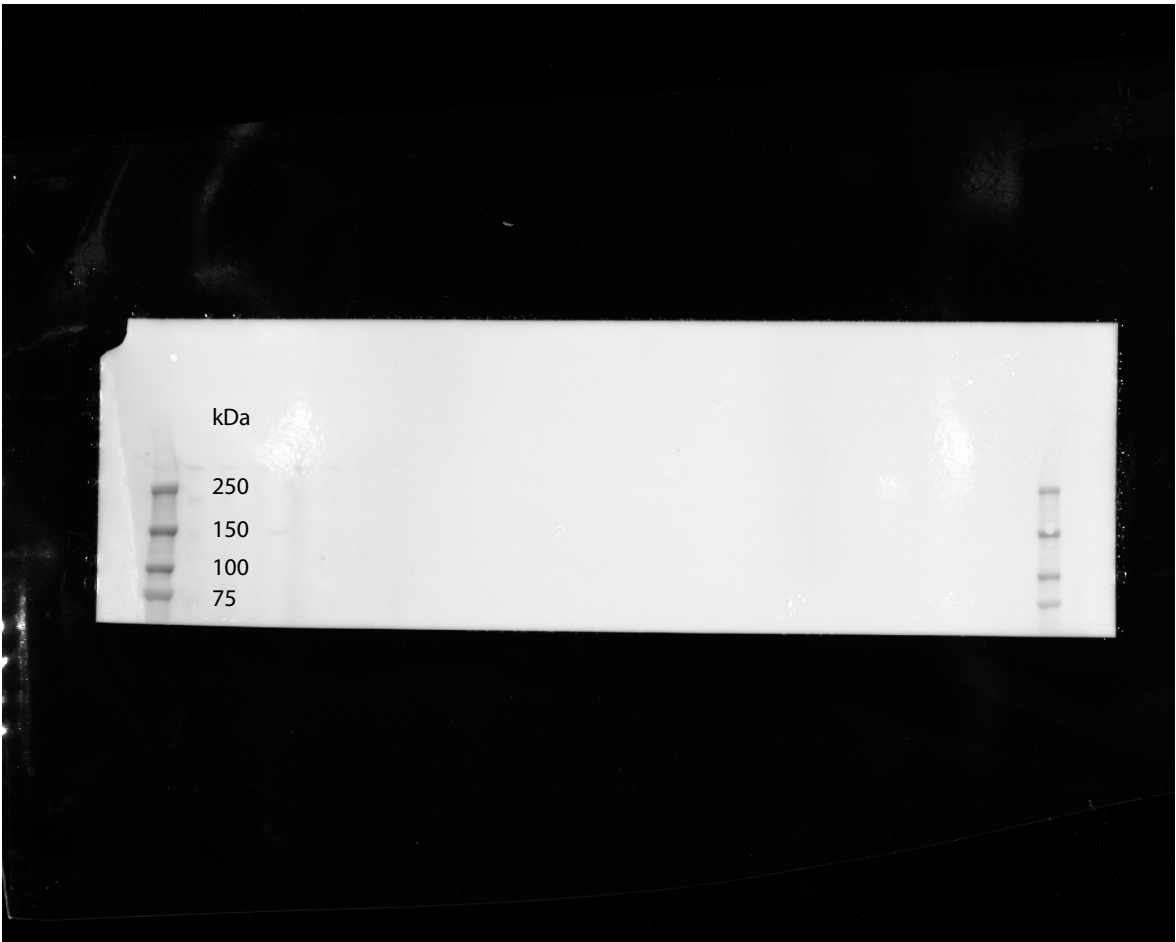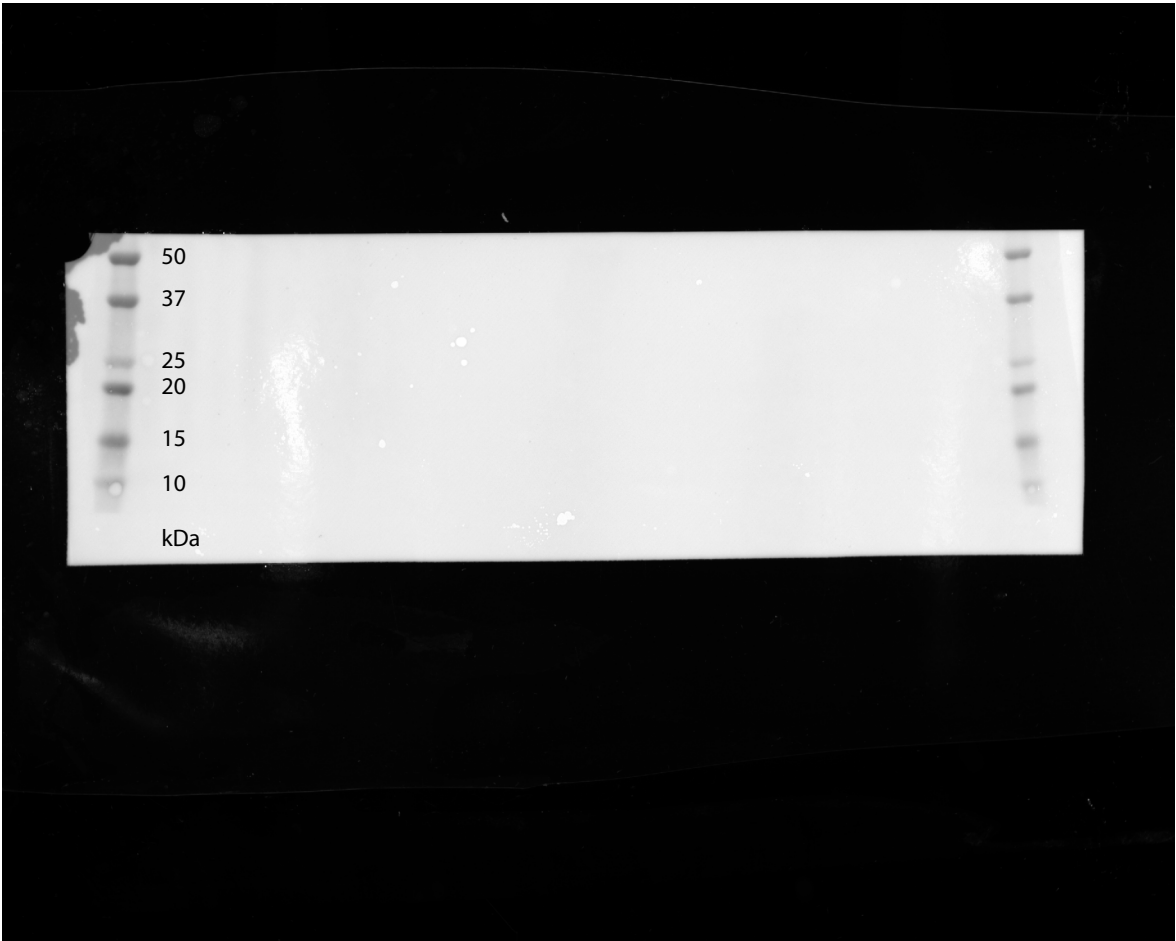

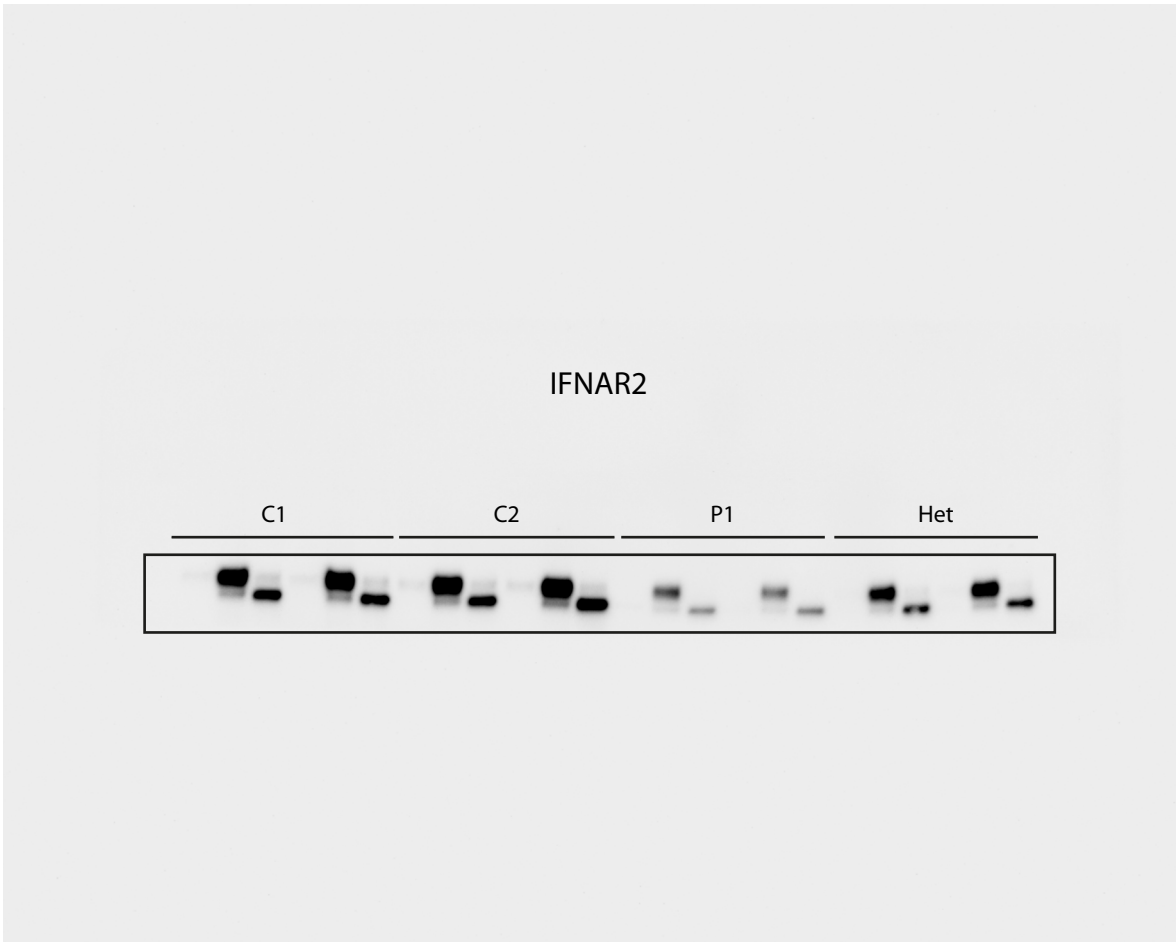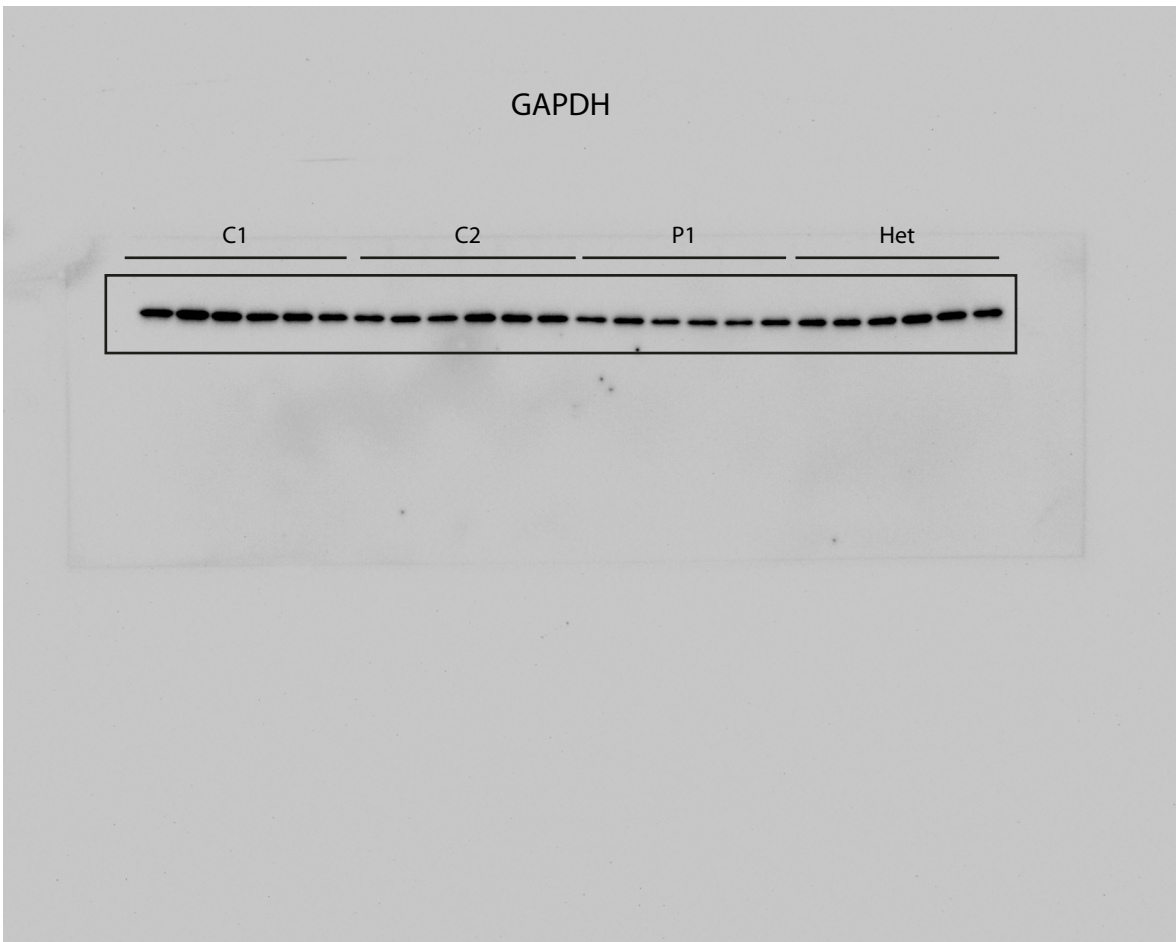

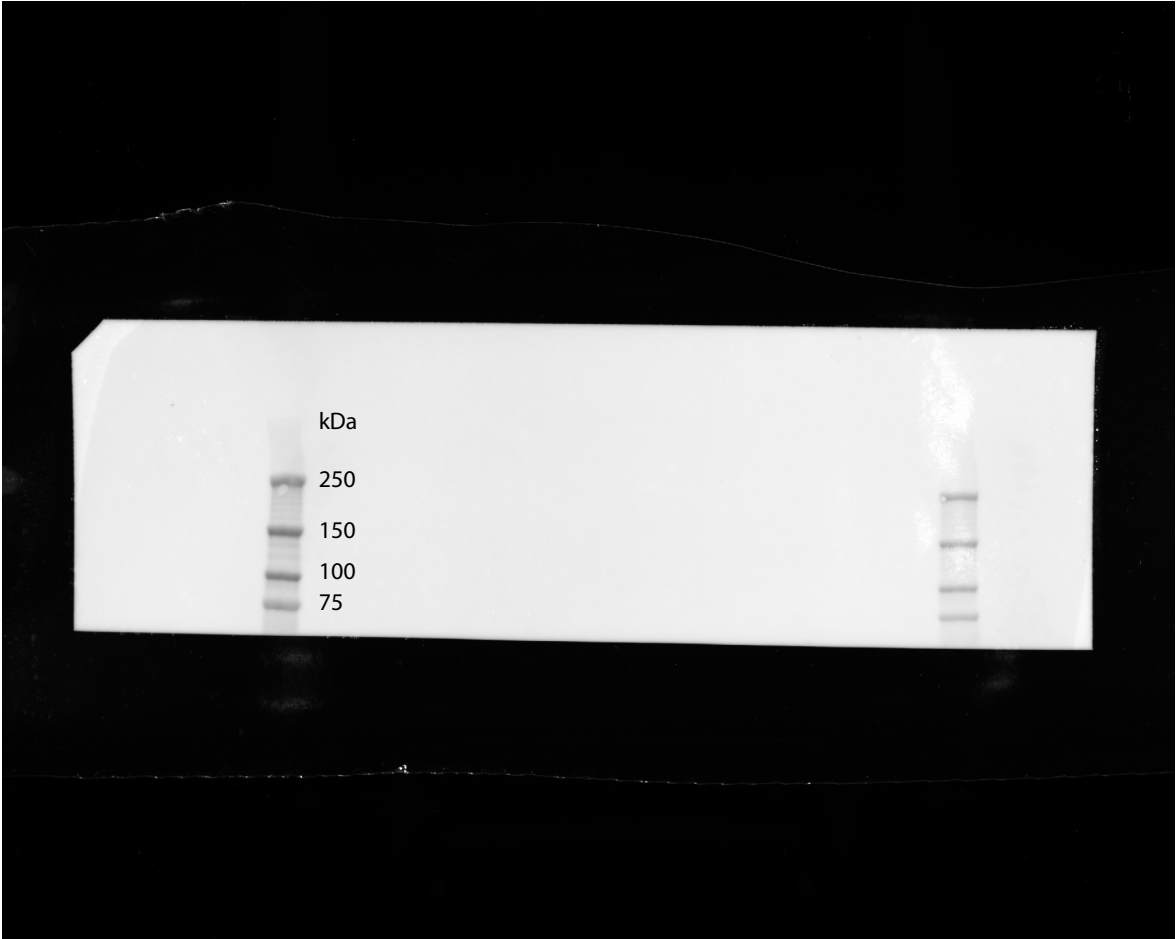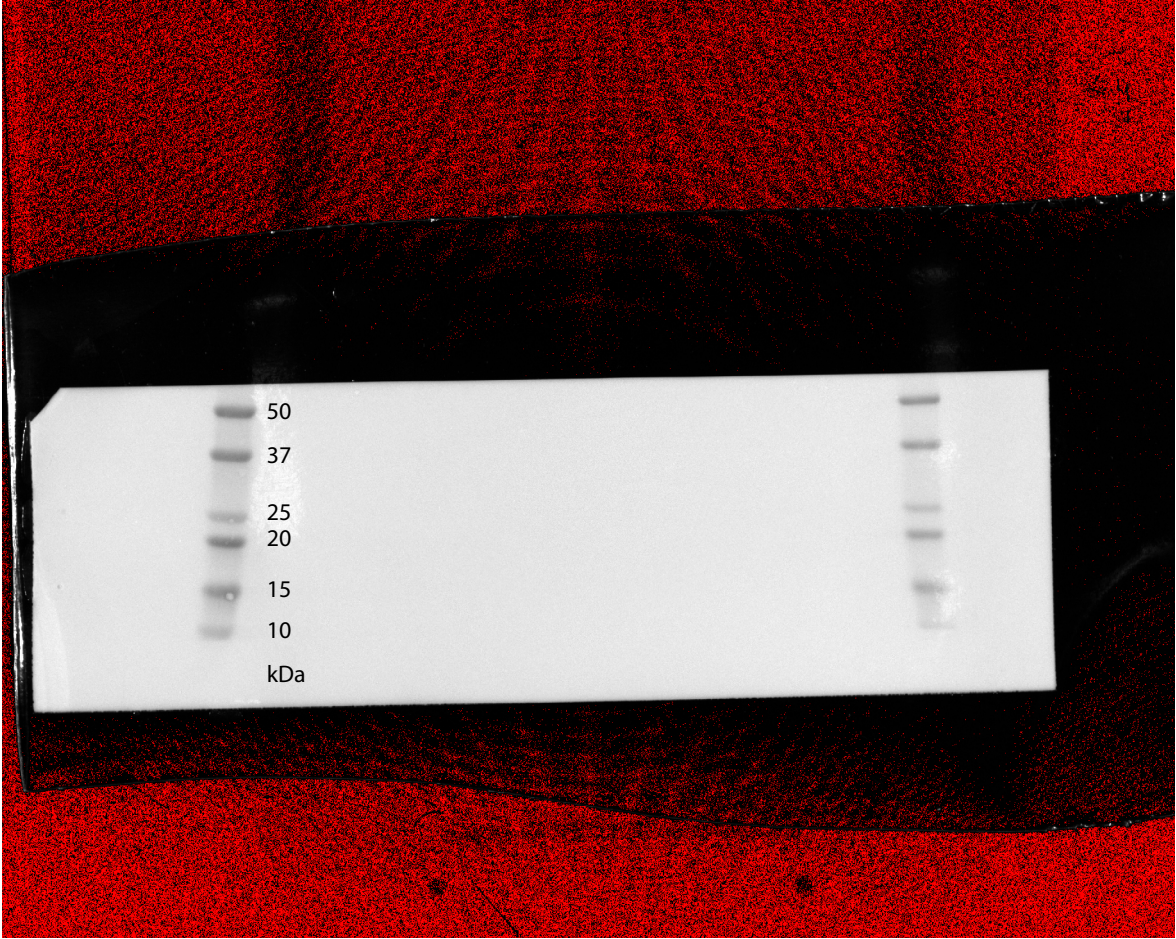

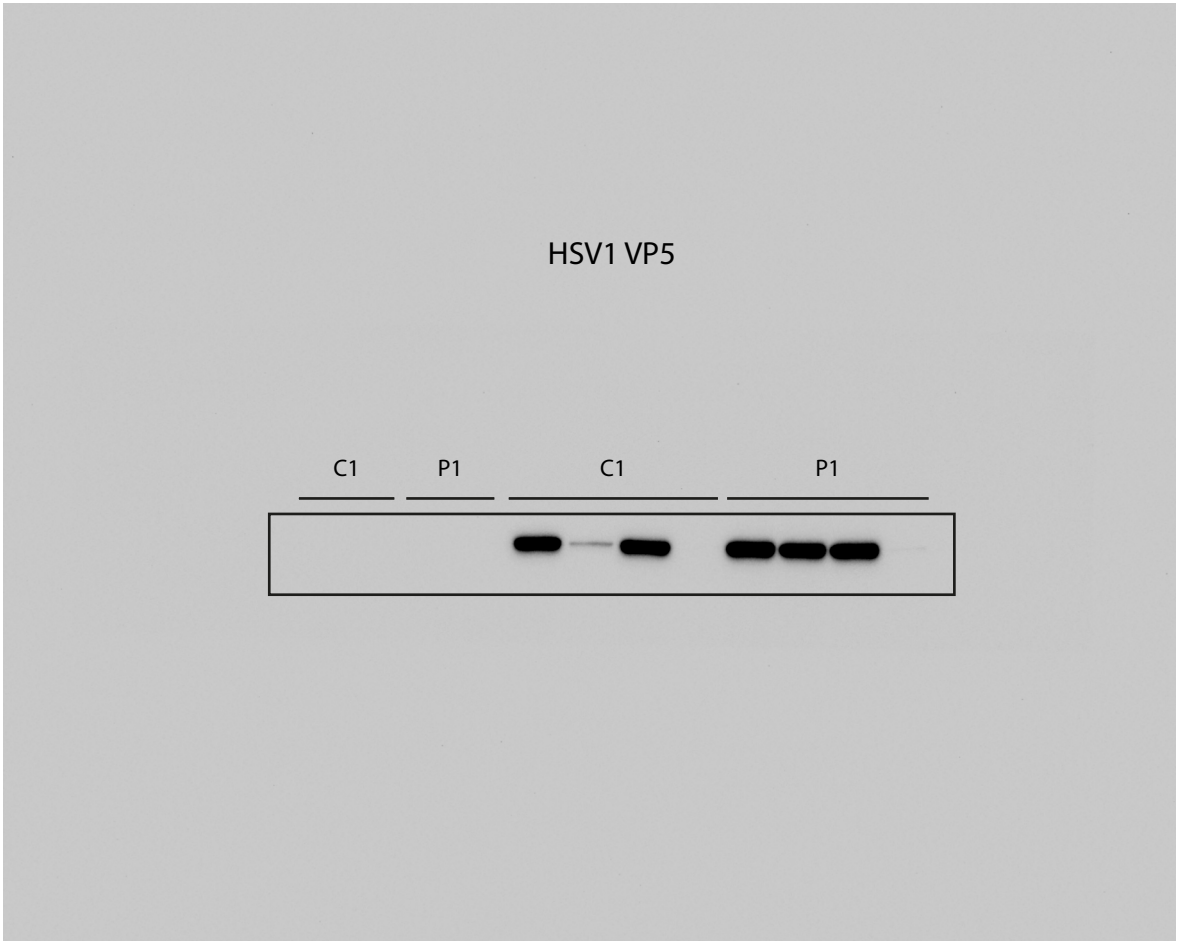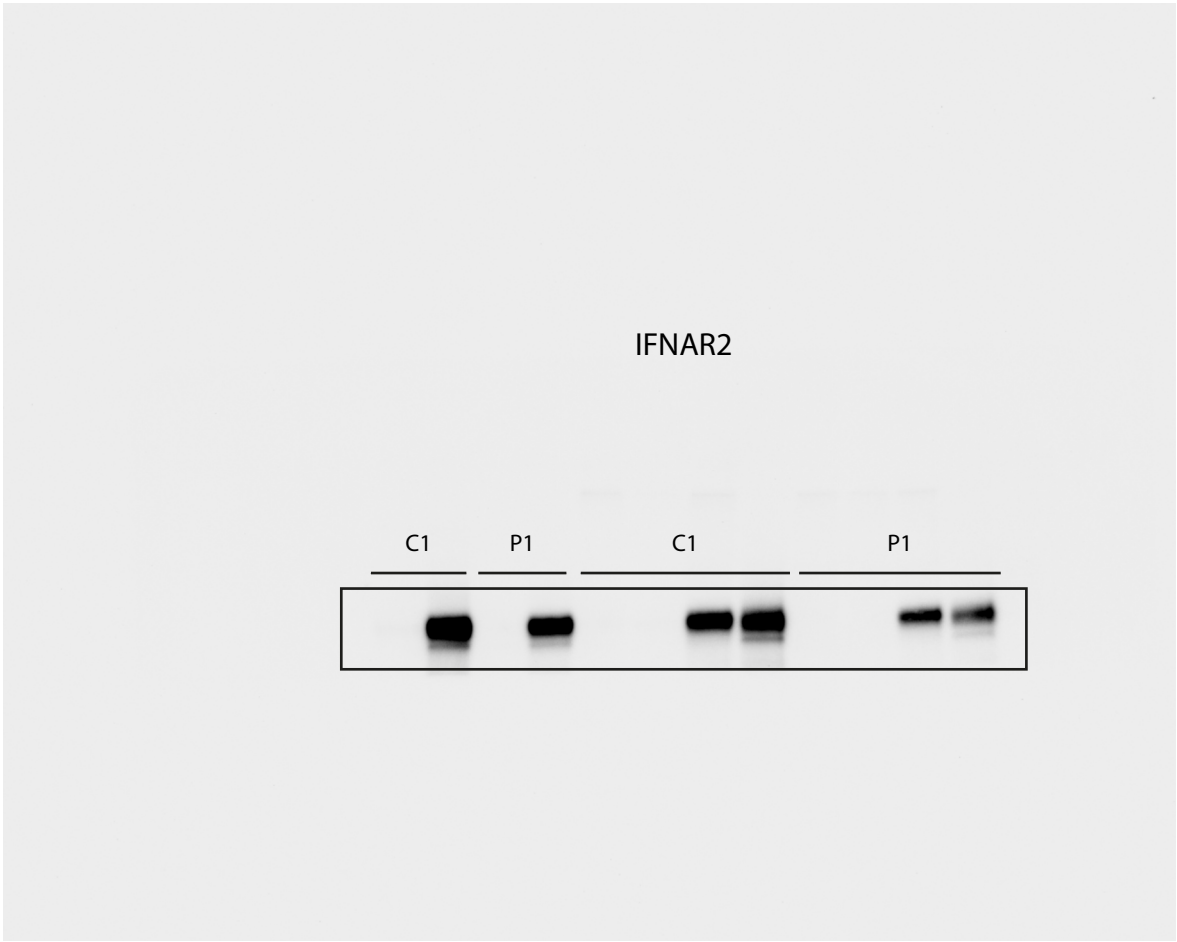

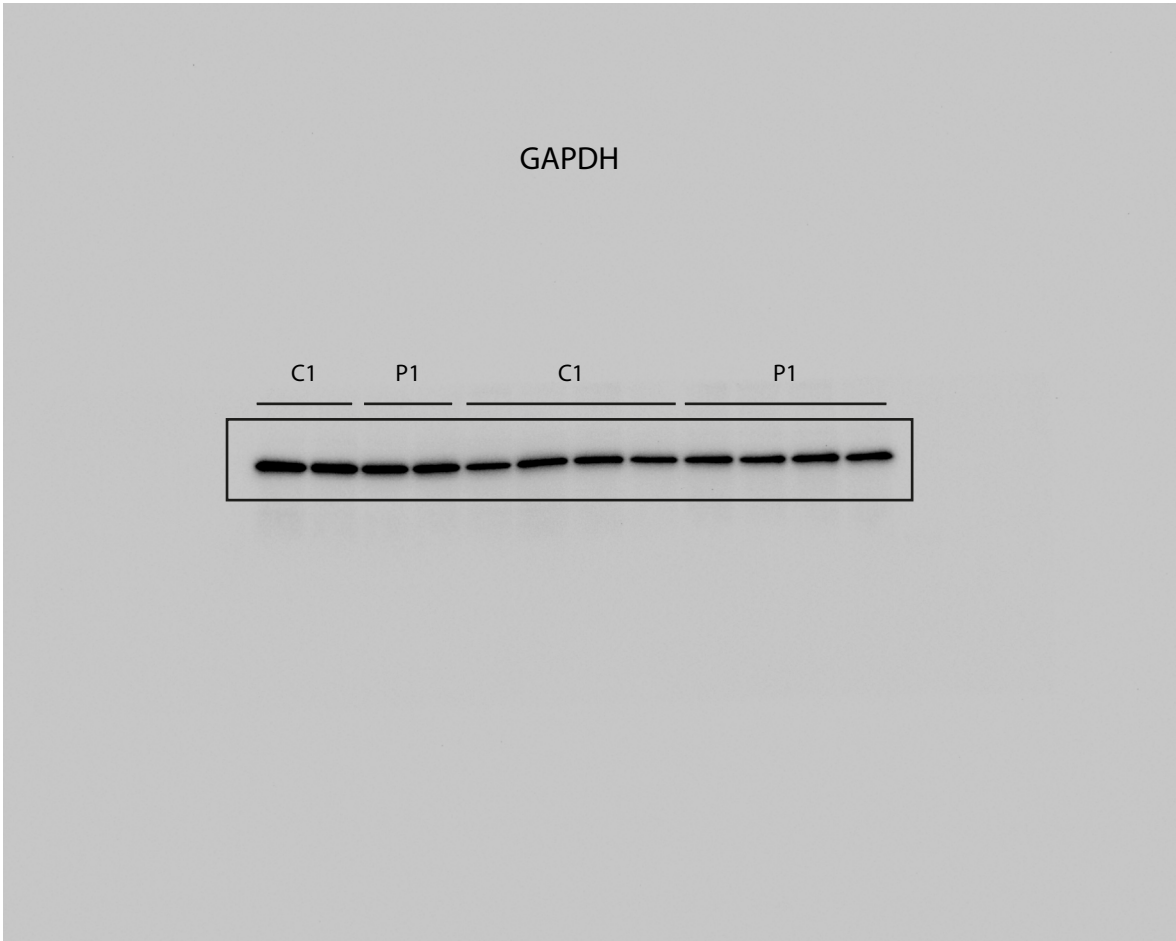

Supplement: SourceData F5 — contains original blots for Fig. 5. [file JEM_20212427_SourceDataF5.pdf]

pSTAT1 with ladder

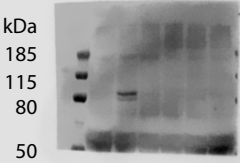

pSTAT1

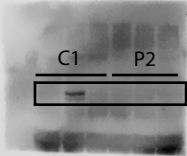

STAT1 with ladder

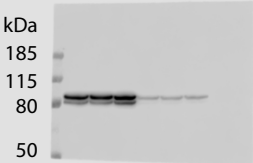

STAT1

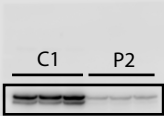

GAPDH with ladder

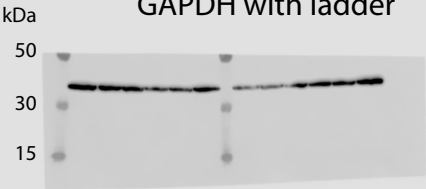

GAPDH

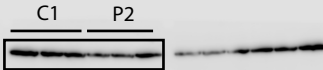

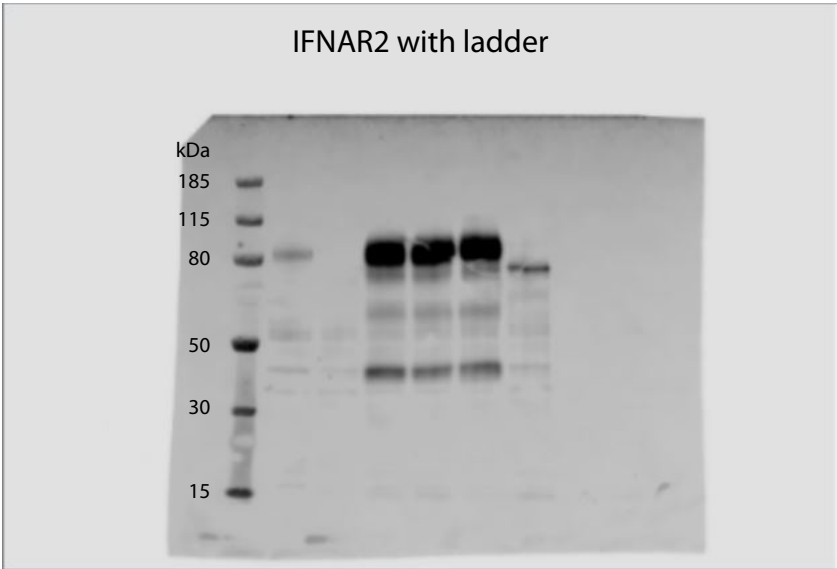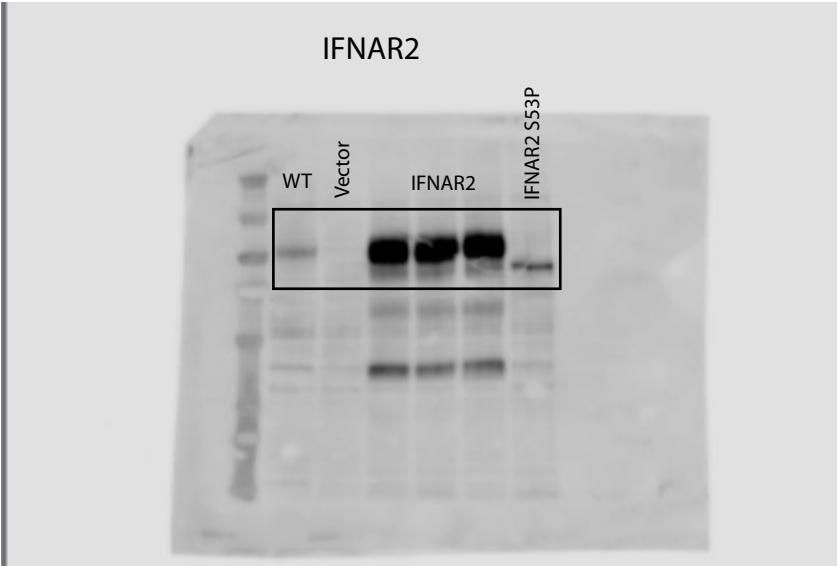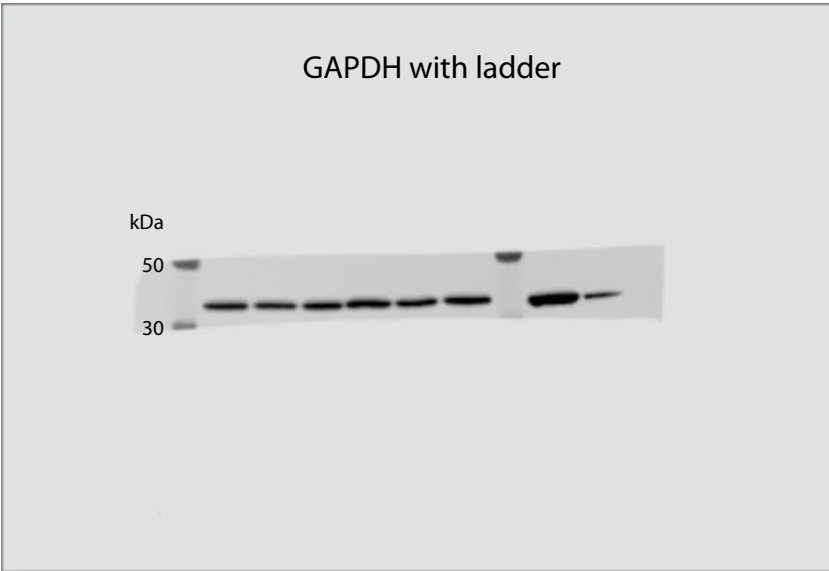

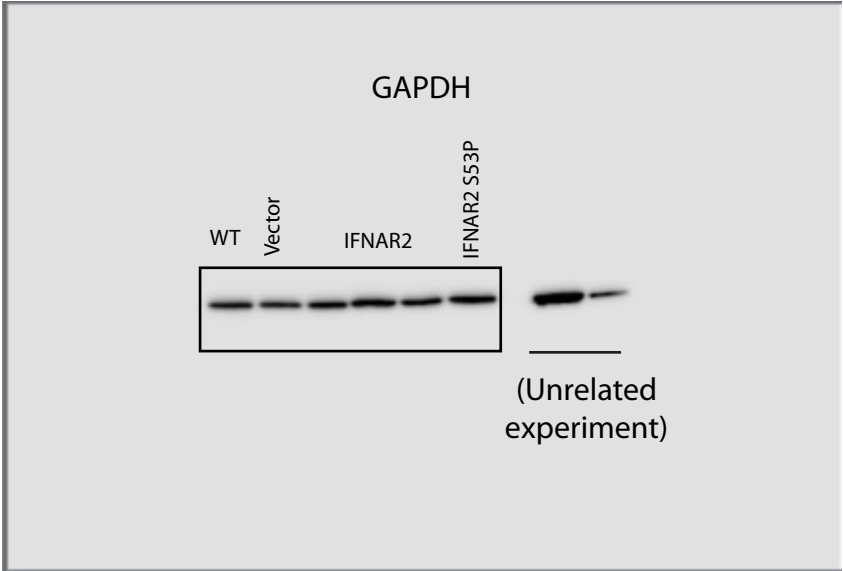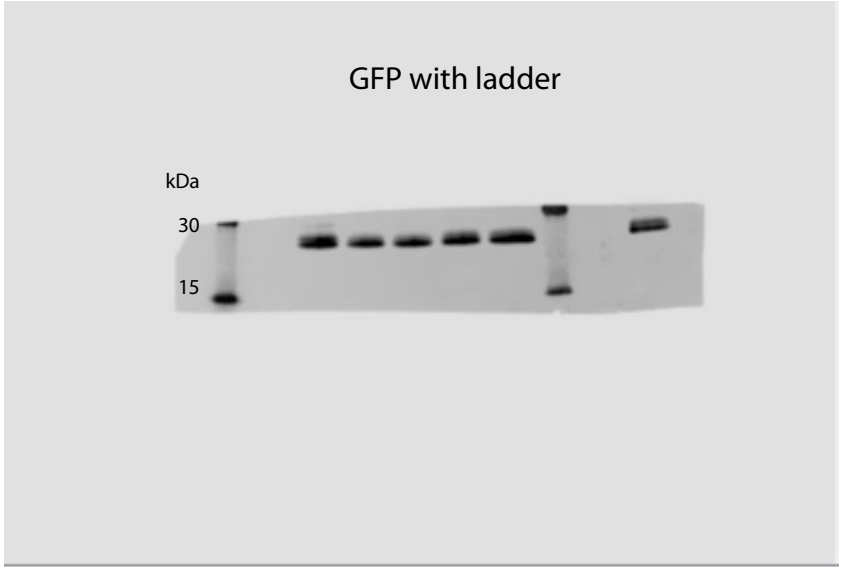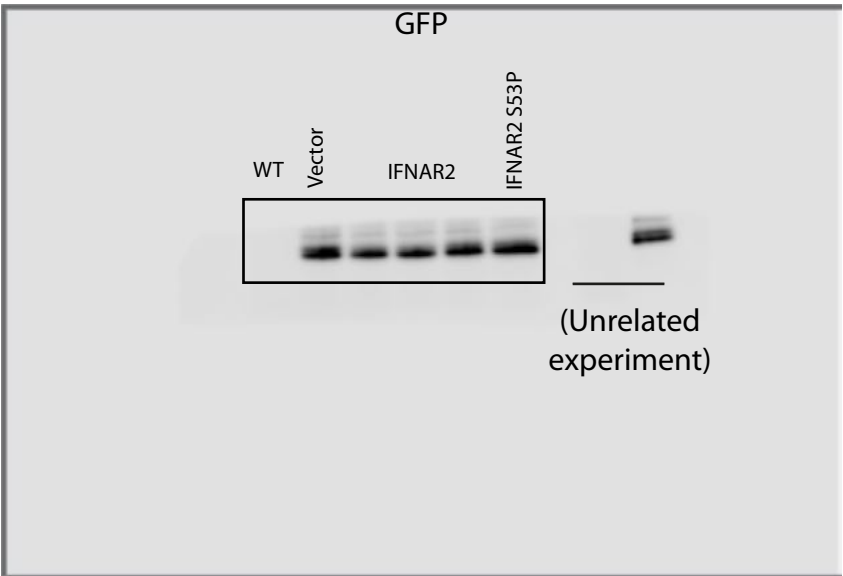

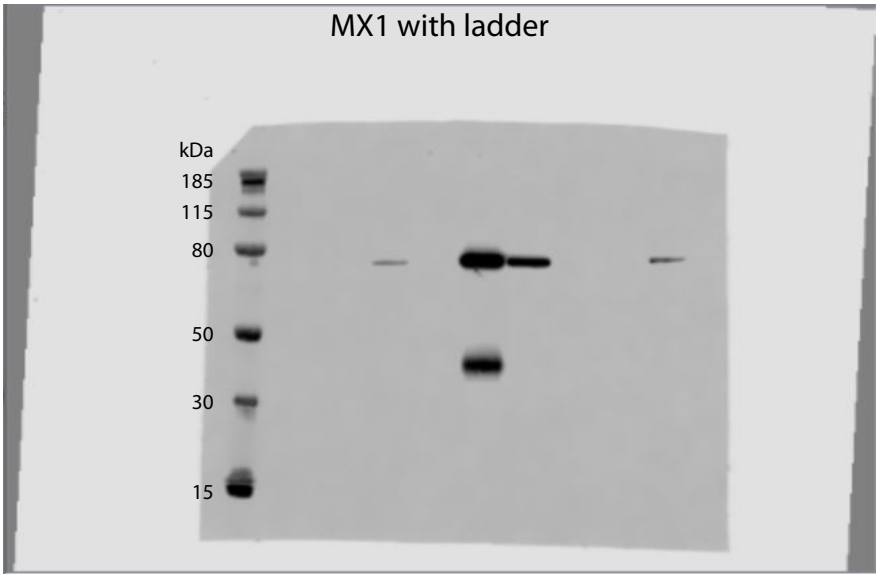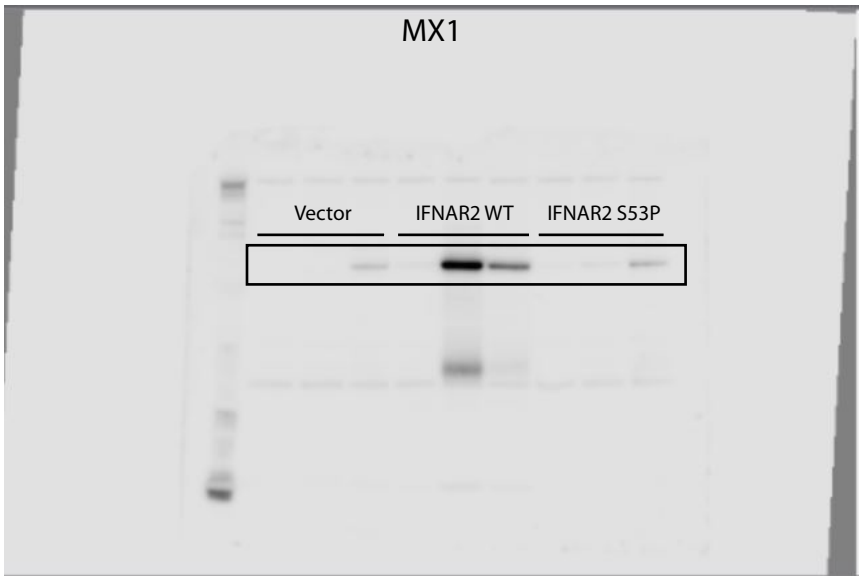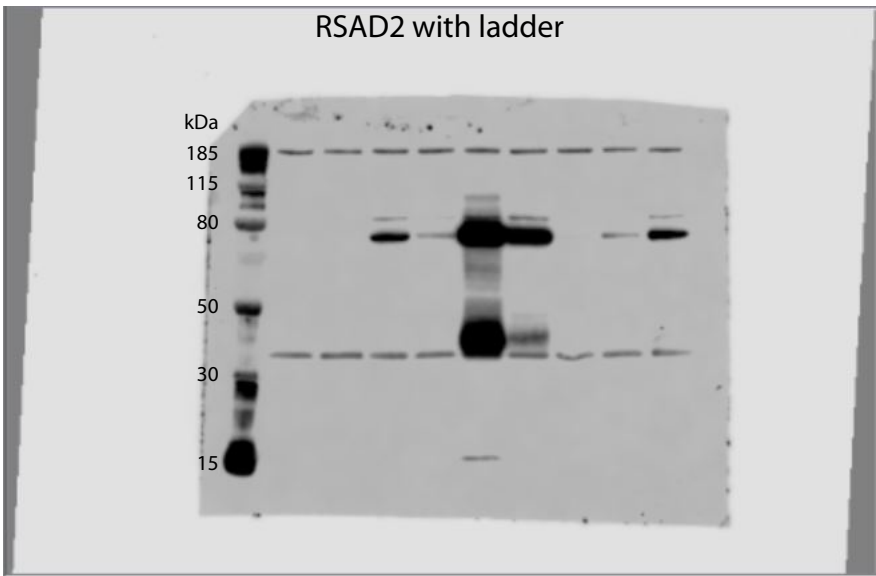

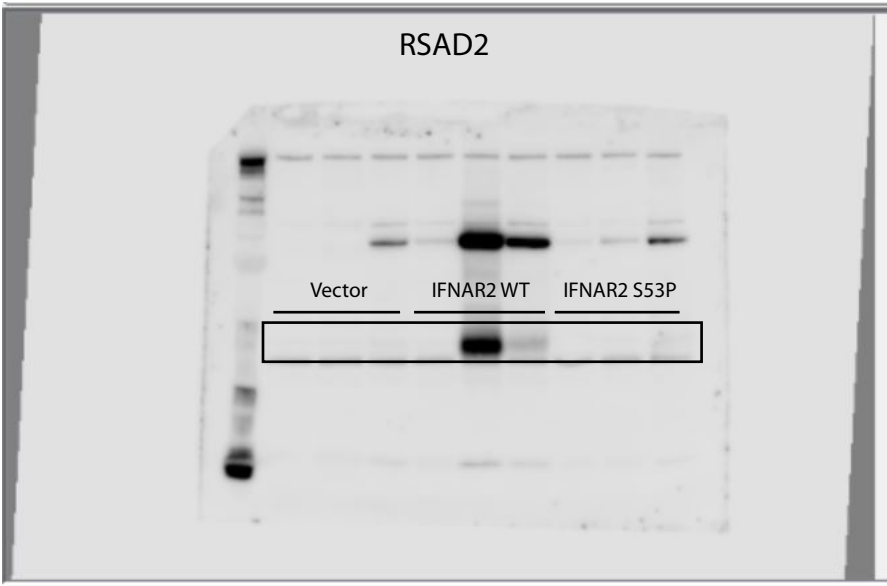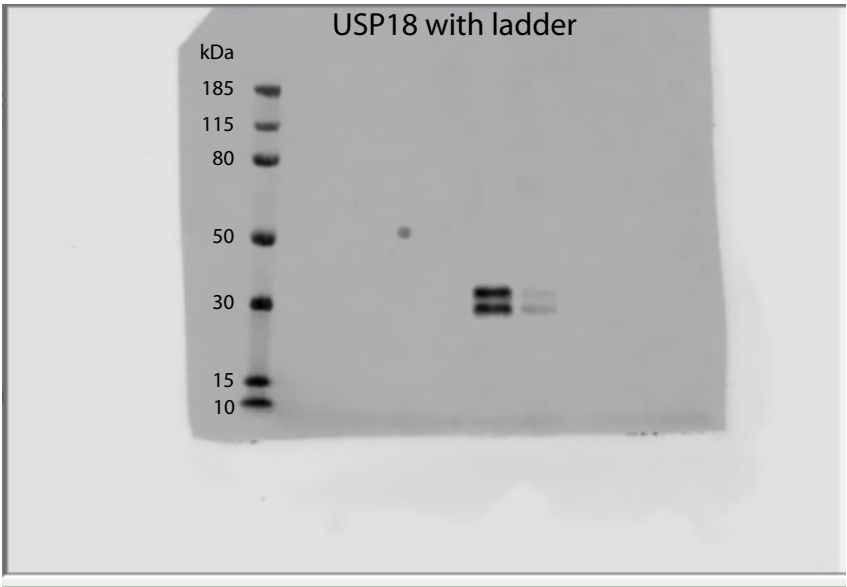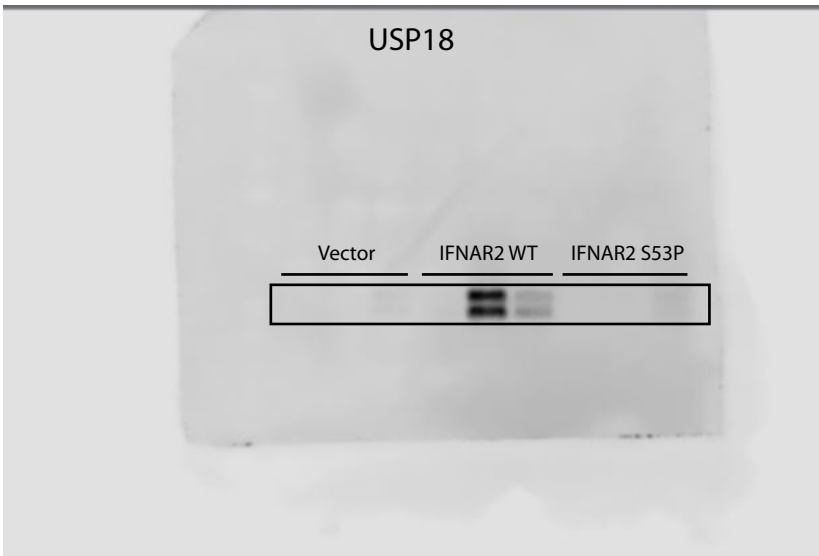

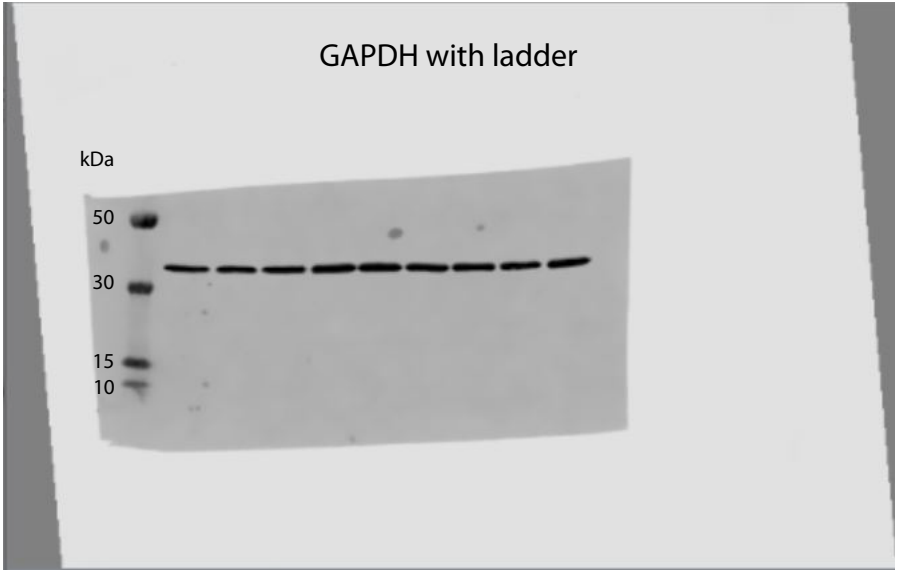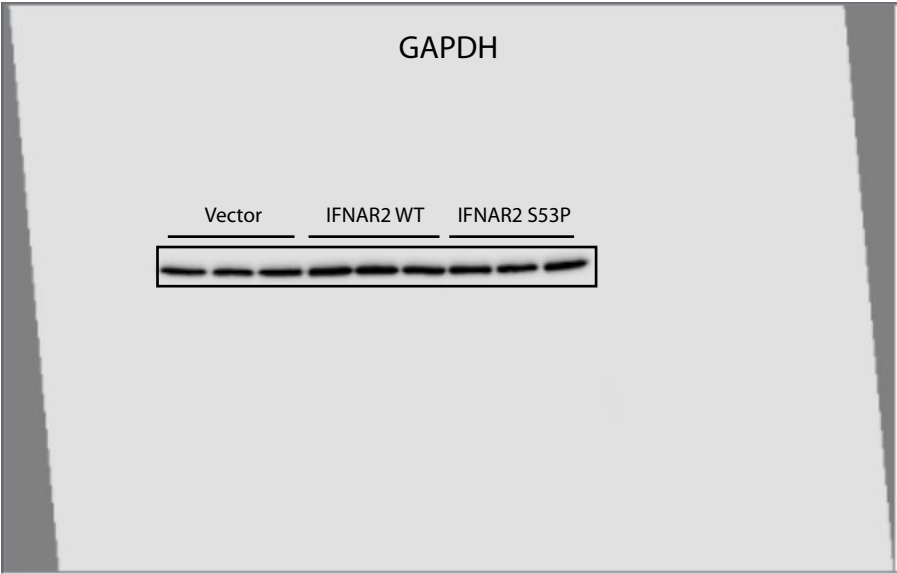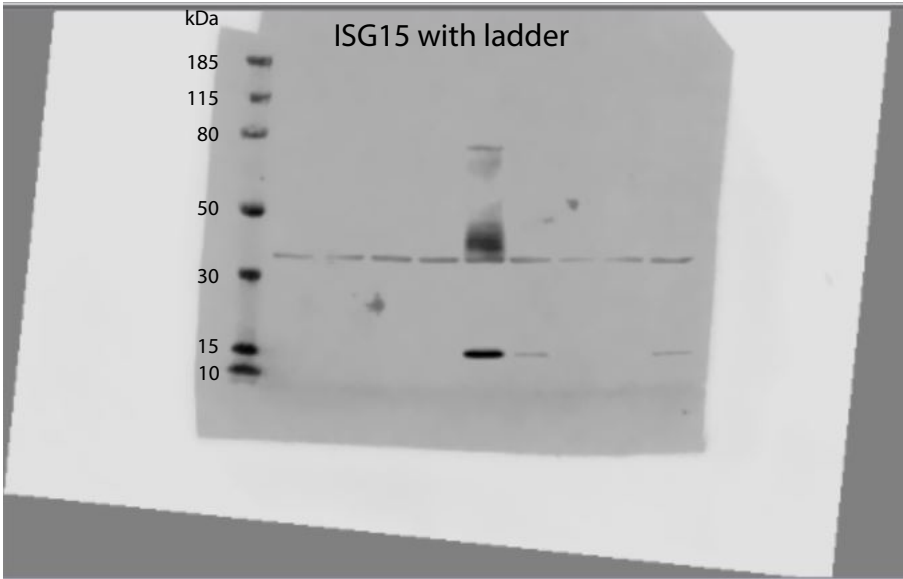

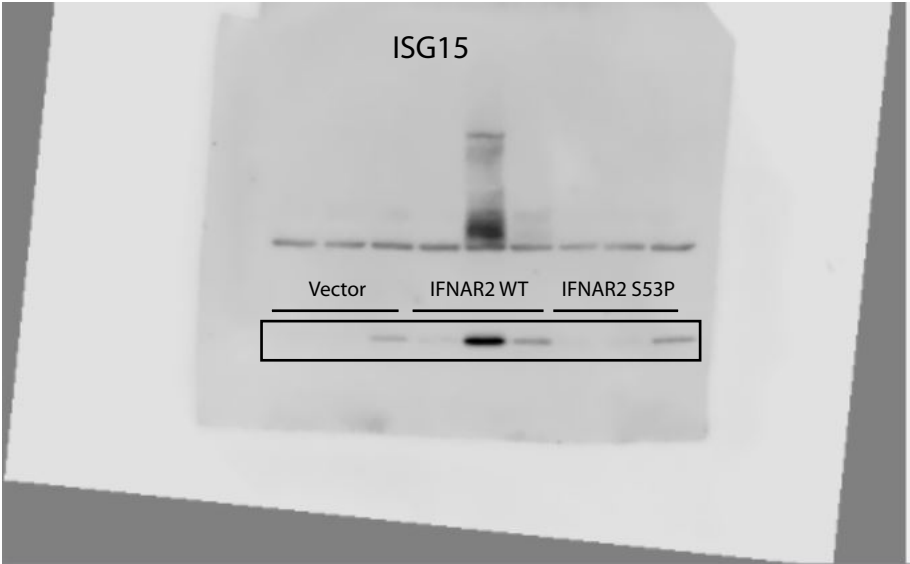

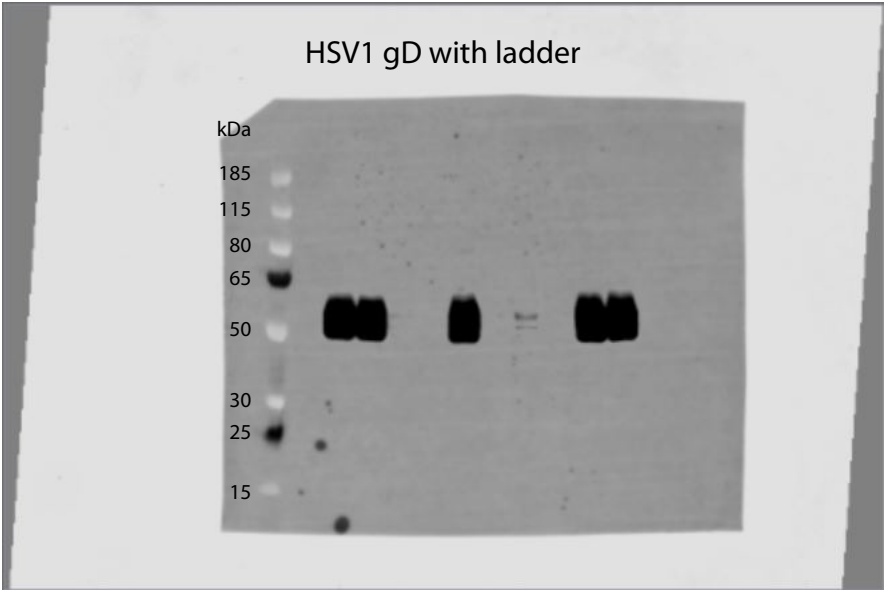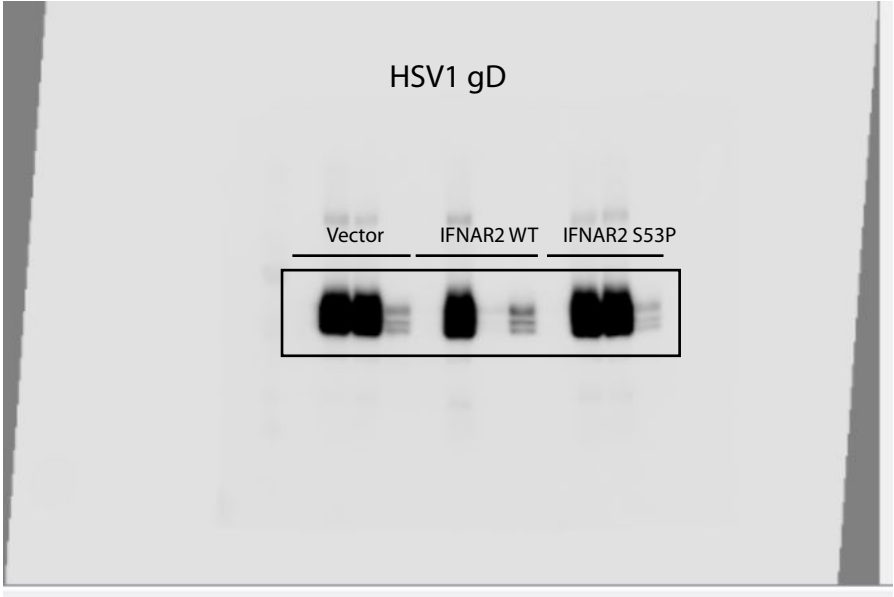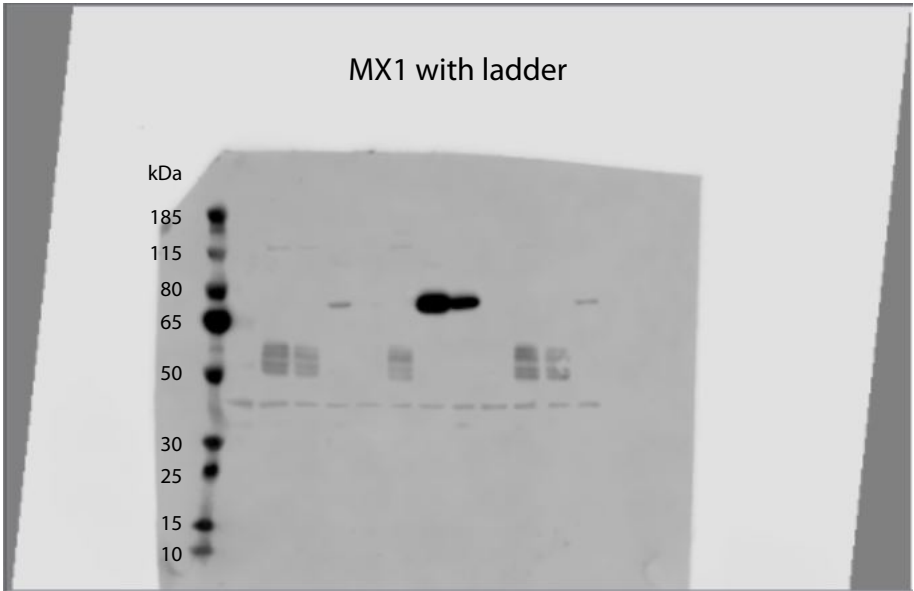

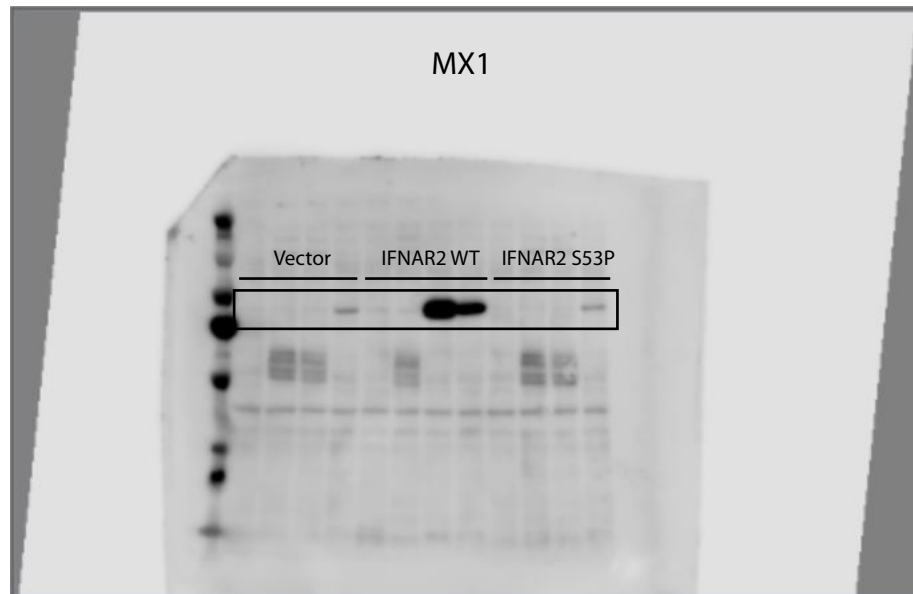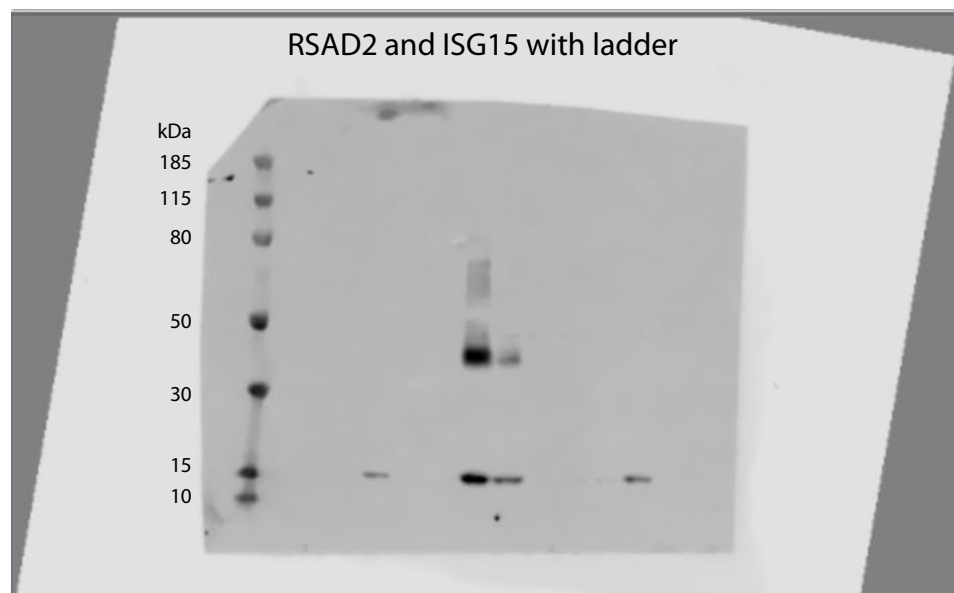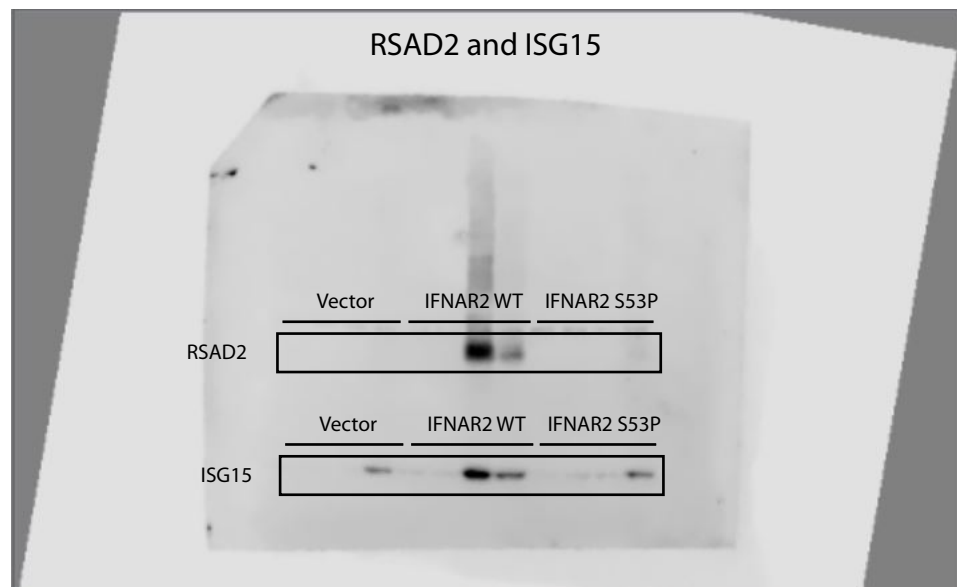

Source data: Figure S3E

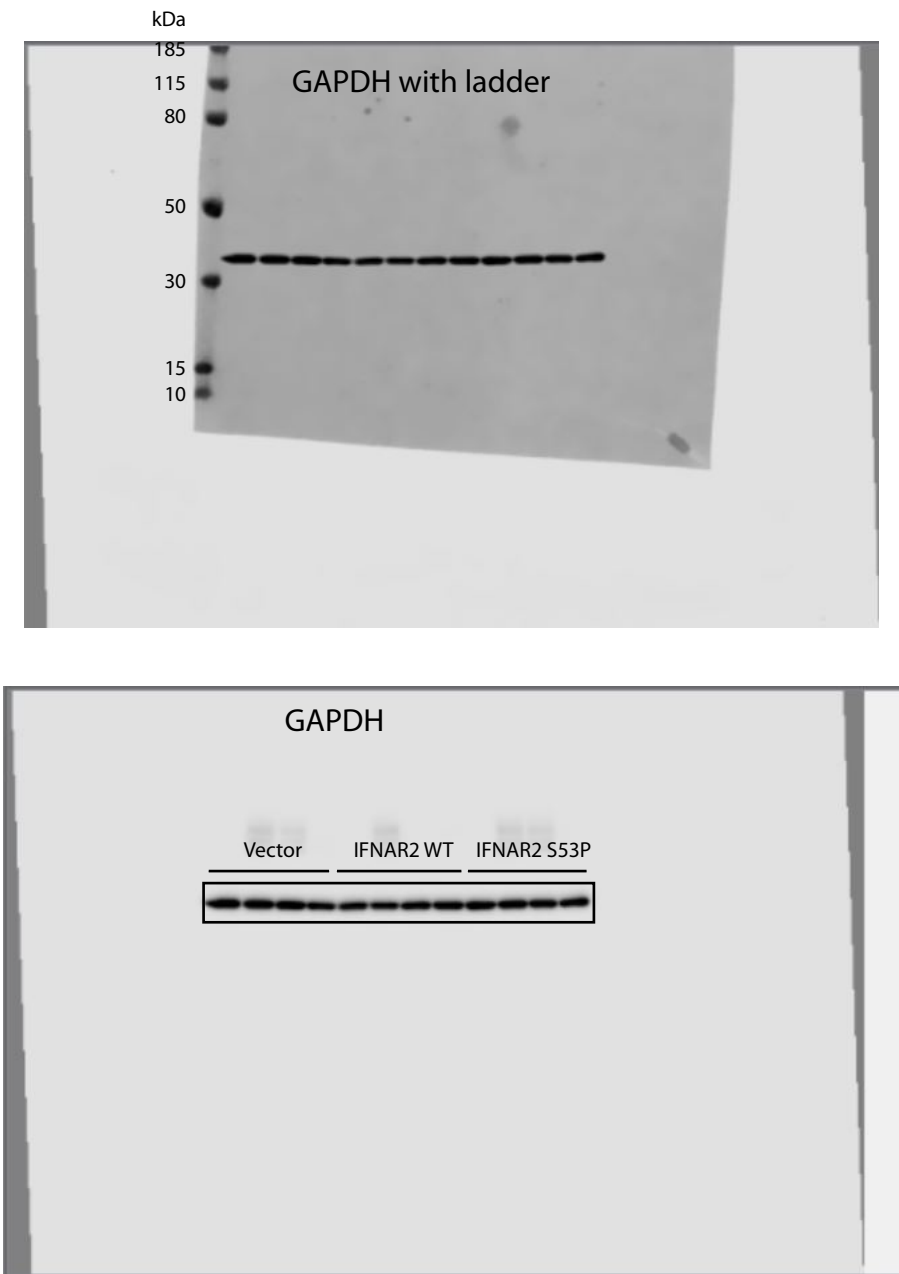

Supplement: SourceData FS3 — contains original blots for Fig. S3. [file JEM_20212427_SourceDataFS3.pdf]
